# An Unexpected Synthesis of 2-Sulfonylquinolines via Deoxygenative C2-Sulfonylation of Quinoline *N*-Oxides with Sulfonyl Chlorides

**DOI:** 10.3390/molecules29122863

**Published:** 2024-06-16

**Authors:** Wei Yang, Zhong-Ying Tian, Ying-Jun Lin, Long-Yong Xie

**Affiliations:** College of Chemistry and Bioengineering, Hunan University of Science and Engineering, Yongzhou 425100, China

**Keywords:** quinoline *N*-oxides, sulfonyl chlorides, sulfenylation, deoxygenative, metal free

## Abstract

A mild, efficient and practical protocol for the preparation of 2-sulfonylquinolines through CS_2_/Et_2_NH-induced deoxygenative C2-H sulfonylation of quinoline *N*-oxides with readily available RSO_2_Cl was developed. The reaction proceeded well under transition-metal-free conditions and exhibited a wide substrate scope and functional group tolerance. The preliminary studies suggested that the nucleophilic sulfonyl sources were generated in situ via the reaction of CS_2_, Et_2_NH and sulfonyl chlorides.

## 1. Introduction

**Scheme 1:**
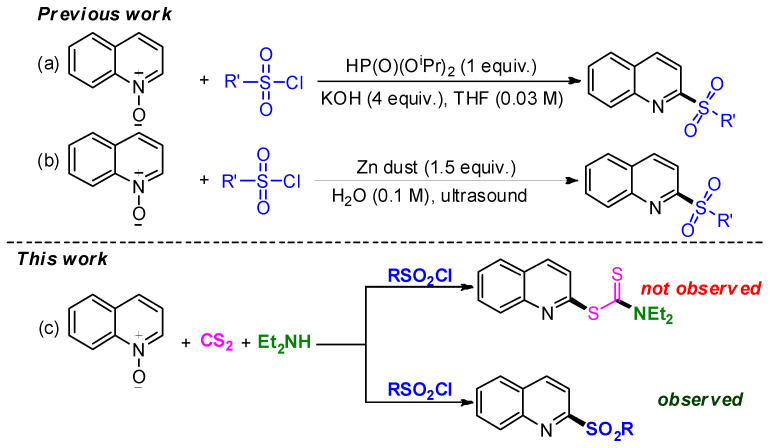
Deoxygenative C2-H sulfonylation of quinoline *N*-oxides with sulfonyl chlorides. (**a**) *H*-phosphonate-promoted deoxygenative C2-sulfonylation of quinoline *N*-oxides; (**b**) zinc-dust-mediated deoxygenative C2-sulfonylation of quinoline *N*-oxides; (**c**) CS_2_/Et_2_NH promoted deoxygenative C2-sulfonylation of quinoline *N*-oxides.

Quinolines are an important class of heterocyclic compounds that are widely present in natural products, medicine, functional materials and other fields [1,2,3,4,5,6]. Among them, 2-sulfonylquinolines exhibit diverse biological activities and serve as important building blocks for complex molecules [7,8,9]. Consequently, great efforts have been devoted to the synthesis of such compounds. In general, there are two main methods for 2-sulfonylquinolines: (1) the cross-coupling reactions of 2-haloquinolines with sulfonylating agents [10,11,12,13,14,15,16], which suffer from the pre-halogenation of the quinoline substrates; (2) the direct deoxygenative C2-H sulfonylation of quinoline *N*-oxides with various sulfonylating agents [17,18,19,20,21,22,23,24,25,26,27,28]. Most 2-haloquinolines are not commercially available and are often obtained via the deoxygenative halogenation of quinoline *N*-oxides with some toxic and irritant halogenating reagents (POX_3_, SOCl_2_, CX_4_, CCl_3_CN, etc.) [29,30,31]. Therefore, the later represents a much more efficient and practical method. In the past few years, great achievements have been gained in the C-H functionalization of quinoline *N*-oxides including alkylation [32], alkenylation [33,34,35], alkoxylation [36,37,38,39], amination [40,41,42,43], sulfenylation [44], cyanation [45,46], amidation [47,48], arylation [49,50], etc. [51,52,53,54]. Particularly, the deoxygenative C2-sulfonylation of quinoline *N*-oxides has been regarded as one of the most important methods for 2-sulfonylquinolines. Various sulfonylating reagents including RSO_2_Na [17,19,20,22,28], RSO_2_NHNH_2_ [23], RSO_2_Cl [26,27] and RSO_2_H [18,25] were utilized for the construction of sulfonylated quinolines. Among them, most RSO_2_Na, RSO_2_NHNH_2_ and RSO_2_H cannot be obtained from commercial sources and need to be prepared from cheap and readily available RSO_2_Cl as starting materials under alkaline conditions. Therefore, from the point of view of synthetic efficiency, directly using sulfonyl chlorides as sulfonylating reagents for 2-sulfonylquinolines represents a much more attractive strategy. In this context, several reports on the deoxygenative C2-sulfonylation of quinoline *N*-oxides with sulfonyl chlorides towards 2-sulfonylquinolines have been presented. For instance, in 2015, Zhao and co-workers first developed an *H*-phosphonate-promoted deoxygenative C2-sulfonylation of quinoline *N*-oxides with sulfonyl chlorides under strong base conditions (Scheme 1a) [27]. In 2017, our group reported an ultrasound-assisted and zinc-dust-mediated one-pot synthesis of various 2-sulfonylquinolines with quinoline *N*-oxides and sulfonyl chlorides as starting materials (Scheme 1b) [26]. Despite these advances, these reactions require the use of moisture-sensitive *H*-phosphonate and large amounts of a strong base or the stoichiometric transition metals, which may result in metal residues of the finished products. Therefore, the development of many more mild and efficient methods for the synthesis of 2-sulfonylquinolines under transition-metal-free conditions is still in high demand. 

With our continuing interest in the deoxygenative C2-H functionalization of quinoline *N*-oxides [25,55,56,57,58], herein, we report on an unexpected method for 2-sulfonylquinolines via CS_2_/Et_2_NH-promoted deoxygenative C2-sulfonylation of quinoline *N*-oxides with commercially available sulfonyl chlorides (Scheme 1c). In this reaction, sulfonyl chlorides act as both sulfonyl sources and electrophilic reagents utilized for activating quinoline *N*-oxides.

## 2. Results and Discussion

In our initial study, we speculated that diethylcarbamodithioic acid (Et_2_NCS_2_H) generated in situ through the reaction of CS_2_ and Et_2_NH could serve as a sulfur-containing nucleophilic reagent, which might further attack quinoline *N*-oxide at the C2 position in the presence of TsCl as an electrophilic activating reagent. With this in mind, a reaction of quinoline *N*-oxide (**1a**) and TsCl (**2a**) was carried out with 1.2 equivalents of carbon disulfide and 1.5 equivalents of diethylamine at room temperature for approximately 0.5 h using THF as the reaction solvent. Beyond our expectation, quinolin-2-yl diethylcarbamodithioate **3aa’** was not detected in the reaction mixture, but 2-tosylquinoline **3aa** was isolated in 55% yield (Table 1, entry 1). To further optimize the reaction conditions of this unexpected sulfonylation reaction, the influence of different solvents on the reaction was explored, among which dichloromethane was identified as the most effective solvent, producing product **3aa** in 67% yield (entry 2), while other investigated solvents resulted in lower yields (entries 3–7). Among the amines tested, dimethylamine produced **3aa** in 43% yield (entry 8), while diisopropylamine (entry 9), morpholine (entry 10) and piperidine (entry 11) produced **3aa** in only around 10% yields. Particularly, triethylamine was found to be unsuitable for the present transformation (entry 12). Increasing the amount of **2a** did not significantly impact the yield of **3aa** (entries 13 and 14). However, a decrease to 1.5 equivalents resulted in a 53% yield of **3aa** (entry 15). Moreover, increasing the amount of diethylamine to 2 equivalents improved the yield of **3aa** to 79% (entry 16), and further optimization by increasing the amount of carbon disulfide to 1.5 equivalents increased the yield to 84% (entry 17). Control experiments confirmed that the presence of carbon disulfide and secondary amine is essential for the formation of the sulfonylation products (entries 19 and 20).

**Scheme 2:**
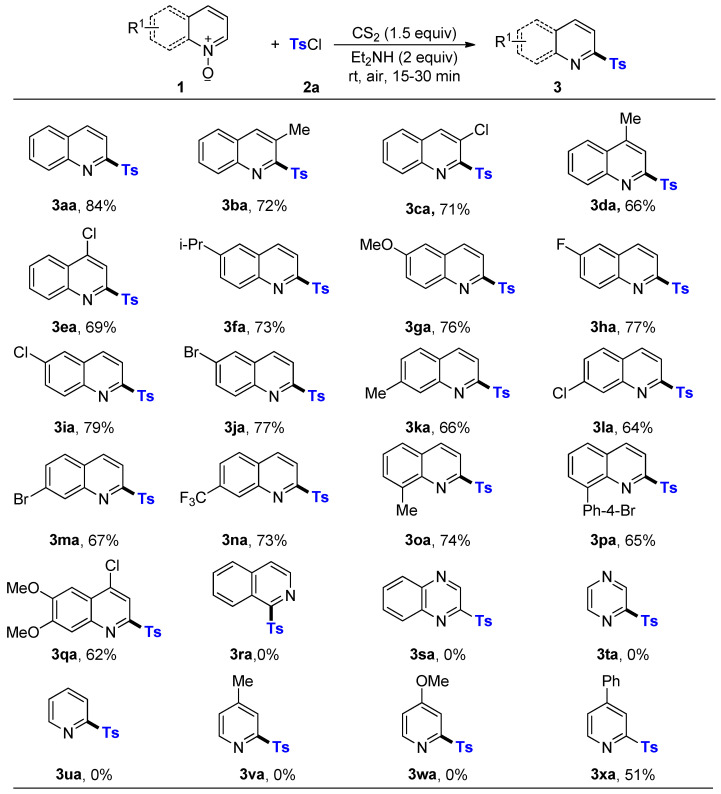
Preparation of **3aa**–**3xa**. Conditions: **1** (0.3 mmol), **2a** (0.6 mmol), CS_2_ (0.45 mmol), Et_2_NH (0.6 mmol), DCM (3 mL), air, rt, 15–30 min. Isolated yields are given based on **1**.

After obtaining the optimal reaction conditions (Table 1, entry 17), we proceeded to investigate the substrate scope and limitations with respect to various heterocyclic *N*-oxides, as illustrated in Scheme 2. We found that a range of quinoline *N*-oxides were suitable for the current transformation and produced the corresponding products in 62%–84% yields (**3aa**–**3pa**). It is worth noting that some important functional groups, such as alkyl (**3ba**, **3da**, **3fa**, **3ka** and **3oa**), methoxy (**3ga**), F (**3ha**), Cl (**3ca**, **3ea, 3ia** and **3la**), Br (**3ja** and **3ma**), and aryl (**3pa**) groups, at different positions on the quinoline rings were compatible with the reaction. In addition, tri-substituted quinoline *N*-oxide **1q** also reacted well with TsCl and provided the expected product **3qa** in 62% yield. However, isoquinoline *N*-oxide (**3ra**), quinoxaline *N*-oxide (**3sa**), pyrazine *N*-oxide (**3ta**) and several substituted pyridine *N*-oxides (**3ua–3wa**) did not yield the desired products, which was likely due to their lower activities in contrast to quinoline *N*-oxides, which can be speculated from the below-mentioned possible mechanism that nucleophilic addition to a pyridine *N*-oxide would result in complete loss of aromaticity whereas with quinoline *N*-oxide, there would only be a partial loss of aromaticity. Interestingly, 4-phenylpyridine *N*-oxide as a potential substrate also produced the expected product **3xa** in 51% yield.

**Scheme 3:**
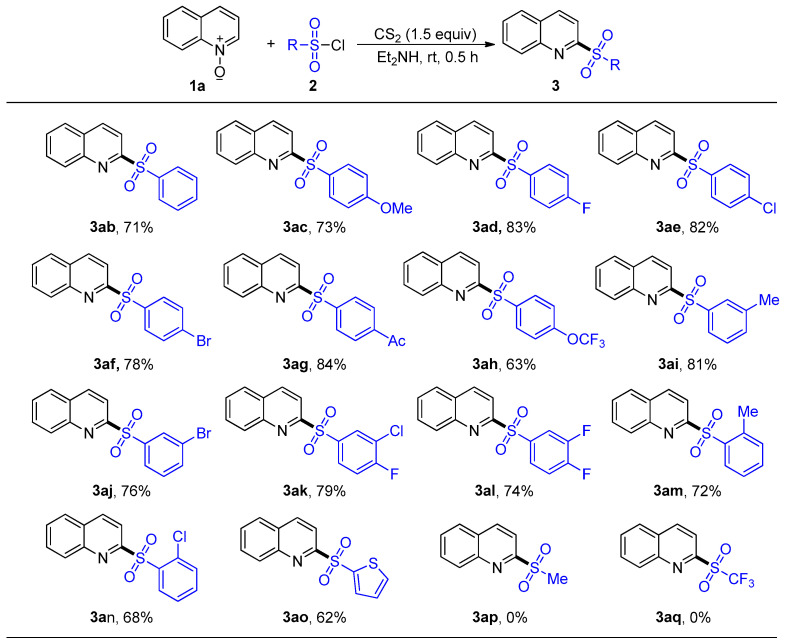
Preparation of **3ab–3aq**. Conditions: **1a** (0.3 mmol), **2** (0.6 mmol), CS_2_ (0.45 mmol), Et_2_NH (0.6 mmol), DCM (3 mL), air, rt, 15–30 min. Isolated yields are given based on **1a**.

We then turned our attention to investigate the scope of sulfonyl chlorides, as shown in Scheme 3. We found that various aryl sulfonyl chlorides bearing electron-donating, electron-withdrawing or steric hindered functional groups at the different positions of the phenyl rings all reacted well with **1a** to produce the corresponding products **3ab**–**3an** in 63–84% yields. The reaction was compatible with a range of valuable substitutions, such as alkyl (**3ai** and **3am**), methoxy (**3ac**), halogen atoms (**3ad**–**3af**, **3aj** and **3an**), acetyl (**3ag**) and trifluoromethoxy (**3ah**). In addition, di-substituted aryl sulfonyl chlorides were found efficient for the reaction and produced the desired products **3ak** and **3al** in 79 and 74% yields, respectively. To our delight, thiophene-2-sulfonyl chloride was also a suitable substrate to produce the target product **3ao** in 62% yield. However, aliphatic sulfonyl chlorides including methanesulfonyl chloride and trifluoromethanesulfonyl chloride failed to produce the corresponding products (**3ap** and **3aq**), presumably owing to their relatively poor abilities for activating quinoline *N*-oxides compared with aryl sulfonyl chlorides.

**Scheme 4:**
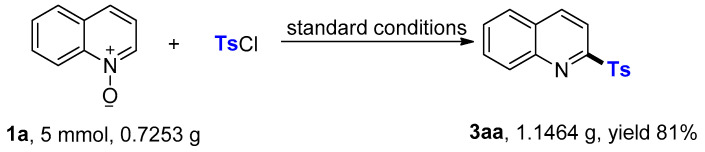
Gram-scale synthesis of **3aa**.

To demonstrate the synthetic application of this method, a gram-scale experiment between quinoline *N*-oxide **1a** (5 mmol, 0.7253 g) and TsCl **2a** was carried out under standard conditions. As anticipated, the expected product **3aa** was isolated in 81% yield (Scheme 4).

**Scheme 5:**
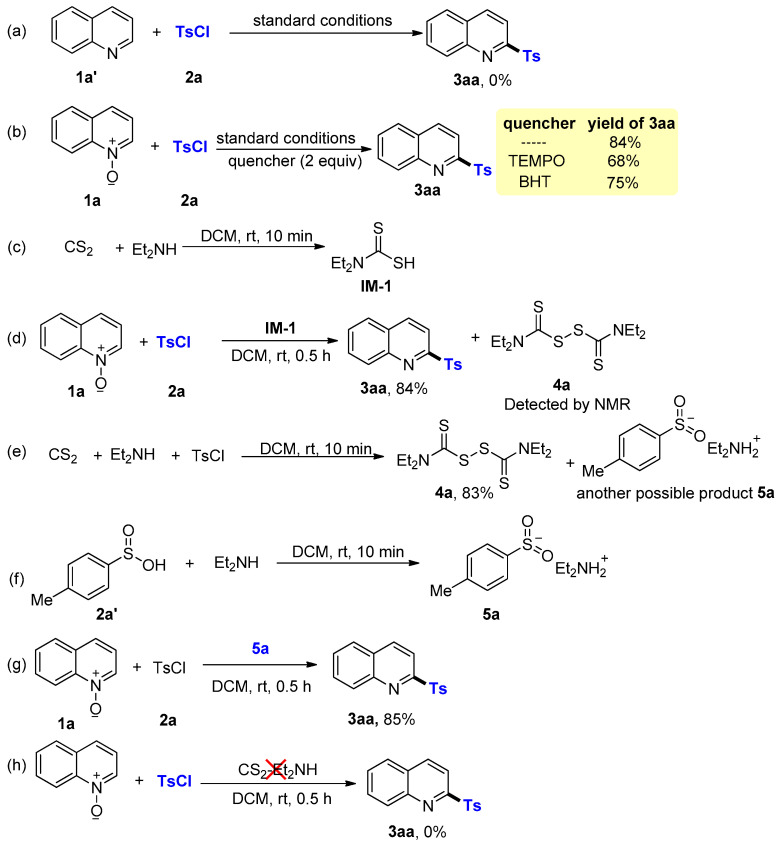
Control experiments.

To investigate the underlying reaction mechanism, a series of control experiments were conducted, as illustrated in Scheme 5. Initially, the reaction of quinoline **1a’** and **2a** under standard conditions failed to produce **3aa**, highlighting the necessity of nitrogen–oxygen groups for the reaction (Scheme 5a). Subsequently, the model reaction was carried out under standard conditions with the addition of TEMPO or BHT as the free radical inhibitors (Scheme 5b). The results indicated that the yields of product **3aa** did not exhibit significant decreases, suggesting that the reaction may not proceed through a free radical pathway. Furthermore, the intermediate **IM-1** was synthesized by reacting CS_2_ with diethylamine, followed by the addition of quinoline *N*-oxide **1a** and *p*-toluenesulfonyl chloride **2a**, resulting in a yield of 84% for **3aa**. Meanwhile, compound **4a** was also isolated and characterized by NMR (Scheme 5c,d). To investigate whether the formation of **4a** necessitates the involvement of quinoline *N*-oxide, a three-component reaction involving CS_2_, diethylamine, and *p*-toluenesulfonyl chloride **2a** was conducted, giving **4a** in 83% yield at room temperature in DCM. It is hypothesized that key intermediate **5a** may also be generated during the reaction (Scheme 5e). Next, compound **5a** was synthesized using *p*-toluenesulfinic acid and diethylamine, and upon its reaction with quinoline *N*-oxide in the presence of *p*-toluenesulfonyl chloride, product **3aa** was obtained in 85% yield (Scheme 5f,g), indicating that the in situ generated **5a** could serve as the sulfonyl source in the current reaction. Finally, the model reaction was carried out in the absence of CS_2_ and Et_2_NH (Scheme 5h), and the results showed that **3aa** was not observed, which further demonstrated the necessity of CS_2_-Et_2_NH in the present reaction.

**Scheme 6:**
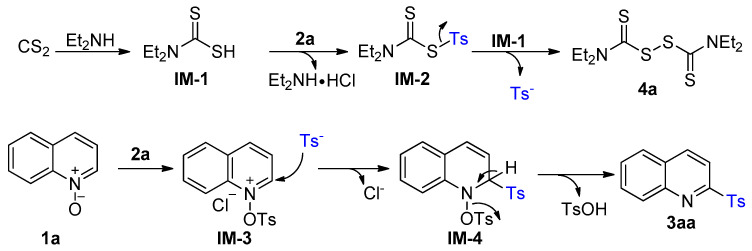
Possible mechanism.

Based on the above-mentioned control experiments and relevant reports in the literature [44,59], a possible reaction pathway was speculated, as shown in Scheme 6. First, a reaction between CS_2_ and Et_2_NH occurred and gave an intermediate **IM-1**, which further reacted with **2a** to give **IM-2**. In the presence of **IM-1**, intermediate **IM-2** was transformed to **4a** with the release of a sulfonyl anion (Ts^−^). Meanwhile, quinoline *N*-oxide **1a** was activated by compound **2a** to produce intermediate **IM-3**. Then, the resulting sulfonyl anion (Ts^−^) attacked IM-3 at the C2 position, providing intermediate **IM-4**, which underwent an elimination process to afford the desired **3aa**.

## 3. Experimental Section

### 3.1. General Information

Unless otherwise noted, all solvents and reagents in this study were commercial and used without further purification. ^1^H, ^13^C and ^19^F NMR spectra were recorded at 400, 100 and 376 MHz, respectively (see Supplementary Materials). Chemical shifts were quoted in ppm relative to CDCl_3_ (δ_H_ = 7.26, δ_C_ = 77.0 ppm). The data are reported as follows: s = singlet, d = doublet, t = triplet, q = quartet, m = multiplet, dd = doublet of doublet, etc. The reactions were monitored by thin-layer chromatography (TLC) using GF254 silica gel-coated TLC plates. Mass spectra were performed on a spectrometer operating on ESI-TOF. Melting points were measured on a melting point apparatus and were uncorrected.

### 3.2. General Procedure for the Preparation of 2-Sulfonylquinolines ***3***

To a round-bottom flask were consecutively added quinoline *N*-oxide **1** (0.3 mmol), CS_2_ (0.45 mmol), diethylamine (0.6 mmol) and sulfonyl chloride **2** (0.6 mmol) in CH_2_Cl_2_ (3 mL). The reaction mixture was stirred at room temperature for about 15–30 min, which was monitored by TLC. Upon completion, CH_2_Cl_2_ (10 mL) and water (10 mL) were added to the mixture, the organic layer was separated and the aqueous layer was further extracted with CH_2_Cl_2_ (2 × 10 mL). The organic phases were combined and dried with anhydrous Na_2_SO_4_, followed by filtration and concentration under vacuo. The residue was purified by a flash chromatography column over silica gel to produce the desired product **3**.

### 3.3. Gram-Scale Synthesis of ***3aa***

To a round-bottom flask were consecutively added quinoline *N*-oxides **1** (5 mmol, 0.7253 g), CS_2_ (7.5 mmol, 0.5696 g), diethylamine (10 mmol, 0.7309 g) and TsCl (10 mmol, 1.8999 g) in CH_2_Cl_2_ (50 mL). The reaction mixture was stirred at room temperature for about 0.5 h. Upon completion, water (30 mL) was added to quench the reaction. The organic layer was separated, and the aqueous layer was further extracted with CH_2_Cl_2_ (2 × 20 mL). The organic phases were combined and dried with anhydrous Na_2_SO_4_, followed by filtration and concentration under vacuo. The residue was purified by a flash chromatography column over silica gel to produce 1.1464 g of **3aa**, yield: 81%.

### 3.4. Characterization Data of Products ***3aa**–**3ta***, ***3xa*** and ***3ab**–**3ap***

*2-tosylquinoline* (**3aa**): White solid (71.3 mg, 84%), m.p.: 141–142 °C. ^1^H NMR (400 MHz, Chloroform-*d*) δ 8.36 (d, *J* = 8.5 Hz, 1H), 8.18 (t, *J* = 8.6 Hz, 2H), 8.02 (d, *J* = 8.1 Hz, 2H), 7.86 (d, *J* = 8.2 Hz, 1H), 7.77 (t, *J* = 7.6 Hz, 1H), 7.64 (t, *J* = 7.5 Hz, 1H), 7.32 (d, *J* = 8.0 Hz, 2H), 2.39 (s, 3H); ^13^C NMR (100 MHz, Chloroform-*d*) δ 158.3, 147.4, 144.8, 138.6, 136.1, 130.9, 130.4, 129.7, 129.1, 129.0, 128.8, 127.6, 117.6, 21.6. NMR data matched previously reported values [20].

*3-methyl-2-tosylquinoline* (**3ba**): White solid (64.2 mg, 72%), m.p.: 114–115 °C. ^1^H NMR (400 MHz, Chloroform-*d*) δ 8.05 (s, 1H), 7.93 (t, *J* = 7.7 Hz, 3H), 7.76 (d, *J* = 8.1 Hz, 1H), 7.65 (t, *J* = 7.5 Hz, 1H), 7.58 (t, *J* = 7.4 Hz, 1H), 7.36 (d, *J* = 7.9 Hz, 2H), 2.86 (s, 3H), 2.46 (s, 3H); ^13^C NMR (100 MHz, Chloroform-*d*) δ 157.0, 144.7, 144.5, 139.8, 135.8, 129.9, 129.7, 129.4, 129.3, 129.1, 128.9, 128.5, 126.6, 21.7, 18.8. NMR data matched previously reported values [22].

*3-chloro-2-tosylquinoline* (**3ca**): White solid (67.5 mg, 71%), m.p.: 108–109 °C. ^1^H NMR (400 MHz, Chloroform-*d*) δ 8.28 (s, 1H), 8.06 (d, *J* = 8.5 Hz, 1H), 7.96 (d, *J* = 7.8 Hz, 2H), 7.76 (q, *J* = 8.3 Hz, 2H), 7.71–7.64 (m, 1H), 7.36 (d, *J* = 7.9 Hz, 2H), 2.46 (s, 3H); ^13^C NMR (100 MHz, Chloroform-*d*) δ 153.6, 145.0, 144.3, 139.0, 135.2, 130.9, 130.3, 130.1, 129.6, 129.5, 129.4, 126.5, 124.7, 21.7; HRMS (ESI): *m*/*z* [M + H]^+^ calcd. for C_16_H_13_ClNO_2_S: 318.0350; found: 318.0353.

*4-methyl-2-tosylquinoline* (**3da**): White solid (58.8 mg, 66%), m.p.: 144–145 °C. ^1^H NMR (400 MHz, Chloroform-*d*) δ 8.15 (d, *J* = 8.4 Hz, 1H), 8.04–7.96 (m, 4H), 7.74 (t, *J* = 7.6 Hz, 1H), 7.64 (t, *J* = 7.4 Hz, 1H), 7.31 (d, *J* = 7.3 Hz, 2H), 2.77 (s, 3H), 2.38 (s, 3H); ^13^C NMR (100 MHz, Chloroform-*d*) δ 157.9, 147.8, 147.2, 144.6, 136.3, 131.0, 130.4, 129.7, 129.0, 128.8, 128.7, 123.7, 118.0, 21.6, 19.1. NMR data matched previously reported values [26].

*4-chloro-2-tosylquinoline* (**3ea**): White solid (65.6 mg, 69%), m.p.: 168–169 °C. ^1^H NMR (400 MHz, Chloroform-*d*) δ 8.26 (s, 1H), 8.22 (d, *J* = 8.4 Hz, 1H), 8.17 (d, *J* = 8.5 Hz, 1H), 8.01 (d, *J* = 8.1 Hz, 2H), 7.81 (t, *J* = 7.2 Hz, 1H), 7.73 (t, *J* = 7.6 Hz, 1H), 7.33 (d, *J* = 7.9 Hz, 2H), 2.39 (s, 3H); ^13^C NMR (100 MHz, Chloroform-*d*) δ 158.1, 148.0, 145.1, 135.5, 131.7, 130.7, 130.1, 129.8, 129.1, 126.9, 124.1, 117.8, 21.6. NMR data matched previously reported values [13].

*6-isopropyl-2-tosylquinoline* (**3fa**): White solid (71.2 mg, 73%), m.p.: 147–148 °C. ^1^H NMR (400 MHz, Chloroform-*d*) δ 8.28 (d, *J* = 8.4 Hz, 1H), 8.15 (d, *J* = 8.6 Hz, 1H), 8.10 (d, *J* = 8.7 Hz, 1H), 8.00 (d, *J* = 7.9 Hz, 2H), 7.71–7.60 (m, 2H), 7.30 (d, *J* = 7.7 Hz, 2H), 3.15–3.04 (m, 1H), 2.38 (s, 3H), 1.32 (d, *J* = 6.8 Hz, 6H); ^13^C NMR (100 MHz, Chloroform-*d*) δ 157.4, 150.2, 146.4, 144.6, 138.1, 136.4, 131.0, 130.2, 129.7, 128.9, 128.9, 123.6, 117.7, 34.2, 23.6, 21.6. NMR data matched previously reported values [26].

*6-methoxy-2-tosylquinoline* (**3ga**): White solid (71.4 mg, 76%), m.p.: 162–163 °C. ^1^H NMR (400 MHz, Chloroform-*d*) δ 8.20 (d, *J* = 8.6 Hz, 1H), 8.12 (d, *J* = 8.6 Hz, 1H), 8.03 (d, *J* = 9.3 Hz, 1H), 7.99 (d, *J* = 8.0 Hz, 2H), 7.39 (dd, *J* = 9.3, 2.6 Hz, 1H), 7.30 (d, *J* = 8.0 Hz, 2H), 7.07 (d, *J* = 2.4 Hz, 1H), 3.92 (s, 3H), 2.38 (s, 3H); ^13^C NMR (100 MHz, Chloroform-*d*) δ 159.7, 155.6, 144.5, 143.6, 136.8, 136.5, 131.7, 130.3, 129.7, 128.8, 124.2, 118.1, 104.6, 55.7, 21.6. NMR data matched previously reported values [19].

*6-fluoro-2-tosylquinoline* (**3ha**): White solid (69.5 mg, 77%), m.p.: 123–124 °C. ^1^H NMR (400 MHz, Chloroform-*d*) δ 8.32 (d, *J* = 8.6 Hz, 1H), 8.21 (d, *J* = 8.7 Hz, 1H), 8.20–8.13 (m, 1H), 8.01 (d, *J* = 8.0 Hz, 2H), 7.55 (t, *J* = 8.7 Hz, 1H), 7.48 (d, *J* = 8.5 Hz, 1H), 7.33 (d, *J* = 8.0 Hz, 2H), 2.41 (s, 3H); ^13^C NMR (100 MHz, Chloroform-*d*) δ 161.9 (d, *J*_C-F_ = 251.6 Hz), 157.9, 144.9, 144.5, 137.9 (d, *J*_C-F_ = 5.7 Hz), 135.9, 133.1 (d, *J*_C-F_ = 9.5 Hz), 129.8, 129.7, 129.0, 121.5 (d, *J*_C-F_ = 26.0 Hz), 118.5, 110.7 (d, *J*_C-F_ = 21.9 Hz), 21.6; ^19^F NMR (376 MHz, Chloroform-*d*) δ −108.35. NMR data matched previously reported values [19].

*6-chloro-2-tosylquinoline* (**3ia**): White solid (75.1 mg, 79%), m.p.: 165–166 °C. ^1^H NMR (400 MHz, Chloroform-*d*) δ 8.28 (d, *J* = 8.6 Hz, 1H), 8.21 (d, *J* = 8.4 Hz, 1H), 8.10 (d, *J* = 8.9 Hz, 1H), 8.00 (d, *J* = 7.9 Hz, 2H), 7.88–7.83 (m, 1H), 7.70 (d, *J* = 9.1 Hz, 1H), 7.33 (d, *J* = 7.8 Hz, 2H), 2.40 (s, 3H); ^13^C NMR (100 MHz, Chloroform-*d*) δ 158.7, 145.8, 145.0, 137.7, 135.8, 135.2, 132.0, 131.9, 129.8, 129.3, 129.1, 126.3, 118.6, 21.6. NMR data matched previously reported values [18].

*6-bromo-2-tosylquinoline* (**3ja**): White solid (83.4 mg, 77%), m.p.: 179–180 °C. ^1^H NMR (400 MHz, Chloroform-*d*) δ 8.27 (d, *J* = 8.5 Hz, 1H), 8.20 (d, *J* = 8.6 Hz, 1H), 8.05–7.98 (m, 4H), 7.83 (dd, *J* = 9.0, 2.2 Hz, 1H), 7.33 (d, *J* = 8.0 Hz, 2H), 2.40(s, 3H); ^13^C NMR (100 MHz, Chloroform-*d*) δ 158.7, 145.9, 145.0, 137.6, 135.7, 134.5, 131.9, 129.8, 129.7, 129.1, 123.5, 118.6, 21.7. NMR data matched previously reported values [18].

*7-methyl-2-tosylquinoline* (**3ka**): White solid (58.8 mg, 66%), m.p.: 209–210 °C. ^1^H NMR (400 MHz, Chloroform-*d*) δ 8.30 (d, *J* = 8.5 Hz, 1H), 8.12 (d, *J* = 8.5 Hz, 1H), 8.01 (d, *J* = 8.0 Hz, 2H), 7.94 (s, 1H), 7.75 (d, *J* = 8.4 Hz, 1H), 7.47 (d, *J* = 8.4 Hz, 1H), 7.32 (d, *J* = 8.0 Hz, 2H), 2.54 (s, 3H), 2.39 (s, 3H); ^13^C NMR (100 MHz, Chloroform-*d*) δ 158.2, 147.7, 144.6, 141.6, 138.2, 136.2, 131.4, 129.7, 129.2, 129.0, 127.2, 126.9, 116.8, 21.8, 21.6. NMR data matched previously reported values [23].

*7-chloro-2-tosylquinoline* (**3la**): White solid (60.9 mg, 64%), m.p.: 173–174 °C. ^1^H NMR (400 MHz, Chloroform-*d*) δ 8.35 (d, *J* = 8.5 Hz, 1H), 8.20 (d, *J* = 8.6 Hz, 1H), 8.16 (s, 1H), 8.00 (d, *J* = 8.0 Hz, 2H), 7.81 (d, *J* = 8.8 Hz, 1H), 7.60 (d, *J* = 8.7 Hz, 1H), 7.34 (d, *J* = 7.9 Hz, 2H), 2.41 (s, 3H); ^13^C NMR (100 MHz, Chloroform-*d*) δ 159.5, 147.7, 145.0, 138.6, 137.1, 135.7, 130.2, 129.8, 129.2, 128.8, 128.0, 127.1, 117.7, 21.7. NMR data matched previously reported values [60].

*7-bromo-2-tosylquinoline* (**3ma**): White solid (72.6 mg, 67%), m.p.: 186–187 °C. ^1^H NMR (400 MHz, Chloroform-*d*) δ 8.39–8.32 (m, 2H), 8.22 (d, *J* = 8.6 Hz, 1H), 8.00 (d, *J* = 7.9 Hz, 2H), 7.74 (s, 2H), 7.34 (d, *J* = 7.9 Hz, 2H), 2.42 (s, 3H); ^13^C NMR (100 MHz, Chloroform-*d*) δ 159.4, 147.8, 145.0, 138.7, 135.6, 132.7, 132.6, 129.8, 129.2, 128.8, 127.3, 125.3, 117.9, 21.7; HRMS (ESI): *m*/*z* [M + H]^+^ calcd. for C_16_H_13_BrNO_2_S: 361.9845; found: 361.9849.

*2-tosyl-7-(trifluoromethyl)quinoline* (**3na**): White solid (76.9 mg, 73%), m.p.: 162–163 °C. ^1^H NMR (400 MHz, Chloroform-*d*) δ 8.45 (d, *J* = 9.1 Hz, 2H), 8.32 (d, *J* = 8.5 Hz, 1H), 8.04–7.99 (m, 3H), 7.82 (d, *J* = 8.4 Hz, 1H), 7.35 (d, *J* = 8.0 Hz, 2H), 2.42 (s, 3H); ^13^C NMR (100 MHz, Chloroform-*d*) δ 160.0, 146.3, 145.2, 138.8, 135.4, 132.7 (q, *J*_C-F_ = 33.0 Hz), 130.0, 129.9, 129.2, 129.0, 128.2 (q, *J*_C-F_ = 4.3 Hz), 124.6 (q, *J*_C-F_ = 2.8 Hz), 123.4 (q, *J*_C-F_ = 271.1 Hz), 119.4, 21.7. NMR data matched previously reported values [60].

*8-methyl-2-tosylquinoline* (**3oa**): White solid (65.9 mg, 74%), m.p.: 125–126 °C. ^1^H NMR (400 MHz, Chloroform-*d*) δ 8.32 (d, *J* = 8.5 Hz, 1H), 8.19 (d, *J* = 8.5 Hz, 1H), 8.06 (d, *J* = 7.9 Hz, 2H), 7.68 (d, *J* = 8.1 Hz, 1H), 7.59 (d, *J* = 6.8 Hz, 1H), 7.51 (t, *J* = 7.5 Hz, 1H), 7.34 (d, *J* = 7.9 Hz, 2H), 2.68 (s, 3H), 2.41 (s, 3H); ^13^C NMR (100 MHz, Chloroform-*d*) δ 157.3, 146.4, 144.6, 138.7, 138.4, 135.9, 130.8, 129.5, 129.3, 128.9, 128.8, 125.5, 116.7, 21.6, 17.5. NMR data matched previously reported values [22].

*8-(4-bromophenyl)-2-tosylquinoline* (**3pa**): White solid (85.2 mg, 65%), m.p.: 137–138 °C. ^1^H NMR (400 MHz, Chloroform-*d*) δ 8.43 (dd, *J* = 8.5, 2.9 Hz, 1H), 8.25 (dd, *J* = 8.5, 2.9 Hz, 1H), 7.90–7.79 (m, 3H), 7.79–7.72 (m, 1H), 7.75–7.64 (m, 1H), 7.47 (dd, *J* = 8.1, 2.8 Hz, 2H), 7.31–7.20 (m, 4H), 2.46 (s, 3H); ^13^C NMR (100 MHz, Chloroform-*d*) δ 158.6, 144.8, 144.3, 140.0, 139.0, 136.8, 134.9, 132.2, 131.1, 130.8, 129.7, 129.4, 129.2, 128.8, 127.6, 121.7, 116.6, 21.7; HRMS (ESI): *m*/*z* [M + H]^+^ calcd. for C_22_H_17_BrNO_2_S: 438.0158; found: 438.0155.

*4-chloro-6,7-dimethoxy-2-tosylquinoline* (**3qa**): White solid (70.1 mg, 62%), m.p.: 196–197 °C. ^1^H NMR (400 MHz, Chloroform-*d*) δ 8.13 (s, 1H), 7.99 (d, *J* = 8.1 Hz, 2H), 7.48 (s, 1H), 7.38 (s, 1H), 7.33 (d, *J* = 8.0 Hz, 2H), 4.06 (s, 3H), 4.02 (s, 3H), 2.40 (s, 3H); ^13^C NMR (100 MHz, Chloroform-*d*) δ 155.6, 154.2, 152.8, 145.6, 144.8, 142.0, 136.3, 129.8, 128.9, 123.4, 116.7, 108.9, 101.3, 56.5, 56.4, 21.7; HRMS (ESI): *m*/*z* [M + H]^+^ calcd. for C_18_H_17_ClNO_4_S: 378.0561; found: 378.0566.

*4-phenyl-2-tosylpyridine* (**3xa**): White solid (47.3 mg, 51%), m.p.: 149–150 °C. ^1^H NMR (400 MHz, Chloroform-*d*) δ 8.68 (s, 1H), 8.41 (s, 1H), 7.98 (d, *J* = 7.0 Hz, 2H), 7.65 (d, *J* = 14.2 Hz, 3H), 7.55–7.41 (m, 3H), 7.34 (d, *J* = 7.1 Hz, 2H), 2.41 (s, 3H); ^13^C NMR (100 MHz, Chloroform-*d*) δ 159.7, 150.8, 144.8, 136.5, 135.9, 130.0, 129.8, 129.4, 129.0, 127.1, 124.3, 119.8, 21.6. NMR data matched previously reported values [22].

*2-(phenylsulfonyl)quinoline* (**3ab**): White solid (57.3 mg, 71%), m.p.: 159–160 °C. ^1^H NMR (400 MHz, Chloroform-*d*) δ 8.37 (d, *J* = 8.5 Hz, 1H), 8.21 (d, *J* = 8.5 Hz, 1H), 8.19–8.12 (m, 3H), 7.87 (d, *J* = 8.1 Hz, 1H), 7.78 (t, *J* = 7.5 Hz, 1H), 7.66 (d, *J* = 7.7 Hz, 1H), 7.59 (d, *J* = 7.2 Hz, 1H), 7.53 (t, *J* = 7.5 Hz, 2H); ^13^C NMR (100 MHz, Chloroform-*d*) δ 158.0, 147.4, 139.0, 138.7, 133.7, 131.0, 130.3, 129.2, 129.0, 128.9, 128.8, 127.7, 117.7. NMR data matched previously reported values [16].

*2-((4-methoxyphenyl)sulfonyl)quinoline* (**3ac**): White solid (65.5 mg, 73%), m.p.: 131–132 °C. ^1^H NMR (400 MHz, Chloroform-*d*) δ 8.36 (d, *J* = 8.5 Hz, 1H), 8.21–8.13 (m, 2H), 8.07 (d, *J* = 8.7 Hz, 2H), 7.86 (d, *J* = 8.0 Hz, 1H), 7.78 (t, *J* = 7.7 Hz, 1H), 7.65 (t, *J* = 7.5 Hz, 1H), 6.99 (d, *J* = 8.7 Hz, 2H), 3.84 (s, 3H); ^13^C NMR (100 MHz, Chloroform-*d*) δ 163.9, 158.6, 147.4, 138.6, 133.8, 131.3, 130.9, 130.5, 129.0, 128.7, 127.6, 117.5, 114.4, 55.6. NMR data matched previously reported values [19].

*2-((4-fluorophenyl)sulfonyl)quinoline* (**3ad**): White solid (71.5 mg, 83%), m.p.: 124–125 °C. ^1^H NMR (400 MHz, Chloroform-*d*) δ 8.39 (d, *J* = 8.5 Hz, 1H), 8.24–8.11 (m, 4H), 7.88 (d, *J* = 8.2 Hz, 1H), 7.79 (t, *J* = 7.6 Hz, 1H), 7.66 (t, *J* = 7.5 Hz, 1H), 7.21 (t, *J* = 8.5 Hz, 2H); ^13^C NMR (100 MHz, Chloroform-*d*) δ 165.9 (d, *J*_C-F_ = 254.9 Hz), 157.9, 147.4, 138.8, 134.9 (d, *J*_C-F_ = 3.1 Hz), 132.0 (d, *J*_C-F_ = 9.6 Hz), 131.1, 130.3, 129.3, 128.8, 127.7, 117.4, 116.4 (d, *J*_C-F_ = 22.5 Hz); ^19^F NMR (376 MHz, Chloroform-*d*) δ −103.39. NMR data matched previously reported values [18].

*2-((4-chlorophenyl)sulfonyl)quinoline* (**3ae**): White solid (74.5 mg, 82%), m.p.: 190–191 °C. ^1^H NMR (400 MHz, Chloroform-*d*) δ 8.40 (dd, *J* = 8.4, 2.3 Hz, 1H), 8.20 (dd, *J* = 8.4, 2.4 Hz, 1H), 8.15 (d, *J* = 8.1 Hz, 1H), 8.08 (dd, *J* = 8.2, 2.4 Hz, 2H), 7.89 (d, *J* = 7.2 Hz, 1H), 7.80 (t, *J* = 8.3 Hz, 1H), 7.68 (t, *J* = 7.0 Hz, 1H), 7.51 (dd, *J* = 8.2, 2.4 Hz, 2H); ^13^C NMR (100 MHz, Chloroform-*d*) δ 157.7, 147.4, 140.5, 138.8, 137.5, 131.1, 130.5, 130.3, 129.4, 129.3, 128.9, 127.7, 117.5. NMR data matched previously reported values [20].

*2-((4-bromophenyl)sulfonyl)quinoline* (**3af**): White solid (81.2 mg, 78%), m.p.: 146–147 °C. ^1^H NMR (400 MHz, Chloroform-*d*) δ 8.39 (d, *J* = 8.5 Hz, 1H), 8.20 (d, *J* = 8.5 Hz, 1H), 8.15 (d, *J* = 8.5 Hz, 1H), 8.00 (d, *J* = 8.4 Hz, 2H), 7.88 (d, *J* = 8.2 Hz, 1H), 7.80 (t, *J* = 7.7 Hz, 1H), 7.70–7.64 (m, 3H); ^13^C NMR (100 MHz, Chloroform-*d*) δ 157.7, 147.4, 138.8, 138.0, 132.4, 131.1, 130.6, 130.3, 129.3, 129.2, 128.8, 127.7, 117.5. NMR data matched previously reported values [20].

*1-(4-(quinolin-2-ylsulfonyl)phenyl)ethanone* (**3ag**): White solid (78.4 mg, 84%), m.p.: 153–154 °C. ^1^H NMR (400 MHz, Chloroform-*d*) δ 8.40 (d, *J* = 8.5 Hz, 1H), 8.26–8.20 (m, 3H), 8.13 (d, *J* = 8.6 Hz, 1H), 8.07 (d, *J* = 8.2 Hz, 2H), 7.88 (d, *J* = 8.2 Hz, 1H), 7.79 (t, *J* = 7.5 Hz, 1H), 7.67 (t, *J* = 7.5 Hz, 1H), 2.62 (s, 3H); ^13^C NMR (100 MHz, Chloroform-*d*) δ 196.8, 157.4, 147.4, 145.0, 142.8, 140.7, 138.9, 131.2, 130.3, 129.4, 128.9, 128.7, 127.7, 117.6, 26.9. NMR data matched previously reported values [26].

*2-((4-(trifluoromethoxy)phenyl)sulfonyl)quinoline* (**3ah**): White solid (66.7 mg, 63%), m.p.: 162–163 °C. ^1^H NMR (400 MHz, Chloroform-*d*) δ 8.40 (d, *J* = 8.5 Hz, 1H), 8.31–8.18 (m, 3H), 8.15 (d, *J* = 8.5 Hz, 1H), 7.89 (d, *J* = 8.2 Hz, 1H), 7.80 (t, *J* = 7.7 Hz, 1H), 7.67 (t, *J* = 7.5 Hz, 1H), 7.35 (d, *J* = 8.4 Hz, 2H); ^13^C NMR (100 MHz, Chloroform-*d*) δ 157.6, 153.0, 147.4, 138.9, 137.2, 131.4, 131.1, 130.3, 129.4, 128.9, 127.7, 122.8 (q, *J*_C-F_ = 273.1 Hz), 120.7, 117.5; ^19^F NMR (376 MHz, Chloroform-*d*) δ -57.64. NMR data matched previously reported values [28].

*2-(m-tolylsulfonyl)quinoline* (**3ai**): White solid (68.8 mg, 81%), m.p.: 124–125 °C. ^1^H NMR (400 MHz, Chloroform-*d*) δ 8.37 (d, *J* = 8.4 Hz, 1H), 8.19 (t, *J* = 7.7 Hz, 2H), 7.96–7.90 (m, 2H), 7.87 (d, *J* = 8.1 Hz, 1H), 7.78 (t, *J* = 7.7 Hz, 1H), 7.65 (t, *J* = 7.1 Hz, 1H), 7.43–7.35 (m, 2H), 2.41 (s, 3H); ^13^C NMR (100 MHz, Chloroform-*d*) δ 158.1, 147.4, 139.3, 138.9, 138.7, 134.5, 130.9, 130.4, 129.2, 129.2, 128.9, 128.8, 127.7, 126.1, 117.8, 21.3. NMR data matched previously reported values [13].

*2-((3-bromophenyl)sulfonyl)quinoline* (**3aj**): White solid (79.1 mg, 76%), m.p.: 115–116 °C. ^1^H NMR (400 MHz, Chloroform-*d*) δ 8.40 (d, *J* = 8.5 Hz, 1H), 8.27 (s, 1H), 8.20 (d, *J* = 8.5 Hz, 1H), 8.16 (d, *J* = 8.6 Hz, 1H), 8.08 (d, *J* = 7.9 Hz, 1H), 7.89 (d, *J* = 8.2 Hz, 1H), 7.80 (t, *J* = 7.7 Hz, 1H), 7.69 (dt, *J* = 15.3, 7.8 Hz, 2H), 7.41 (t, *J* = 7.9 Hz, 1H); ^13^C NMR (100 MHz, Chloroform-*d*) δ 157.4, 147.4, 140.9, 138.9, 136.7, 131.8, 131.1, 130.5, 130.3, 129.4, 128.9, 127.7, 127.6, 123.0, 117.6. NMR data matched previously reported values [18].

*2-((3-chloro-4-fluorophenyl)sulfonyl)quinoline* (**3ak**): White solid (76.1 mg, 79%), m.p.: 128–129 °C. ^1^H NMR (400 MHz, Chloroform-*d*) δ 8.42 (d, *J* = 8.5 Hz, 1H), 8.21 (t, *J* = 7.7 Hz, 2H), 8.15 (d, *J* = 8.6 Hz, 1H), 8.09–8.03 (m, 1H), 7.90 (d, *J* = 8.2 Hz, 1H), 7.81 (t, *J* = 7.6 Hz, 1H), 7.69 (t, *J* = 7.5 Hz, 1H), 7.30 (t, *J* = 8.5 Hz, 1H); ^13^C NMR (100 MHz, Chloroform-*d*) δ 161.4 (d, *J*_C-F_ = 257.0 Hz), 157.4, 147.4, 139.0, 136.0 (d, *J*_C-F_ = 3.9 Hz), 132.0 (d, *J*_C-F_ = 1.1 Hz), 131.2, 130.3, 129.8 (d, *J*_C-F_ = 8.8 Hz), 129.5, 128.9, 127.7, 122.5 (d, *J*_C-F_ = 18.6 Hz), 117.4 (d, *J*_C-F_ = 22.3 Hz), 117.4; ^19^F NMR (376 MHz, Chloroform-*d*) δ −105.56. NMR data matched previously reported values [26].

*2-((3,4-difluorophenyl)sulfonyl)quinoline* (**3al**): White solid (67.7 mg, 74%), m.p.: 126–127 °C. ^1^H NMR (400 MHz, Chloroform-*d*) δ 8.41 (d, *J* = 8.5 Hz, 1H), 8.20 (d, *J* = 8.5 Hz, 1H), 8.15 (d, *J* = 8.6 Hz, 1H), 8.00 (t, *J* = 8.2 Hz, 1H), 7.92 (dd, *J* = 14.8, 8.5 Hz, 2H), 7.81 (t, *J* = 7.7 Hz, 1H), 7.68 (t, *J* = 7.5 Hz, 1H), 7.33 (q, *J* = 8.7 Hz, 1H); ^13^C NMR (100 MHz, Chloroform-*d*) δ 157.4, 153.9 (dd, *J*_C-F_ = 257.2 Hz, 12.5 Hz), 150.1 (dd, *J*_C-F_ = 253.5 Hz, 12.5 Hz), 147.4, 139.0, 135.7 (dd, *J*_C-F_ = 4.8 Hz, 3.7 Hz), 131.2, 130.3, 129.5, 128.9, 127.7, 126.4 (dd, *J*_C-F_ = 7.8 Hz, 4.0 Hz), 119.1 (dd, *J*_C-F_ = 19.8 Hz, 1.9 Hz), 118.2 (d, *J*_C-F_ = 18.4 Hz), 117.4. NMR data matched previously reported values [26].

*2-(o-tolylsulfonyl)quinoline* (**3am**): White solid (61.1 mg, 72%), m.p.: 115–116 °C. ^1^H NMR (400 MHz, Chloroform-*d*) δ 8.41 (d, *J* = 8.5 Hz, 1H), 8.33 (d, *J* = 7.8 Hz, 1H), 8.19 (d, *J* = 8.6 Hz, 1H), 8.13 (d, *J* = 8.6 Hz, 1H), 7.91 (d, *J* = 8.2 Hz, 1H), 7.78 (d, *J* = 8.2 Hz, 1H), 7.68 (t, *J* = 7.5 Hz, 1H), 7.52 (t, *J* = 7.4 Hz, 1H), 7.44 (t, *J* = 7.6 Hz, 1H), 7.28 (s, 1H), 2.58 (s, 3H); ^13^C NMR (100 MHz, Chloroform-*d*) δ 158.1, 147.1, 139.1, 138.6, 137.1, 133.9, 132.4, 130.9, 130.6, 130.3, 129.1, 128.8, 127.7, 126.3, 117.7, 20.7. NMR data matched previously reported values [20].

*2-((2-chlorophenyl)sulfonyl)quinoline* (**3an**): White solid (61.8 mg, 68%), m.p.: 166–167 °C. ^1^H NMR (400 MHz, Chloroform-*d*) δ 8.54–8.46 (m, 1H), 8.43 (d, *J* = 8.5 Hz, 1H), 8.32 (d, *J* = 8.6 Hz, 1H), 8.04 (d, *J* = 8.5 Hz, 1H), 7.91 (d, *J* = 8.1 Hz, 1H), 7.75 (t, *J* = 7.5 Hz, 1H), 7.66 (t, *J* = 7.4 Hz, 1H), 7.60–7.52 (m, 2H), 7.46–7.37 (m, 1H); ^13^C NMR (100 MHz, Chloroform-*d*) δ 157.3, 147.1, 138.3, 136.7, 134.9, 133.0, 132.1, 131.5, 130.9, 130.2, 129.2, 129.0, 127.8, 127.1, 118.3. NMR data matched previously reported values [19].

*2-(thiophen-2-ylsulfonyl)quinoline* (**3ao**)*:* White solid (51.2 mg, 62%), m.p.: 145–146 °C. ^1^H NMR (400 MHz, Chloroform-*d*) δ 8.39 (d, *J* = 8.5 Hz, 1H), 8.20 (d, *J* = 8.4 Hz, 2H), 7.92–7.86 (m, 2H), 7.83–7.77 (m, 1H), 7.75–7.70 (m, 1H), 7.69–7.63 (m, 1H), 7.15–7.08 (m, 1H); ^13^C NMR (100 MHz, Chloroform-*d*) δ 158.0, 147.4, 139.7, 138.9, 135.3, 135.3, 131.1, 130.4, 129.3, 128.9, 127.9, 127.7, 117.3. NMR data matched previously reported values [27].

*Diethylcarbamodithioic acid* (**IM-1**): Yellow oil, ^1^H NMR (400 MHz, Chloroform-*d*) δ 8.07 (s, 1H), 3.99 (s, 2H), 3.09 (q, *J* = 7.3 Hz, 2H), 1.39–1.26 (m, 3H), 1.16 (t, *J* = 7.0 Hz, 3H); ^13^C NMR (100 MHz, Chloroform-*d*) δ 208.1, 47.6, 40.8, 12.1, 10.6.

*Tetraethylthiuram Disulfide* (**4a**): White solid; ^1^H NMR (400 MHz, Chloroform-*d*) δ 4.17–3.76 (m, 8H), 1.55–1.37 (m, 6H), 1.34–1.20 (m, 6H); ^13^C NMR (100 MHz, Chloroform-*d*) δ 192.5, 51.9, 47.5, 13.3, 11.3.

*Diethylamine 4-methylbenzenesulfinate* (**5a**): Yellow oil; ^1^H NMR (400 MHz, Chloroform-*d*) δ 9.73 (s, 2H), 7.48 (d, *J* = 7.8 Hz, 2H), 7.14 (d, *J* = 7.7 Hz, 2H), 2.68 (q, *J* = 7.2 Hz, 4H), 2.29 (s, 3H), 1.18 (t, *J* = 7.3 Hz, 6H); ^13^C NMR (100 MHz, Chloroform-*d*) δ 153.3, 139.0, 128.8, 123.8, 41.4, 21.1, 10.9.

## 4. Conclusions

In summary, we have developed a practical and efficient approach to the synthesis of 2-sulfonylquinolines via deoxygenative C2-H sulfonylation of quinoline *N*-oxides with sulfonyl chlorides in the presence of CS_2_ and diethylamine. The reaction proceeds under mild and transition-metal-free conditions, providing the sulfonylation products bearing various functional groups in satisfactory yields. In this reaction, sulfonyl chlorides act as both electrophilic reagents for activating quinoline *N*-oxides and readily available sulfonylating reagents. The present method provides a novel strategy for generating nucleophilic sulfonyl sources using electrophilic sulfonyl chlorides as starting materials. Further sulfonyl chlorides-promoted sulfonylation reactions and their potential applications are currently underway in our laboratory.

## Figures and Tables

**Table 1:** Optimization of reaction conditions ^a^.

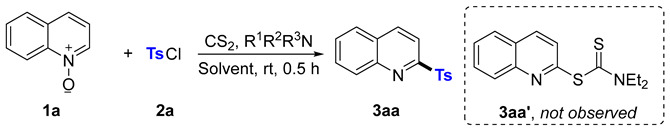				
Entry	R^1^R^2^R^3^N	Solvent	Ratios of 2a to 1a	Yield of 3aa %
1	Et_2_NH	THF	2	55
2	Et_2_NH	DCM	2	67
3	Et_2_NH	DCE	2	63
4	Et_2_NH	EtOAc	2	42
5	Et_2_NH	Acetone	2	0
6	Et_2_NH	CH_3_CN	2	47
7	Et_2_NH	DMF	2	24
8	Me_2_NH	DCM	2	43
9	IPr_2_NH	DCM	2	7
10	morpholine	DCM	2	12
11	piperidine	DCM	2	8
12	Et_2_NH	DCM	2	0
13	Et_2_NH	DCM	2.5	67
14	Et_2_NH	DCM	3	66
15	Et_2_NH	DCM	1.5	53
16 ^b^	Et_2_NH	DCM	2	79
17 ^c^	Et_2_NH	DCM	2	84
18 ^d^	Et_2_NH	DCM	2	82
19 ^e^	Et_2_NH	DCM	2	0
20	none	DCM	2	0

^a^ Conditions: unless otherwise noted, **1a**. (0.3 mmol, 1 eq), CS_2_ (0.36 mmol, 1.2 eq), R^1^R^2^R^3^N (0.45 mmol, 1.5 eq); **2a**. solvent (3 mL), rt, 0.5 h, isolated yields were given. ^b^ Here, 2 equiv. of Et_2_NH was used. ^c^ Here, 1.5 equiv. of CS_2_ was used. ^d^ Here, 2 equiv. of CS_2_ was used. ^e^ The reaction proceeded in the absence of CS_2_.

## Data Availability

The data presented in this study are available on request from the corresponding author.

## Supplementary Materials

The following supporting information can be downloaded at: https://www.mdpi.com/article/10.3390/molecules29122863/s1, Copies of the ^1^H NMR and ^13^C NMR spectra for compounds **3aa**–**3ta, 3xa** and **3ab**–**3ao**.

## References

[1] Andries K., Verhasselt P., Guillemont J., Göhlmann H.W.H., Neefs J.-M., Winkler H., Van Gestel J., Timmerman P., Zhu M., Lee E. (2005). A Diarylquinoline Drug Active on the ATP Synthase of Mycobacterium tuberculosis. Science.

[2] Galambos J., Domány G., Nógrádi K., Wágner G., Keserű G.M., Bobok A., Kolok S., Mikó-Bakk M.L., Vastag M., Sághy K. (2016). 4-Aryl-3-arylsulfonyl-quinolines as negative allosteric modulators of metabotropic GluR5 receptors: From HTS hit to development candidate. Bioorg. Med. Chem. Lett..

[3] Hayat F., Moseley E., Salahuddin A., Van Zyl R.L., Azam A. (2011). Antiprotozoal activity of chloroquinoline based chalcones. Eur. J. Med. Chem..

[4] Michael J.P. (2008). Quinoline, quinazoline and acridone alkaloids. Nat. Prod. Rep..

[5] Strekowski L., Say M., Henary M., Ruiz P., Manzel L., Macfarlane D.E., Bojarski A.J. (2003). Synthesis and Activity of Substituted 2-Phenylquinolin-4-amines, Antagonists of Immunostimulatory CpG-Oligodeoxynucleotides. J. Med. Chem..

[6] Zajdel P., Marciniec K., Maślankiewicz A., Paluchowska M.H., Satała G., Partyka A., Jastrzębska-Więsek M., Wróbel D., Wesołowska A., Duszyńska B. (2011). Arene- and quinoline-sulfonamides as novel 5-HT7 receptor ligands. Biorg. Med. Chem..

[7] Hussain I., Yawer M.A., Lalk M., Lindequist U., Villinger A., Fischer C., Langer P. (2008). Hetero-Diels–Alder reaction of 1,3-bis(trimethylsilyloxy)-1,3-butadienes with arylsulfonylcyanides. Synthesis and antimicrobial activity of 4-hydroxy-2-(arylsulfonyl)pyridines. Biorg. Med. Chem..

[8] Lee H.-Y., Chang C.-Y., Su C.-J., Huang H.-L., Mehndiratta S., Chao Y.-H., Hsu C.-M., Kumar S., Sung T.-Y., Huang Y.-Z. (2016). 2-(Phenylsulfonyl)quinoline *N*-hydroxyacrylamides as potent anticancer agents inhibiting histone deacetylase. Eur. J. Med. Chem..

[9] Lee H.-Y., Chang J.-Y., Nien C.-Y., Kuo C.-C., Shih K.-H., Wu C.-H., Chang C.-Y., Lai W.-Y., Liou J.-P. (2011). 5-Amino-2-aroylquinolines as Highly Potent Tubulin Polymerization Inhibitors. Part 2. The Impact of Bridging Groups at Position C-2. J. Med. Chem..

[10] Cacchi S., Fabrizi G., Goggiamani A., Parisi L.M., Bernini R. (2004). Unsymmetrical Diaryl Sulfones and Aryl Vinyl Sulfones through Palladium-Catalyzed Coupling of Aryl and Vinyl Halides or Triflates with Sulfinic Acid Salts. J. Org. Chem..

[11] Li X.-Y., Sun Y.-M., Yuan J.-W. (2018). Metal-free catalyzed arylsulfonylation of chloroquinoline with sodium arylsulfinates under microwave irradiation. Z. Naturforschung B.

[12] Liu N.-W., Hofman K., Herbert A., Manolikakes G. (2018). Visible-Light Photoredox/Nickel Dual Catalysis for the Cross-Coupling of Sulfinic Acid Salts with Aryl Iodides. Org. Lett..

[13] Liu X.-W., Wang J.-Q., Ma H., Zhu Q., Xie L.-Y. (2021). Ball-milling synthesis of sulfonyl quinolines via coupling of haloquinolines with sulfonic acids. Green Chem..

[14] Maloney K.M., Kuethe J.T., Linn K. (2011). A Practical, One-Pot Synthesis of Sulfonylated Pyridines. Org. Lett..

[15] Nguyen V.D., Nguyen V.T., Haug G.C., Dang H.T., Arman H.D., Ermler W.C., Larionov O.V. (2019). Rapid and Chemodivergent Synthesis of N-Heterocyclic Sulfones and Sulfides: Mechanistic and Computational Details of the Persulfate-Initiated Catalysis. ACS Catal..

[16] Xie L.-Y., Peng S., Tan J.-X., Sun R.-X., Yu X., Dai N.-N., Tang Z.-L., Xu X., He W.-M. (2018). Waste-Minimized Protocol for the Synthesis of Sulfonylated N-Heteroaromatics in Water. ACS Sustain. Chem. Eng..

[17] Ai C., Liao X., Zhou Y., Yan Z., Lin S. (2020). SO_2_F_2_-mediated deoxygenative C2-sulfonylation of quinoline *N*-oxides with sodium sulfinates. Tetrahedron Lett..

[18] Dong D.-Q., Li L.-X., Li G.-H., Deng Q., Wang Z.-L., Long S. (2019). Visible-light-induced deoxygenative C2-sulfonylation of quinoline *N*-oxides with sulfinic acids for the synthesis of 2-sulfonylquinoline via radical reactions. Chin. J. Catal..

[19] Du B., Qian P., Wang Y., Mei H., Han J., Pan Y. (2016). Cu-Catalyzed Deoxygenative C2-Sulfonylation Reaction of Quinoline *N*-Oxides with Sodium Sulfinate. Org. Lett..

[20] Jiang M., Yuan Y., Wang T., Xiong Y., Li J., Guo H., Lei A. (2019). Exogenous-oxidant- and catalyst-free electrochemical deoxygenative C2 sulfonylation of quinoline *N*-oxides. Chem. Commun..

[21] Li G.-H., Dong D.-Q., Deng Q., Yan S.-Q., Wang Z.-L. (2019). Copper-Catalyzed Deoxygenative C2-Sulfonylation of Quinoline *N*-Oxides with DABSO and Phenyldiazonium Tetrafluoroborates for the Synthesis of 2-Sulfonylquinolines via a Radical Reaction. Synthesis.

[22] Sumunnee L., Buathongjan C., Pimpasri C., Yotphan S. (2017). Iodine/TBHP-Promoted One-Pot Deoxygenation and Direct 2-Sulfonylation of Quinoline *N*-Oxides with Sodium Sulfinates: Facile and Regioselective Synthesis of 2-Sulfonylquinolines. Eur. J. Org. Chem..

[23] Wang R., Zeng Z., Chen C., Yi N., Jiang J., Cao Z., Deng W., Xiang J. (2016). Fast regioselective sulfonylation of pyridine/quinoline *N*-oxides induced by iodine. Org. Biomol. Chem..

[24] You G., Xi D., Sun J., Hao L., Xia C. (2019). Transition-metal- and oxidant-free three-component reaction of quinoline *N*-oxides, sodium metabisulfite and aryldiazonium tetrafluoroborates via a dual radical coupling process. Org. Biomol. Chem..

[25] Xie L.-Y., Fang T.-G., Tan J.-X., Zhang B., Cao Z., Yang L.-H., He W.-M. (2019). Visible-light-induced deoxygenative C2-sulfonylation of quinoline *N*-oxides with sulfinic acids. Green Chem..

[26] Xie L.-Y., Li Y.-J., Qu J., Duan Y., Hu J., Liu K.-J., Cao Z., He W.-M. (2017). A base-free, ultrasound accelerated one-pot synthesis of 2-sulfonylquinolines in water. Green Chem..

[27] Sun K., Chen X.-L., Li X., Qu L.-B., Bi W.-Z., Chen X., Ma H.-L., Zhang S.-T., Han B.-W., Zhao Y.-F. (2015). H-phosphonate-mediated sulfonylation of heteroaromatic *N*-oxides: A mild and metal-free one-pot synthesis of 2-sulfonyl quinolines/pyridines. Chem. Commun..

[28] Peng S., Song Y.-X., He J.-Y., Tang S.-S., Tan J.-X., Cao Z., Lin Y.-W., He W.-M. (2019). TsCl-promoted sulfonylation of quinoline *N*-oxides with sodium sulfinates in water. Chin. Chem. Lett..

[29] Malykhin R.S., Sukhorukov A.Y. (2021). Nucleophilic Halogenation of Heterocyclic *N*-Oxides: Recent Progress and a Practical Guide. Adv. Synth. Catal..

[30] Qiao K., Wan L., Sun X., Zhang K., Zhu N., Li X., Guo K. (2016). Regioselective Chlorination of Quinoline *N*-Oxides and Isoquinoline *N*-Oxides Using PPh_3_/Cl_3_CCN. Eur. J. Org. Chem..

[31] Wang D., Jia H., Wang W., Wang Z. (2014). A practical and mild chlorination of fused heterocyclic *N*-oxides. Tetrahedron Lett..

[32] Bi W.-Z., Sun K., Qu C., Chen X.-L., Qu L.-B., Zhu S.-H., Li X., Wu H.-T., Duan L.-K., Zhao Y.-F. (2017). A direct metal-free C2–H functionalization of quinoline *N*-oxides: A highly selective amination and alkylation strategy towards 2-substituted quinolines. Org. Chem. Front..

[33] Zhang Z., Pi C., Tong H., Cui X., Wu Y. (2017). Iodine-Catalyzed Direct C–H Alkenylation of Azaheterocycle *N*-Oxides with Alkenes. Org. Lett..

[34] Wu J., Cui X., Chen L., Jiang G., Wu Y. (2009). Palladium-Catalyzed Alkenylation of Quinoline-*N*-oxides via C−H Activation under External-Oxidant-Free Conditions. J. Am. Chem. Soc..

[35] Xia H., Liu Y., Zhao P., Gou S., Wang J. (2016). Synthesis of 2-Alkenylquinoline by Reductive Olefination of Quinoline *N*-Oxide under Metal-Free Conditions. Org. Lett..

[36] Londregan A.T., Jennings S., Wei L. (2011). Mild Addition of Nucleophiles to Pyridine-*N*-Oxides. Org. Lett..

[37] Bi W.-Z., Qu C., Chen X.-L., Qu L.-B., Liu Z.-D., Sun K., Li X., Zhao Y.-F. (2017). A Direct C2-Selective Phenoxylation and Alkoxylation of Quinoline *N*-Oxides with Various Phenols and Alcohols in the Presence of H-Phosphonate. Eur. J. Org. Chem..

[38] Lian Y., Coffey S.B., Li Q., Londregan A.T. (2016). Preparation of Heteroaryl Ethers from Azine *N*-Oxides and Alcohols. Org. Lett..

[39] Zhang D., Qiao K., Hua J., Liu Z., Qi H., Yang Z., Zhu N., Fang Z., Guo K. (2018). Preparation of fluoroalkoxy or fluorophenoxy substituted *N*-heterocycles from heterocyclic *N*-oxides and polyfluoroalcohols. Org. Chem. Front..

[40] Chen X., Li X., Qu Z., Ke D., Qu L., Duan L., Mai W., Yuan J., Chen J., Zhao Y. (2014). H-Phosphonate-Mediated Amination of Quinoline *N*-Oxides with Tertiary Amines: A Mild and Metal-Free Synthesis of 2-Dialkylaminoquinolines. Adv. Synth. Catal..

[41] Londregan A.T., Jennings S., Wei L. (2010). General and Mild Preparation of 2-Aminopyridines. Org. Lett..

[42] Yin J., Xiang B., Huffman M.A., Raab C.E., Davies I.W. (2007). A General and Efficient 2-Amination of Pyridines and Quinolines. J. Org. Chem..

[43] Bu C., Wang K., Gong C., Wang D. (2024). Green and fast 2-aryloxylation/amination of quinolines. Green Chem..

[44] Hu C., Liu R., Ning Z., Mou D., Fu Y., Du Z. (2022). TsCl-promoted thiolation of quinoline *N*-oxides with thiophenols. Org. Biomol. Chem..

[45] Sarmah B.K., Konwar M., Bhattacharyya D., Adhikari P., Das A. (2019). Regioselective Cyanation of Six-Membered *N*-Heteroaromatic Compounds Under Metal-, Activator-, Base- and Solvent-Free Conditions. Adv. Synth. Catal..

[46] Xu F., Li Y., Huang X., Fang X., Li Z., Jiang H., Qiao J., Chu W., Sun Z. (2019). Hypervalent Iodine(III)-Mediated Regioselective Cyanation of Quinoline *N*-Oxides with Trimethylsilyl Cyanide. Adv. Synth. Catal..

[47] Xie L.-Y., Peng S., Lu L.-H., Hu J., Bao W.-H., Zeng F., Tang Z., Xu X., He W.-M. (2018). Brønsted Acidic Ionic Liquid-Promoted Amidation of Quinoline *N*-Oxides with Nitriles. ACS Sustain. Chem. Eng..

[48] Wu J., Xia Z. (2023). Gold-Catalyzed Redox-Neutral Reaction of Nitriles with Quinoline *N*-Oxides. Adv. Synth. Catal..

[49] Chen X., Cui X., Yang F., Wu Y. (2015). Base-Promoted Cross-Dehydrogenative Coupling of Quinoline *N*-Oxides with 1,3-Azoles. Org. Lett..

[50] Tsunokawa R., Karuo Y., Tarui A., Sato K., Hosoya T., Agou T., Kawai K., Omote M. (2023). Metal-Free Arylation of Pyridine/Quinoline *N*-Oxides with Indoles. Eur. J. Org. Chem..

[51] Singha K., Habib I., Hossain M. (2022). Quinoline *N*-Oxide: A Versatile Precursor in Organic Transformations. ChemistrySelect.

[52] Kutasevich A.V., Perevalov V.P., Mityanov V.S. (2021). Recent Progress in Non-Catalytic C–H Functionalization of Heterocyclic *N*-Oxides. Eur. J. Org. Chem..

[53] Singh J., Patel R.I., Sharma A. (2022). Visible-Light-Mediated C-2 Functionalization and Deoxygenative Strategies in Heterocyclic *N*-Oxides. Adv. Synth. Catal..

[54] Wang D., Désaubry L., Li G., Huang M., Zheng S. (2021). Recent Advances in the Synthesis of C2-Functionalized Pyridines and Quinolines Using *N*-Oxide Chemistry. Adv. Synth. Catal..

[55] Wu L.-Y., Tian H., Tian Z.-Y., Xu X.-Q., Peng S., Xie L.-Y. (2024). TsCl promoted deoxygenative phosphorothiolation of quinoline *N*-oxides towards S-quinolyl phosphorothioates. Org. Biomol. Chem..

[56] Xie L.-Y., Liu C., Wang S.-Y., Tian Z.-Y., Peng S. (2024). Ts_2_O mediated deoxygenative C2-dithiocarbamation of quinoline *N*-oxides with CS_2_ and amines. RSC Adv..

[57] Xie L.-Y., Peng S., Liu F., Chen G.-R., Xia W., Yu X., Li W.-F., Cao Z., He W.-M. (2018). Metal-free deoxygenative sulfonylation of quinoline *N*-oxides with sodium sulfinates via a dual radical coupling process. Org. Chem. Front..

[58] Zhao M.-Y., Tang J.-J., Lin Y.-J., Tian Z.-Y., Peng S., Xie L.-Y. (2024). Ts_2_O Promoted Deoxygenative C–H Dithiocarbonation of Quinoline *N*-Oxides with Potassium O-Alkyl Xanthates. J. Org. Chem..

[59] Weber W.G., McLeary J.B., Sanderson R.D. (2006). Facile preparation of bis(thiocarbonyl)disulfides via elimination. Tetrahedron Lett..

[60] Chen G., Xu B. (2022). Divergent Synthesis of Sulfonyl Quinolines, Formyl Indoles, and Quinolones from Ethynyl Benzoxazinanones via AuI Catalysis, AuI-ArI Co-Catalysis, and Silver Catalysis. ACS Catal..

