# Supplementary material for: An Unexpected Synthesis of 2-Sulfonylquinolines via Deoxygenative C2-Sulfonylation of Quinoline N-Oxides with Sulfonyl Chlorides
Source: Molecules. 2024 Jun 16;29(12):2863. doi: 10.3390/molecules29122863 (PMC11206716; doi:10.3390/molecules29122863)
Supplement: Supplementary file 1 [file molecules-29-02863-s001.zip › molecules-3055372-supplementary.pdf]

Supporting Information

An Unexpected Synthesis of 2-Sulfonylquinolines via Deoxygenative C2-Sulfonylation of Quinoline *N*-oxides with Sulfonyl Chlorides

Wei Yang[#], Zhong-Ying Tian[#], Ying-Jun Lin[#], and Long-Yong Xie*

Key Laboratory of Comprehensive Utilization of Advantage Plants Resources of Southern Hunan, College of Chemistry and Bioengineering, Hunan University of Science and Engineering, Yongzhou 425100, China

E-mail: longyongxie@yeah.net

Copies of NMR spectra for all products **S2**

Copies of NMR spectra for all products

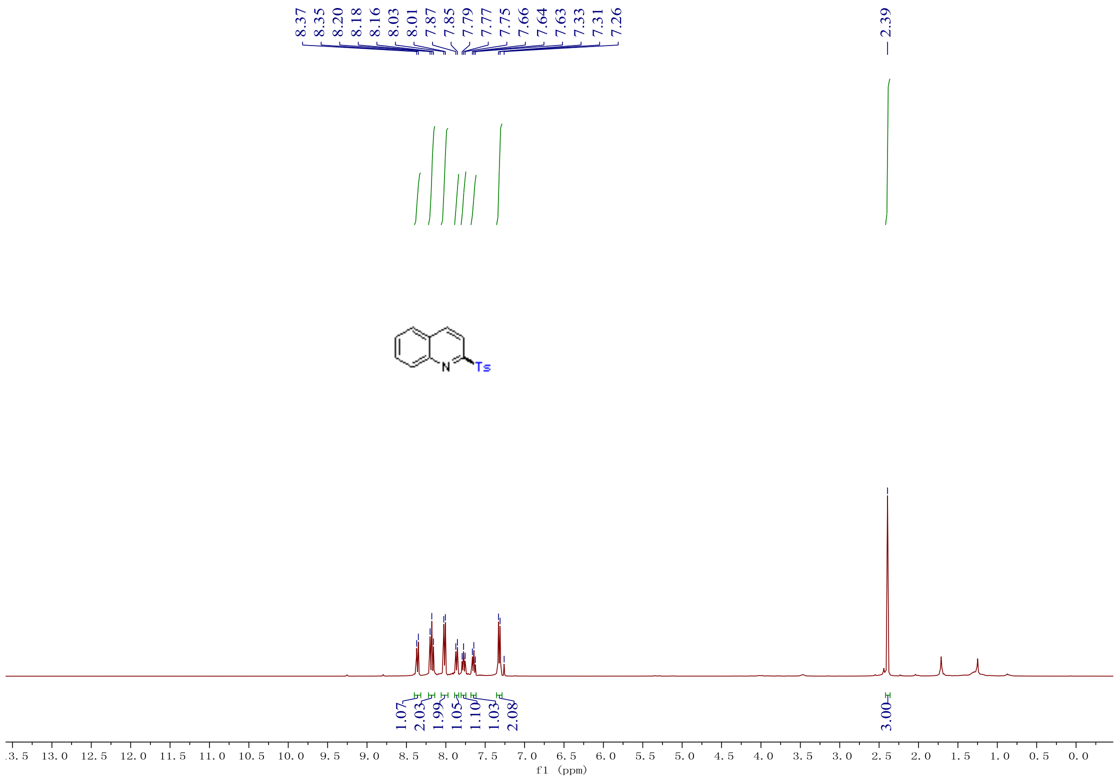

^{1}H NMR ($CDCl_3$, 400 M) spectra of **3aa**

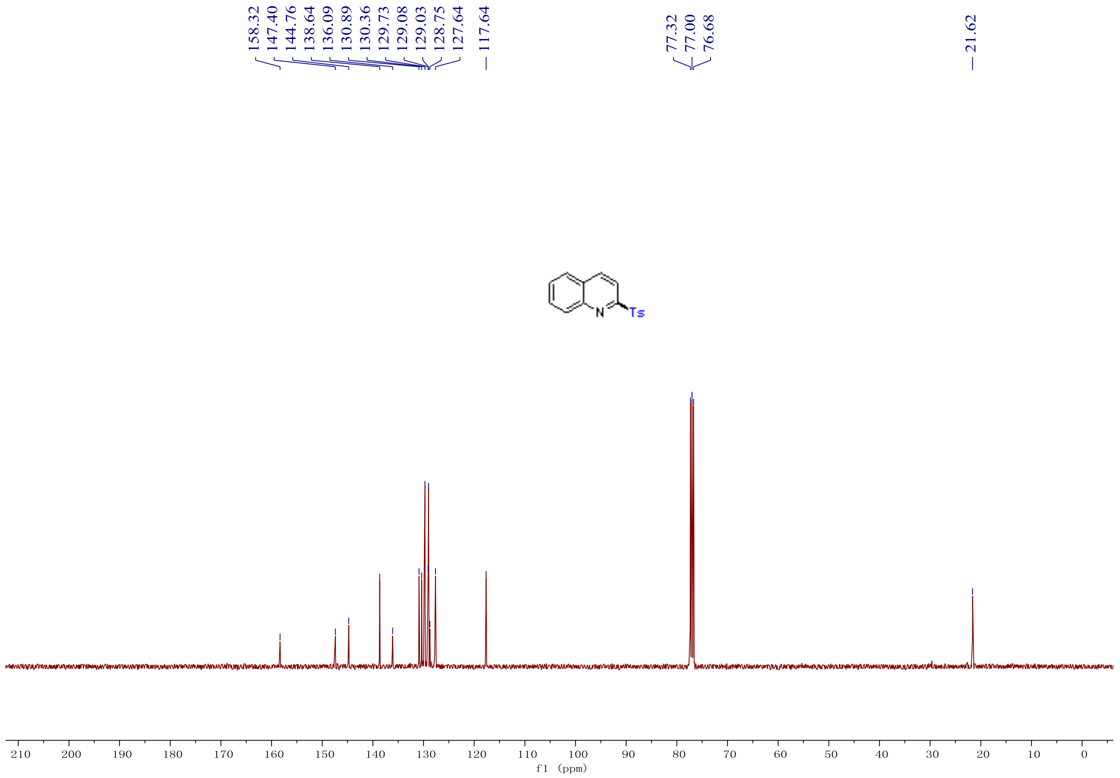

^{13}C NMR ($CDCl_3$, 100 M) spectra of **3aa**

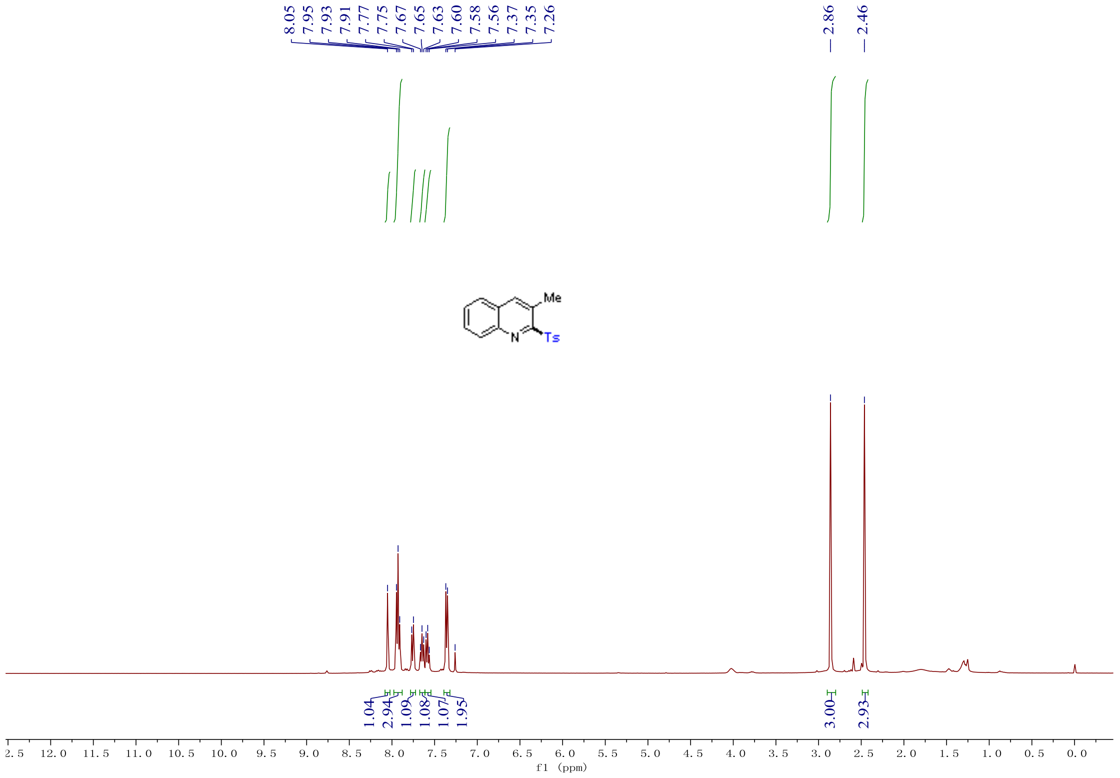

^{1}H NMR ($CDCl_3$, 400 M) spectra of **3ba**

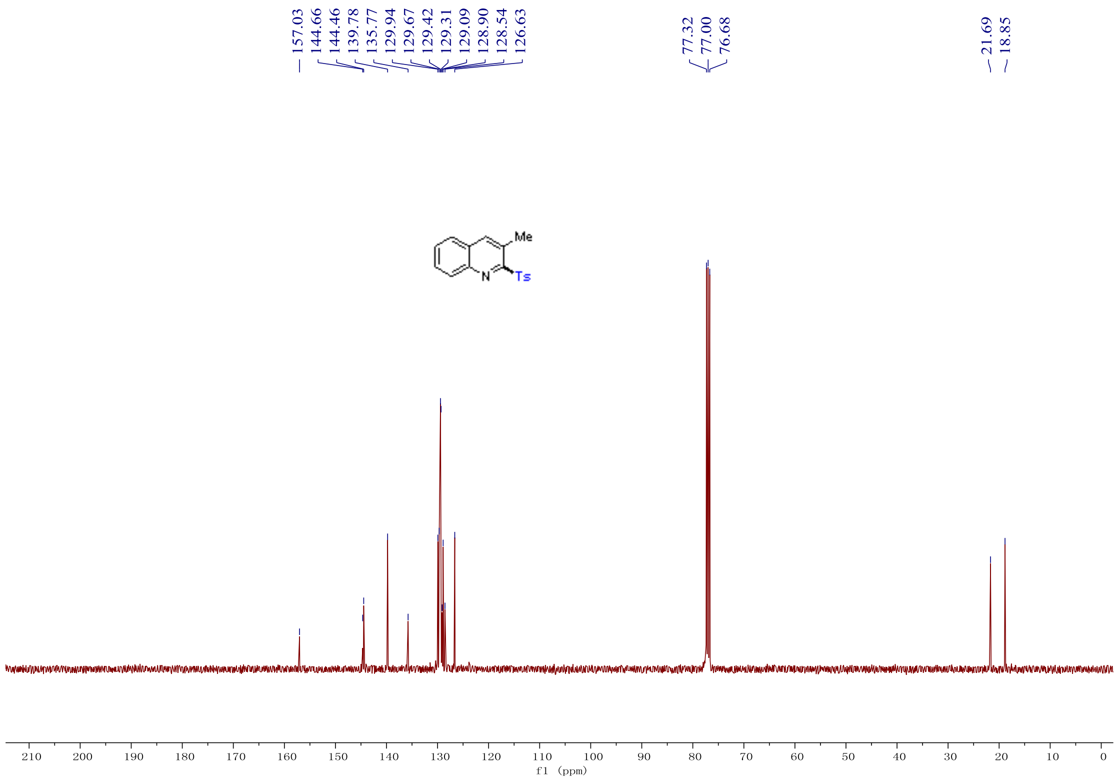

^{13}C NMR ($CDCl_3$, 100 M) spectra of **3ba**

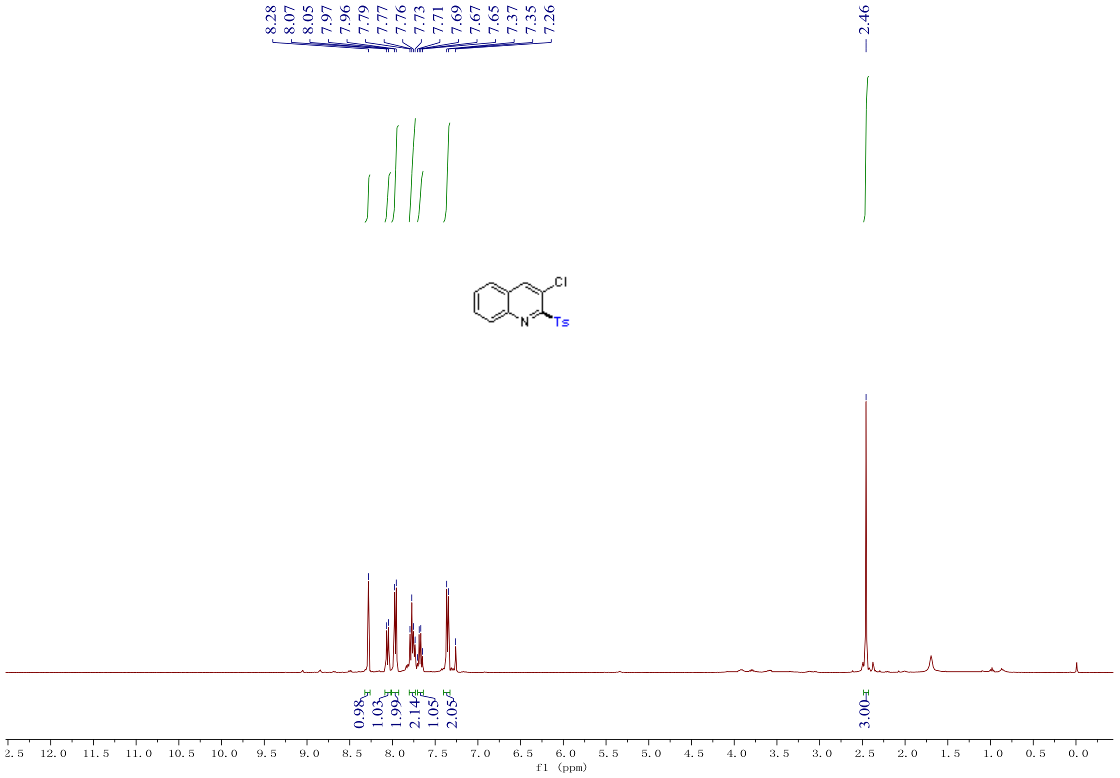

^{1}H NMR ($CDCl_3$, 400 M) spectra of **3ca**

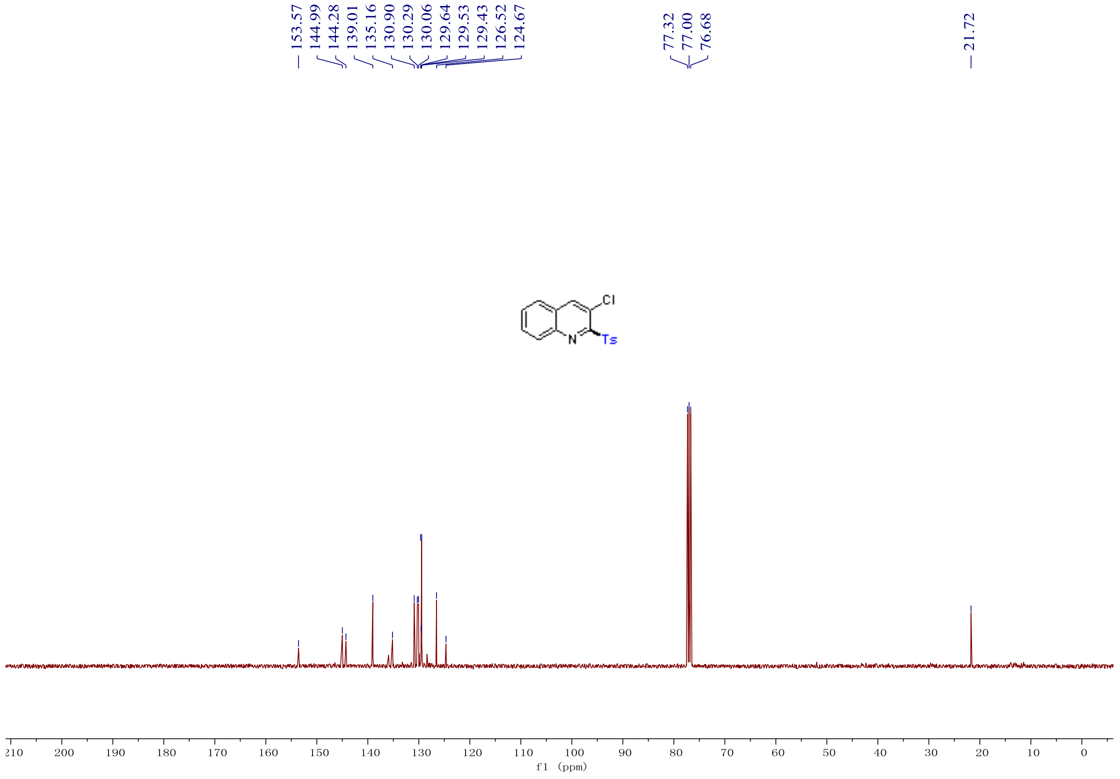

^{13}C NMR ($CDCl_3$, 100 M) spectra of **3ca**

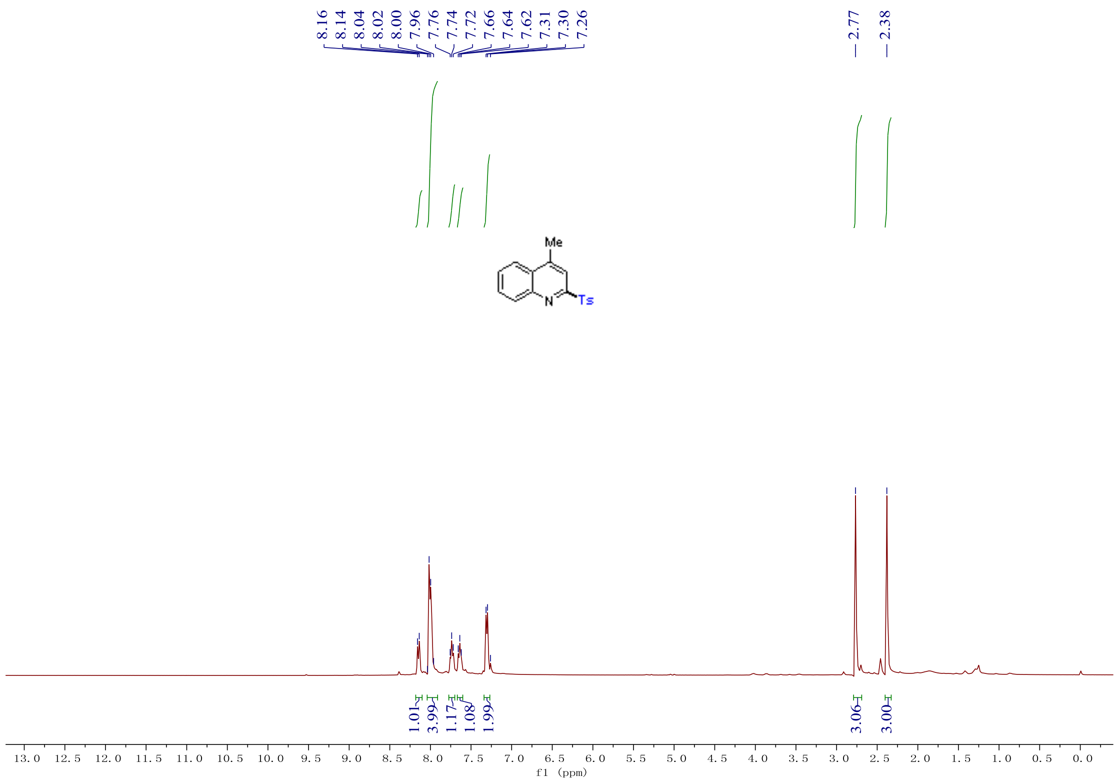

^{1}H NMR ($CDCl_3$, 400 M) spectra of **3da**

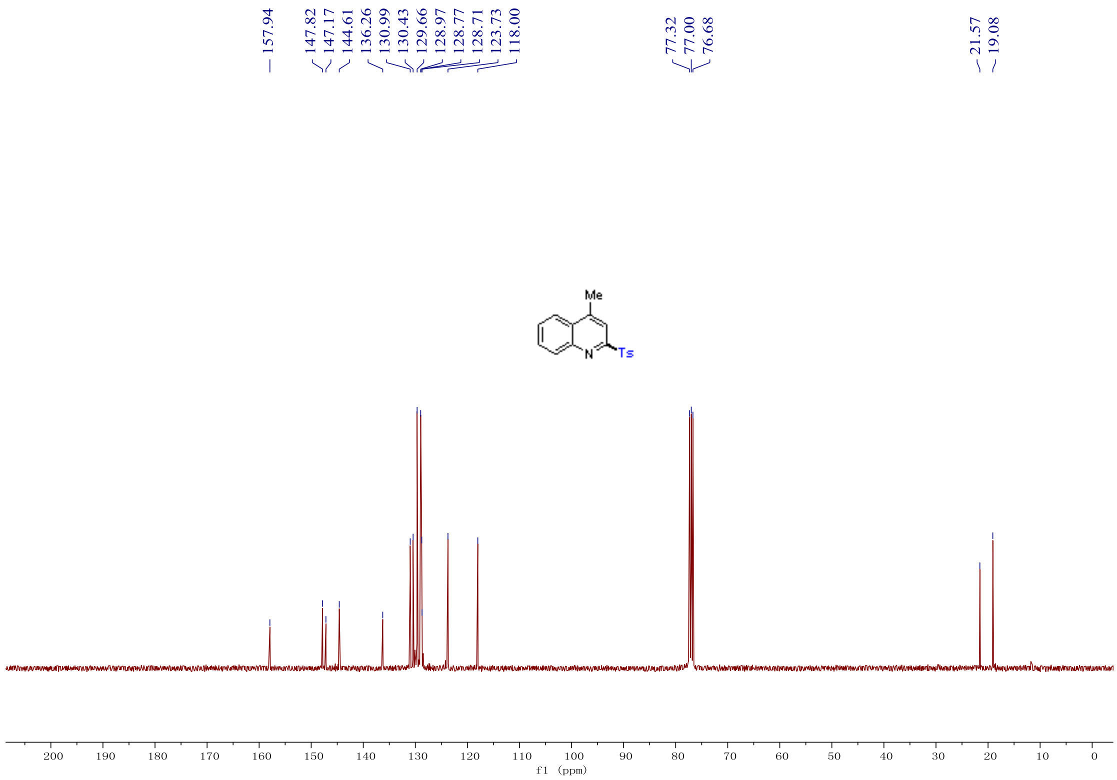

^{13}C NMR ($CDCl_3$, 100 M) spectra of **3da**

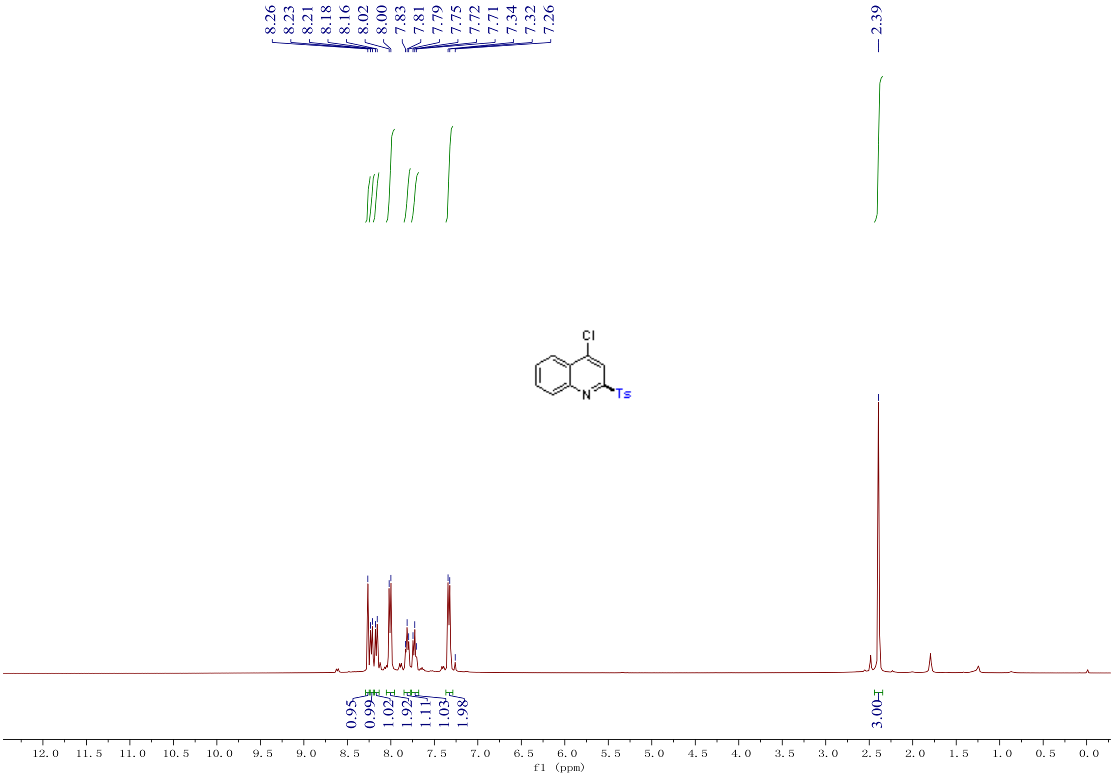

^{1}H NMR ($CDCl_3$, 400 M) spectra of **3ea**

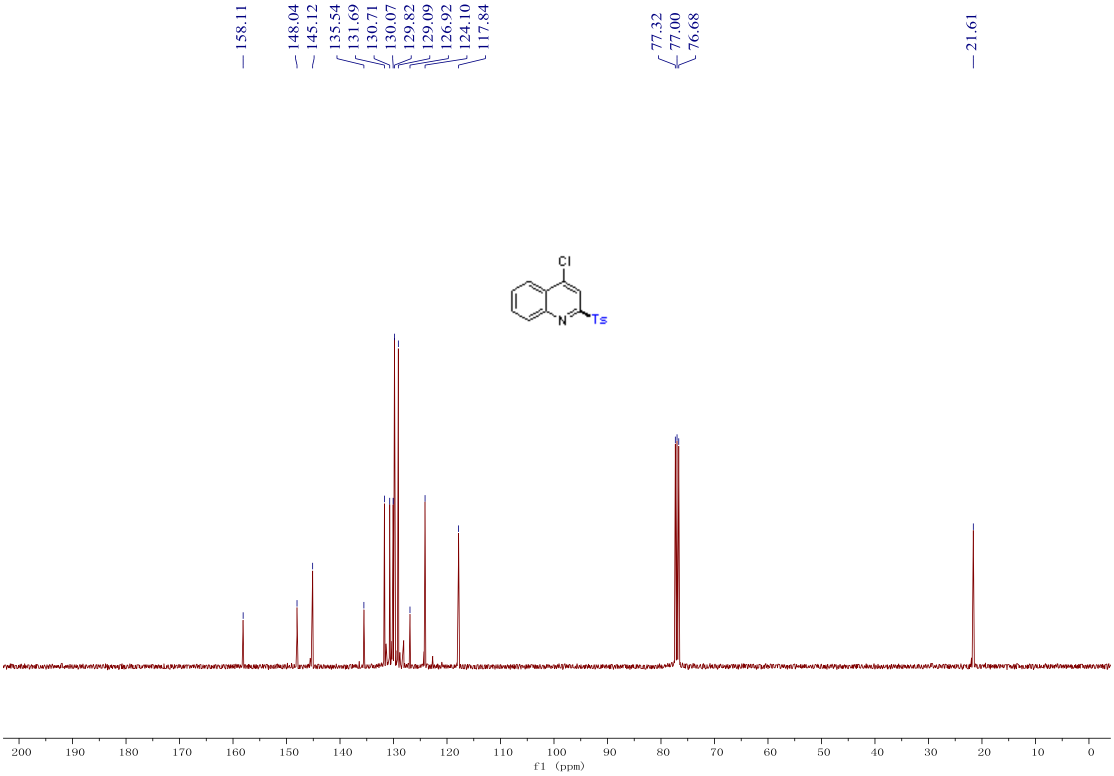

^{13}C NMR ($CDCl_3$, 100 M) spectra of **3ea**

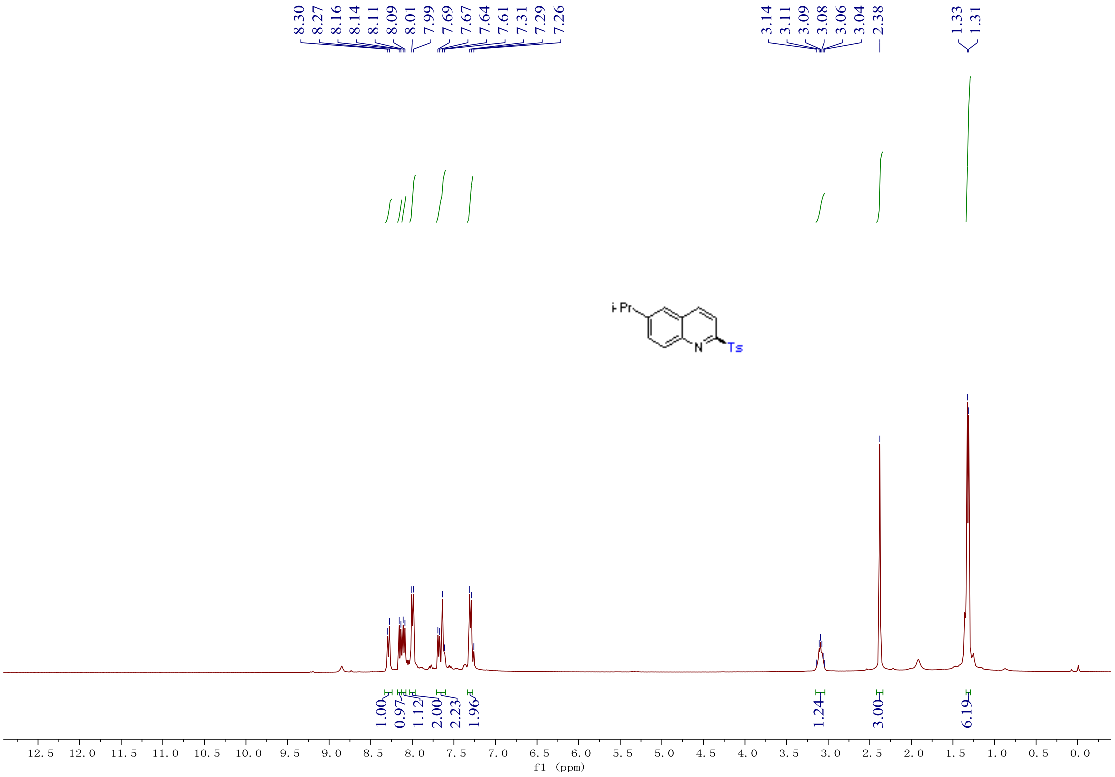

^{1}H NMR ($CDCl_3$, 400 M) spectra of **3fa**

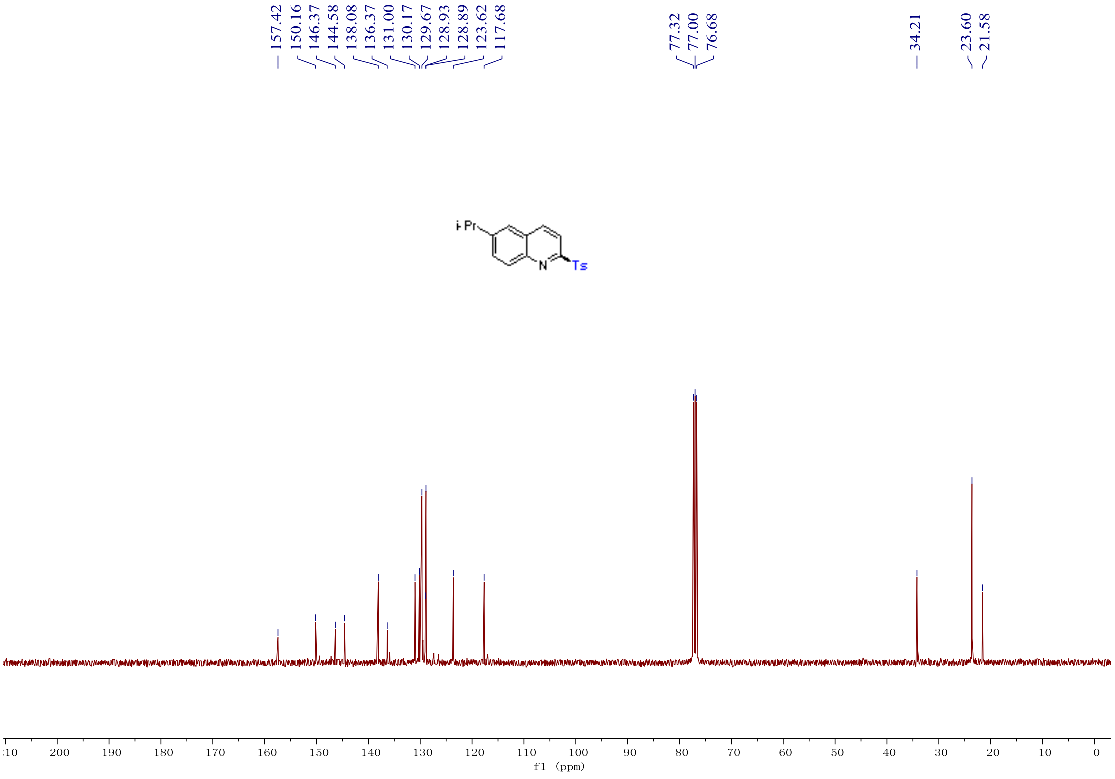

^{13}C NMR ($CDCl_3$, 100 M) spectra of **3fa**

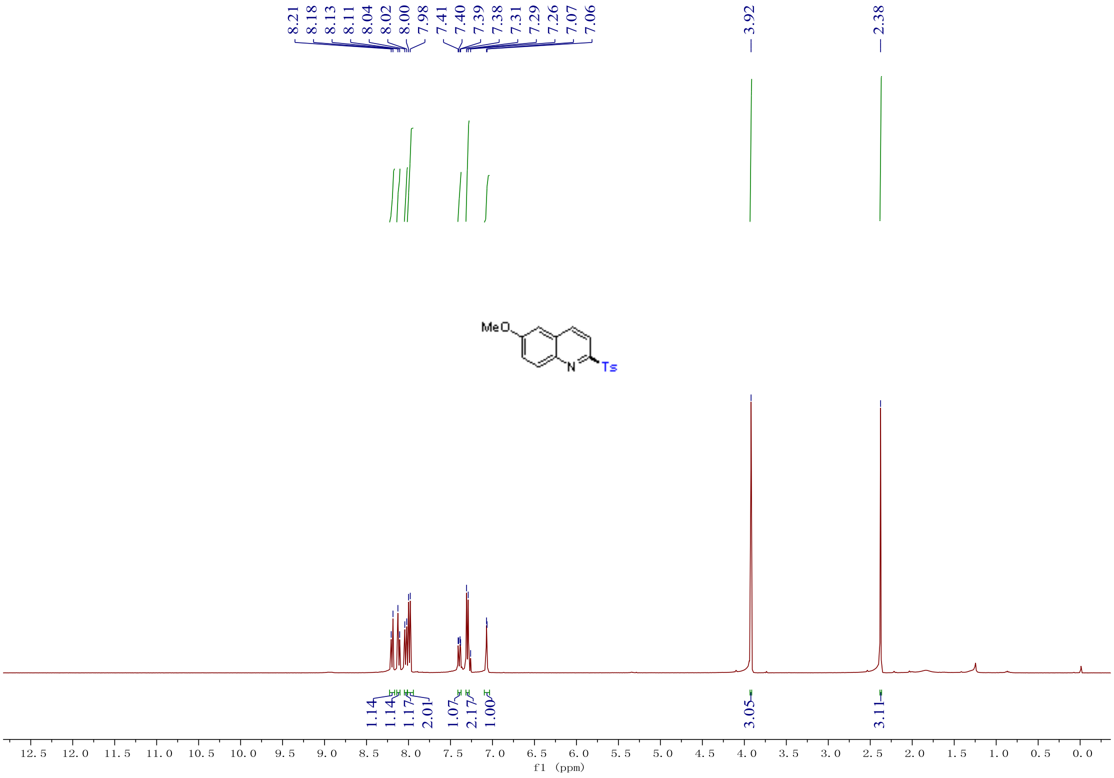

^{1}H NMR ($CDCl_3$, 400 M) spectra of **3ga**

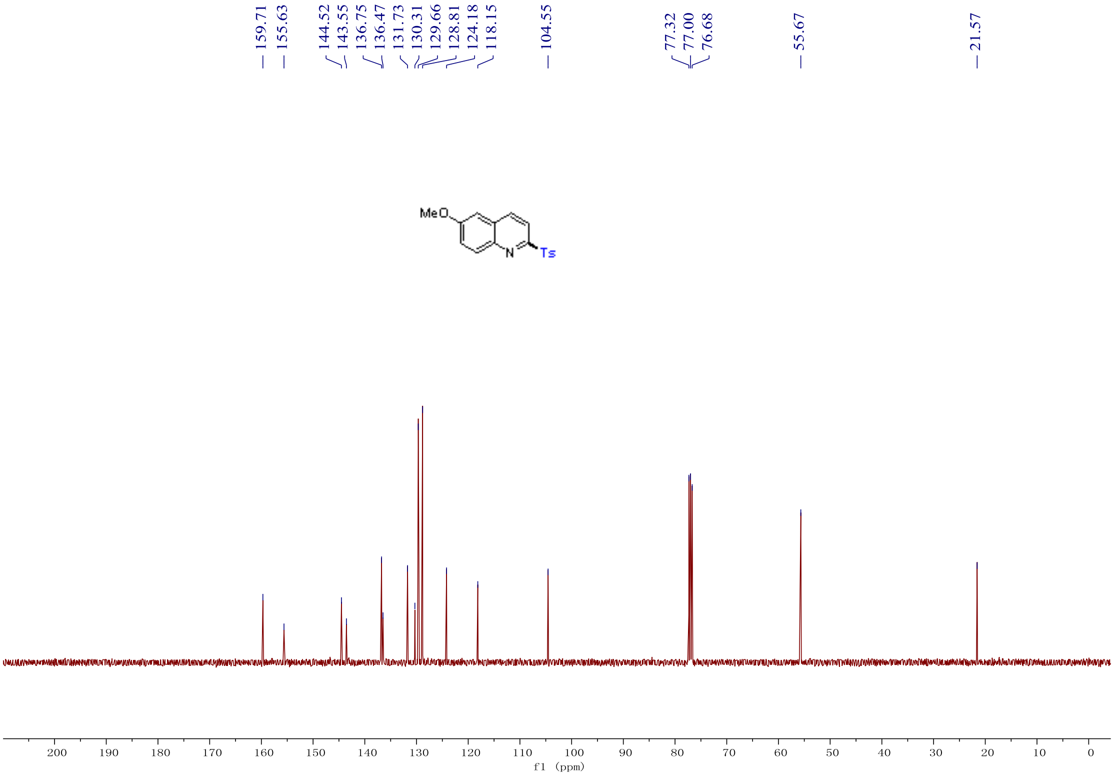

^{13}C NMR ($CDCl_3$, 100 M) spectra of **3ga**

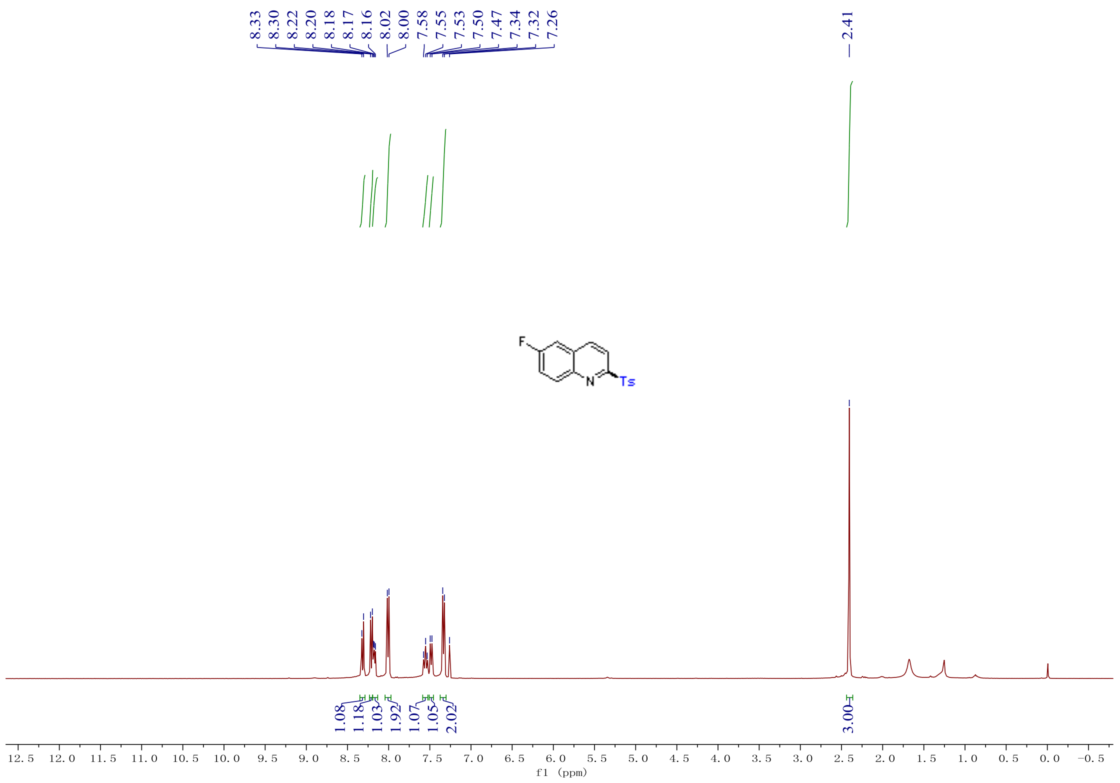

^{1}H NMR ($CDCl_3$, 400 M) spectra of **3ha**

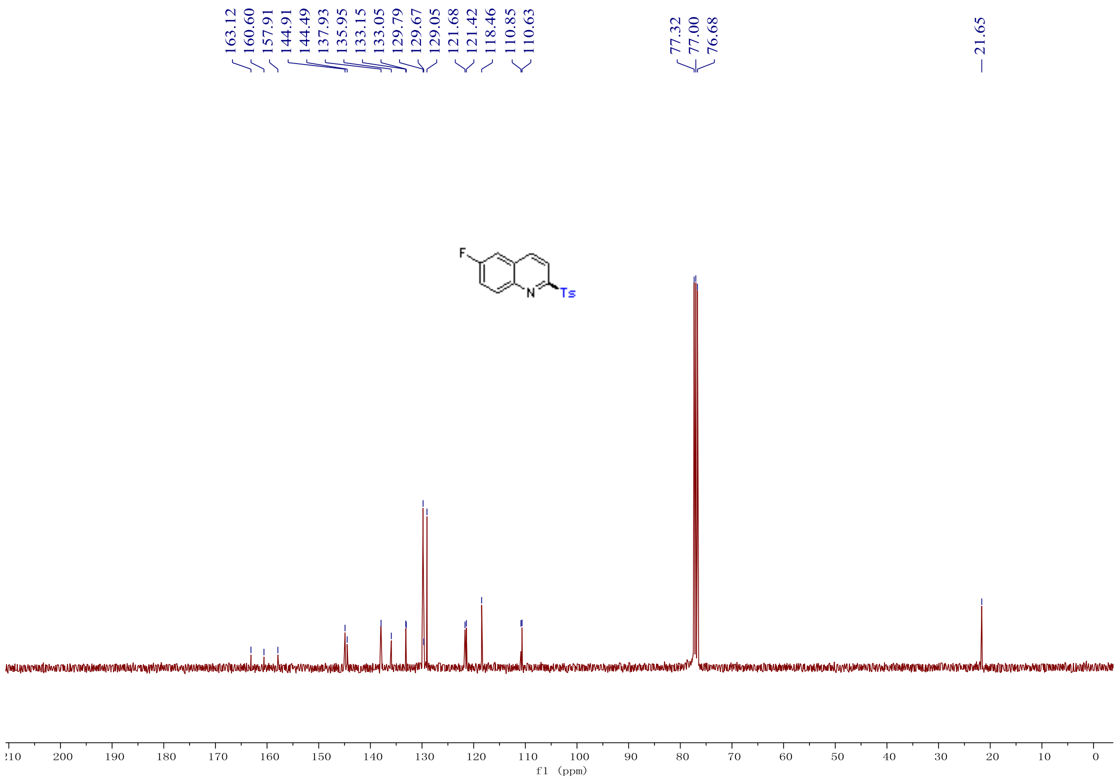

^{13}C NMR ($CDCl_3$, 100 M) spectra of **3ha**

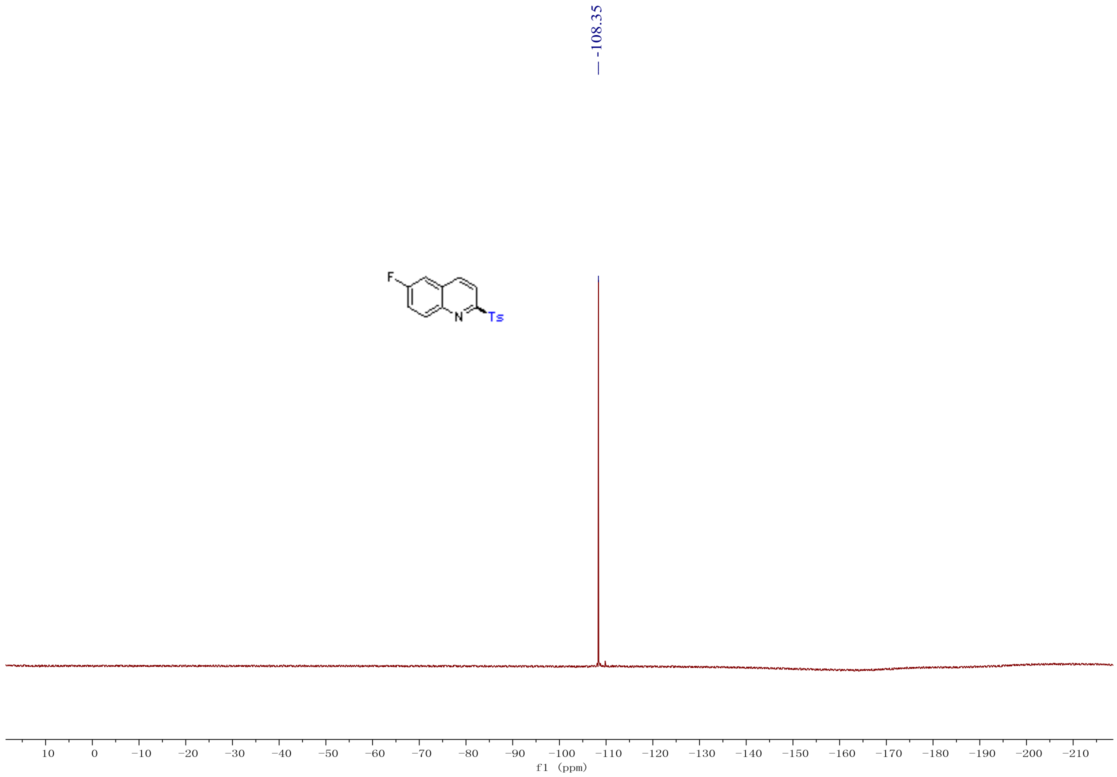

^{19}F NMR ($CDCl_3$, 376 M) spectra of **3ha**

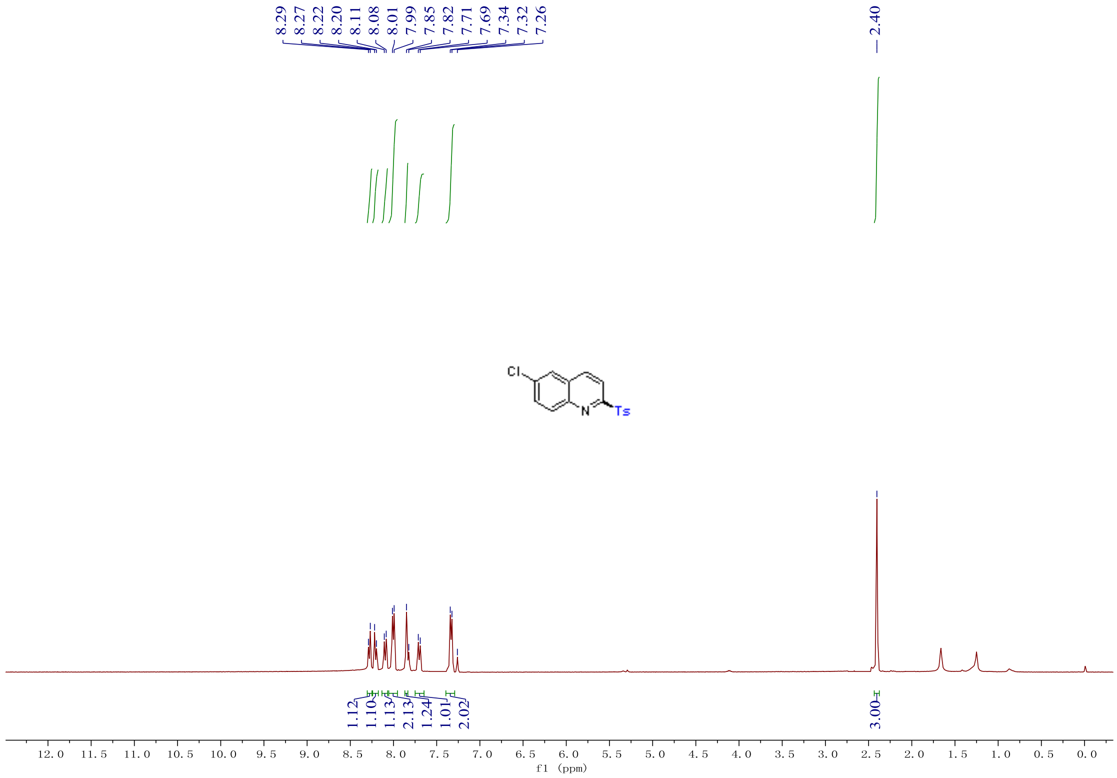

^{1}H NMR ($CDCl_3$, 400 M) spectra of **3ia**

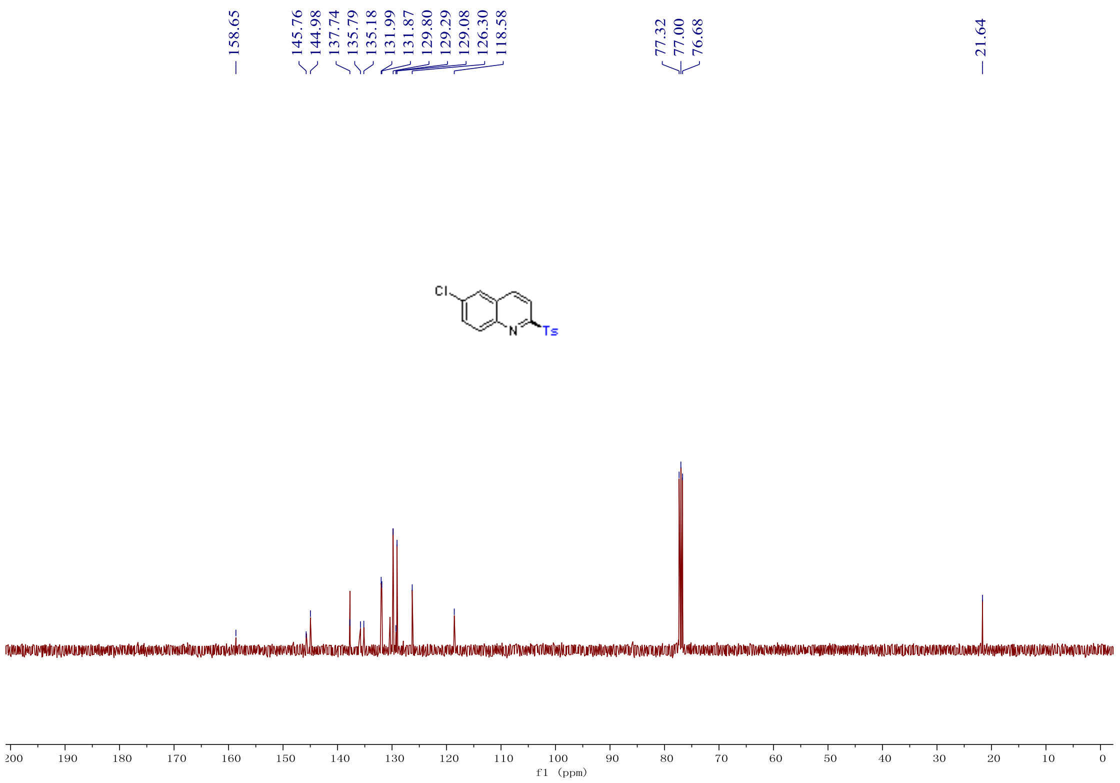

^{13}C NMR ($CDCl_3$, 100 M) spectra of **3ia**

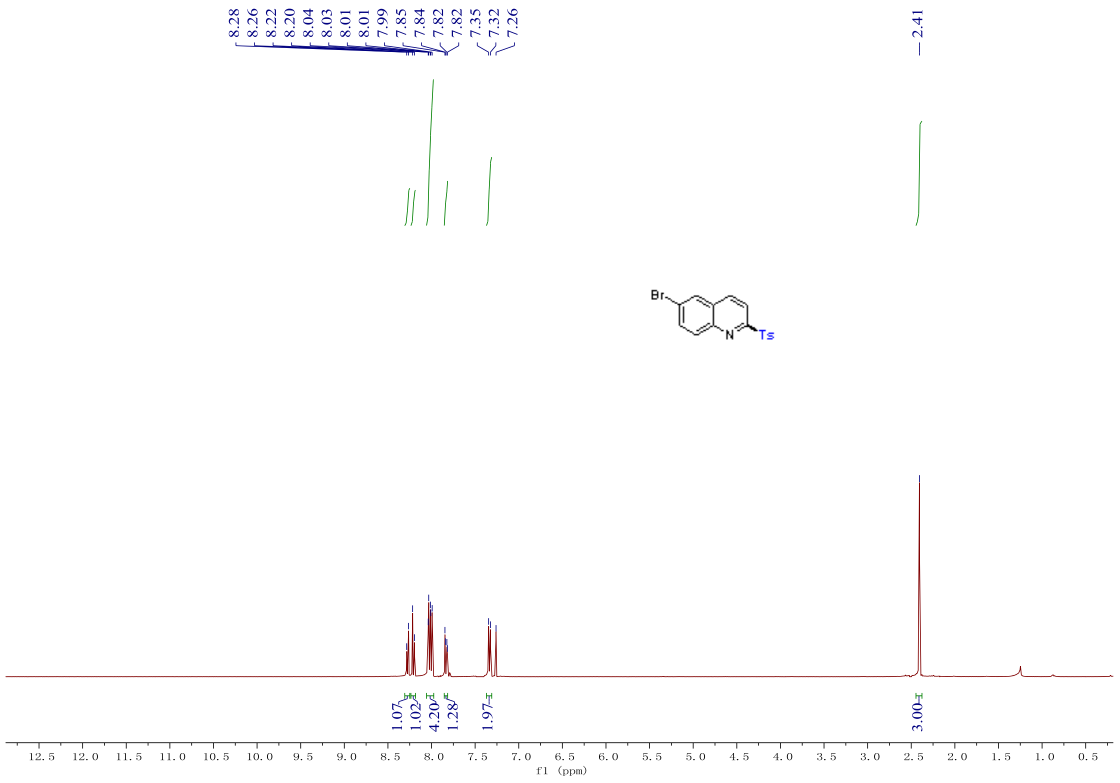

^{1}H NMR ($CDCl_3$, 400 M) spectra of **3ja**

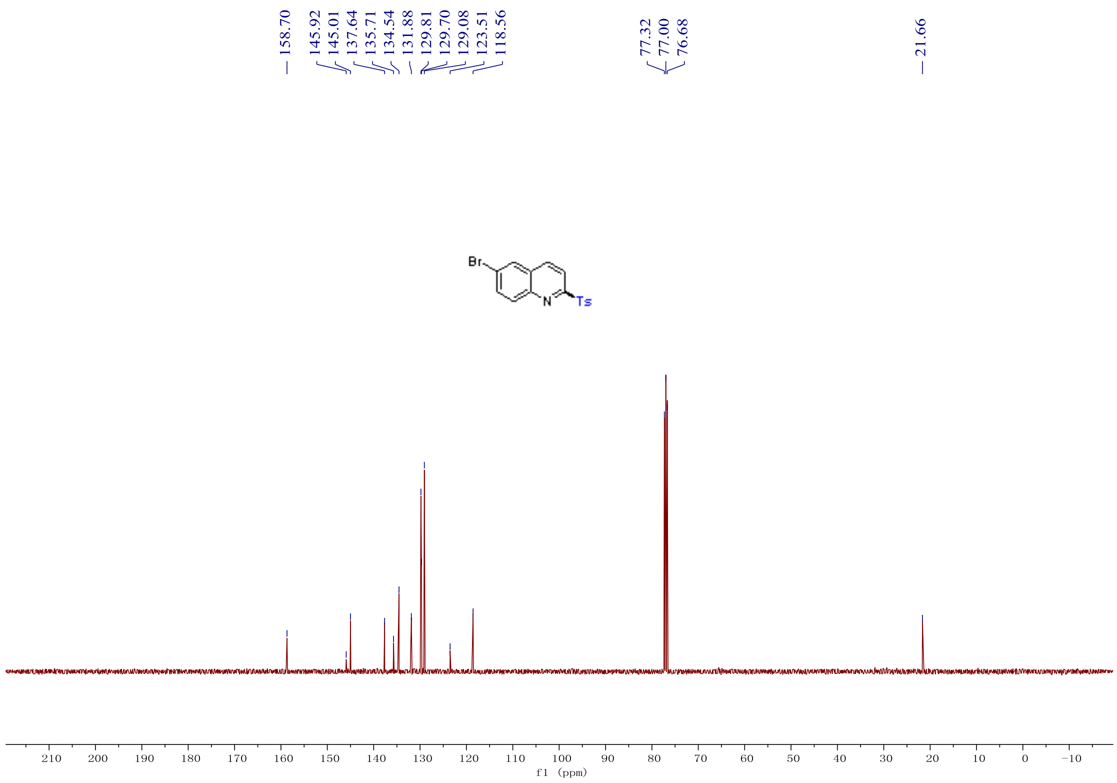

^{13}C NMR ($CDCl_3$, 100 M) spectra of **3ja**

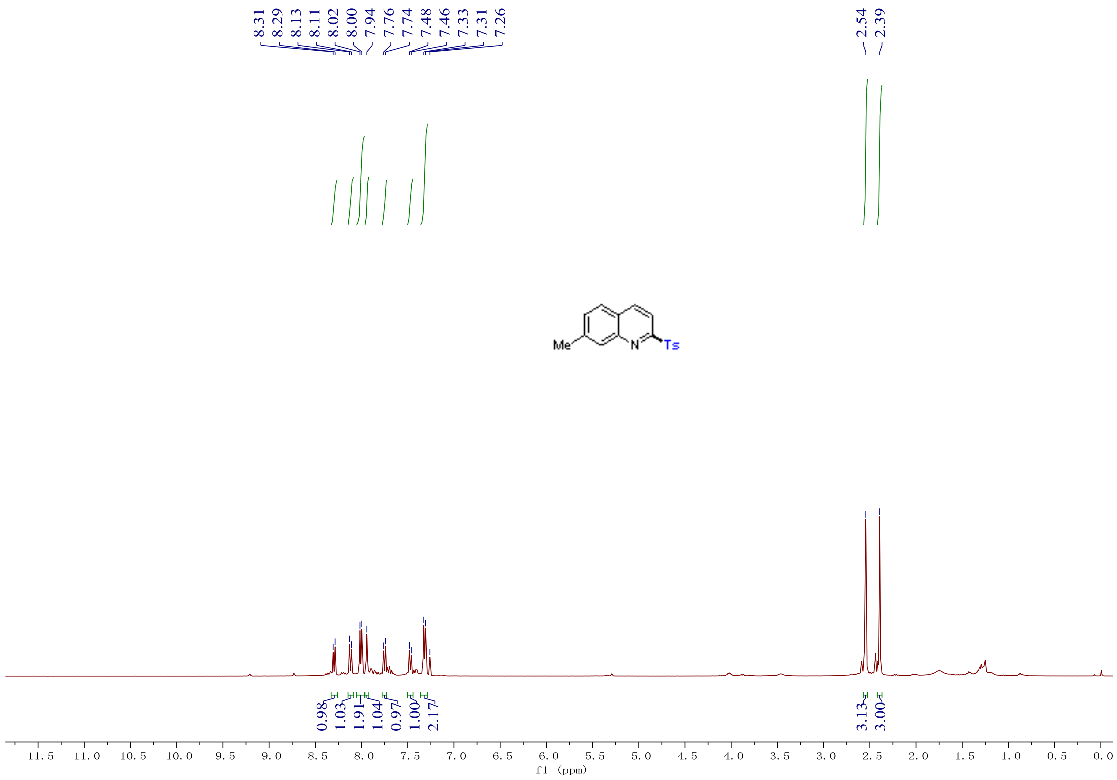

^{1}H NMR ($CDCl_3$, 400 M) spectra of **3ka**

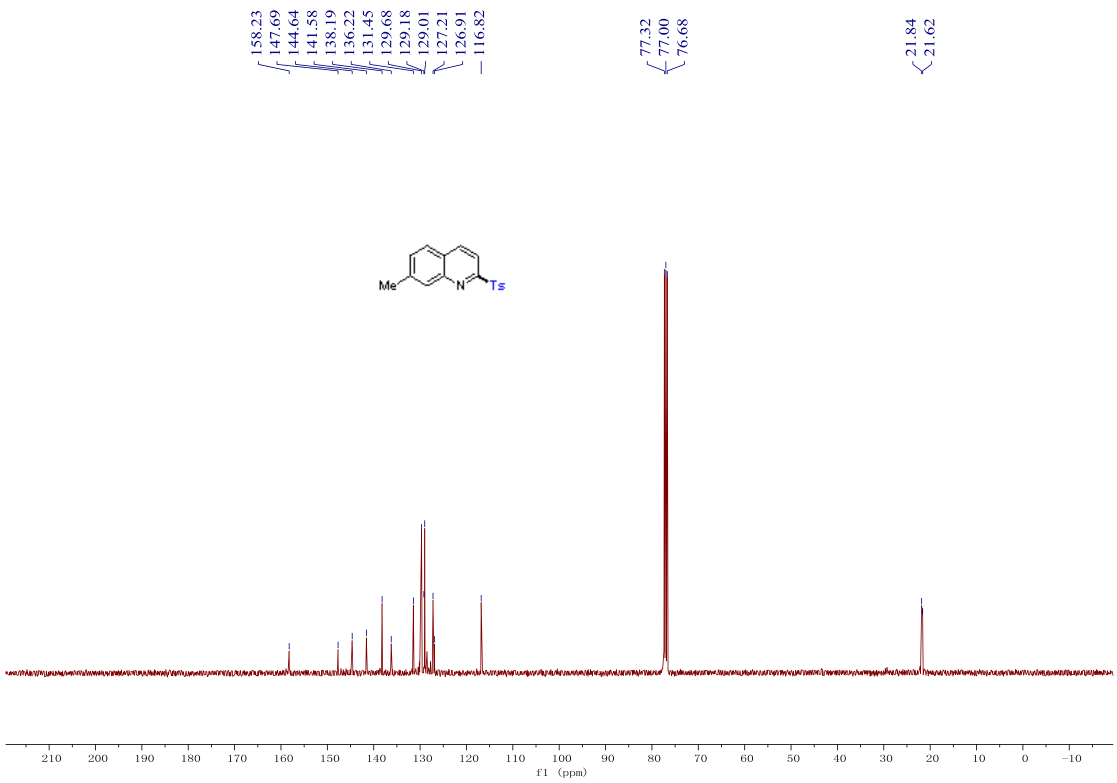

^{13}C NMR ($CDCl_3$, 100 M) spectra of **3ka**

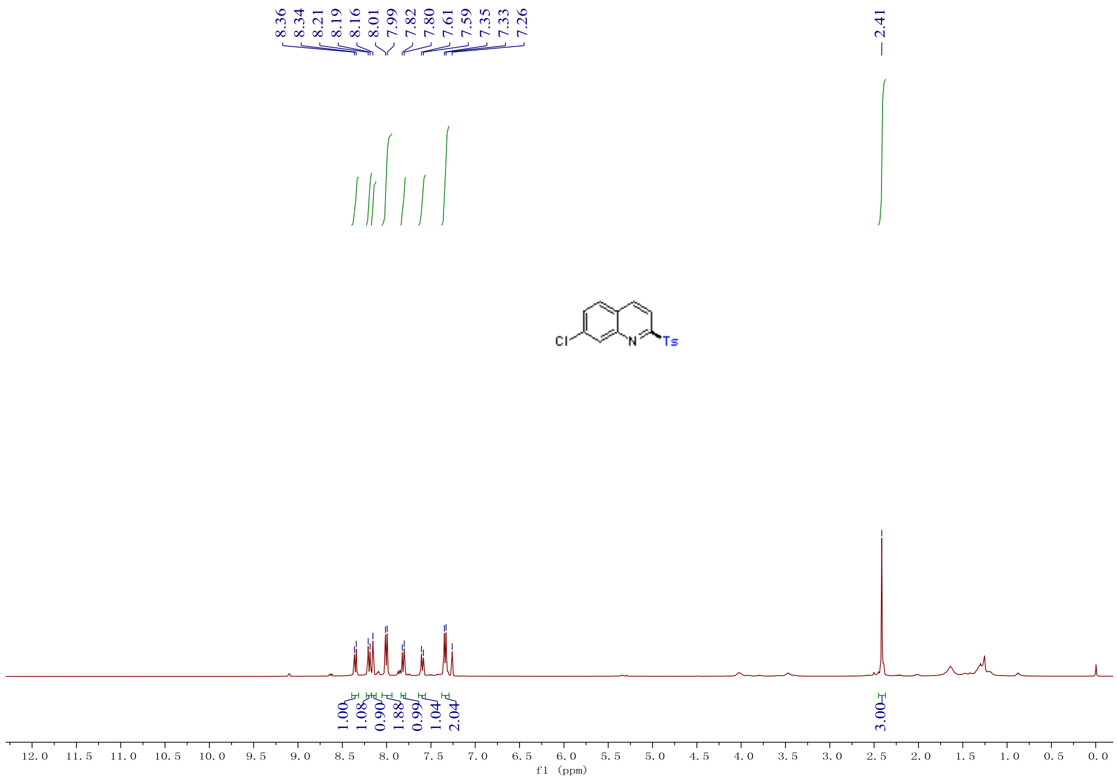

^{1}H NMR ($CDCl_3$, 400 M) spectra of **3la**

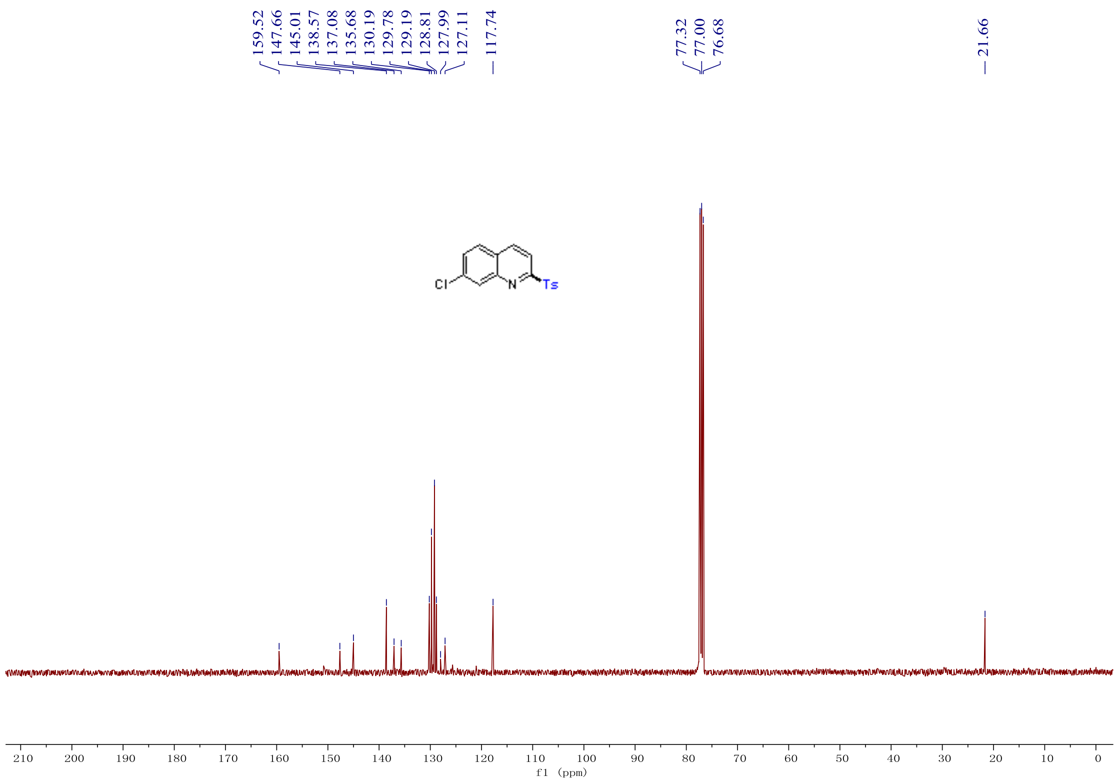

^{13}C NMR ($CDCl_3$, 100 M) spectra of **3la**

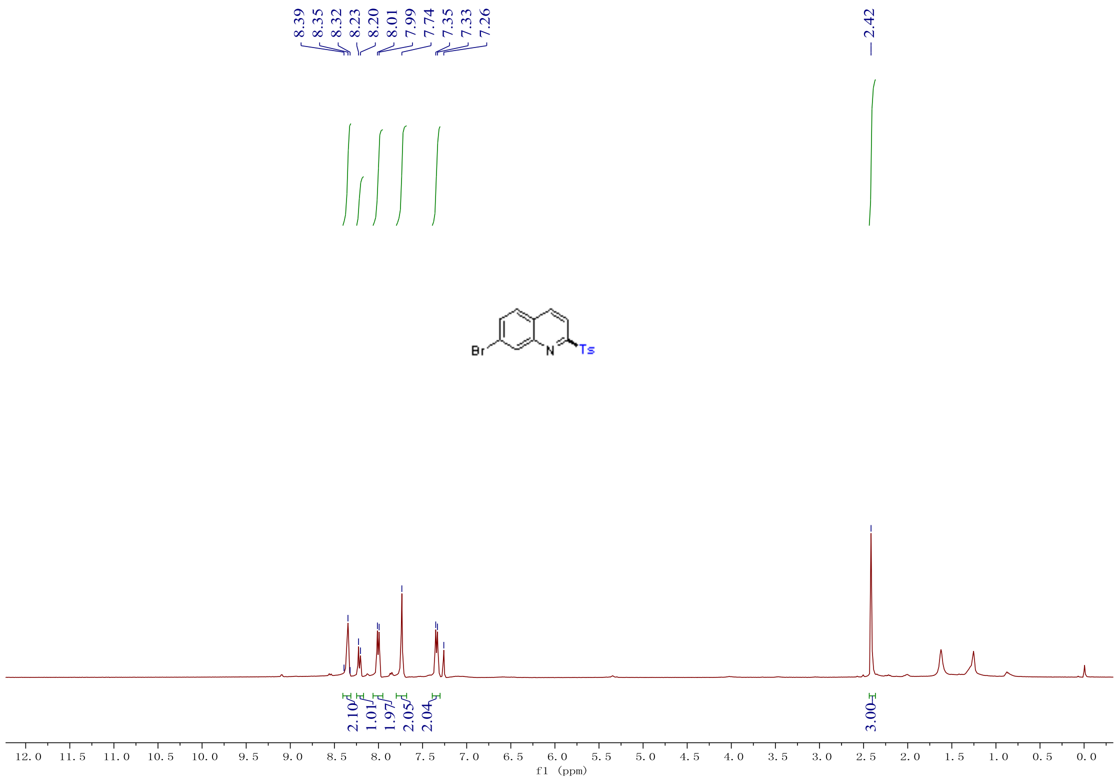

^{1}H NMR ($CDCl_3$, 400 M) spectra of **3ma**

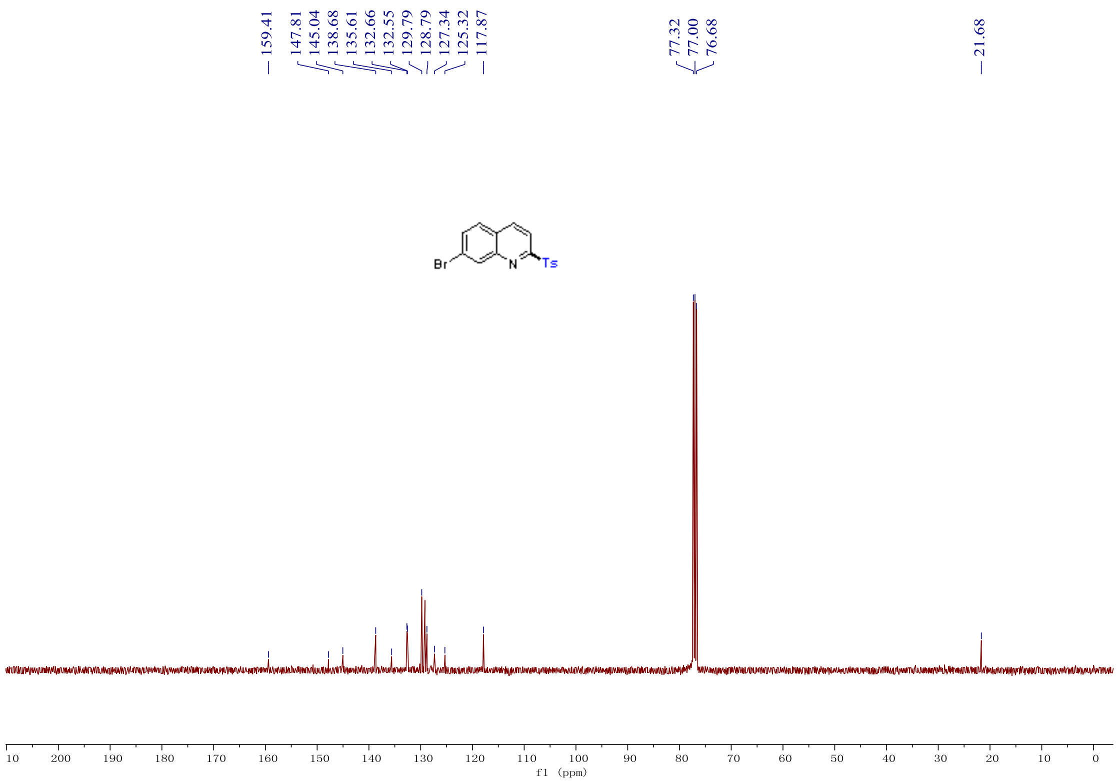

^{13}C NMR ($CDCl_3$, 100 M) spectra of **3ma**

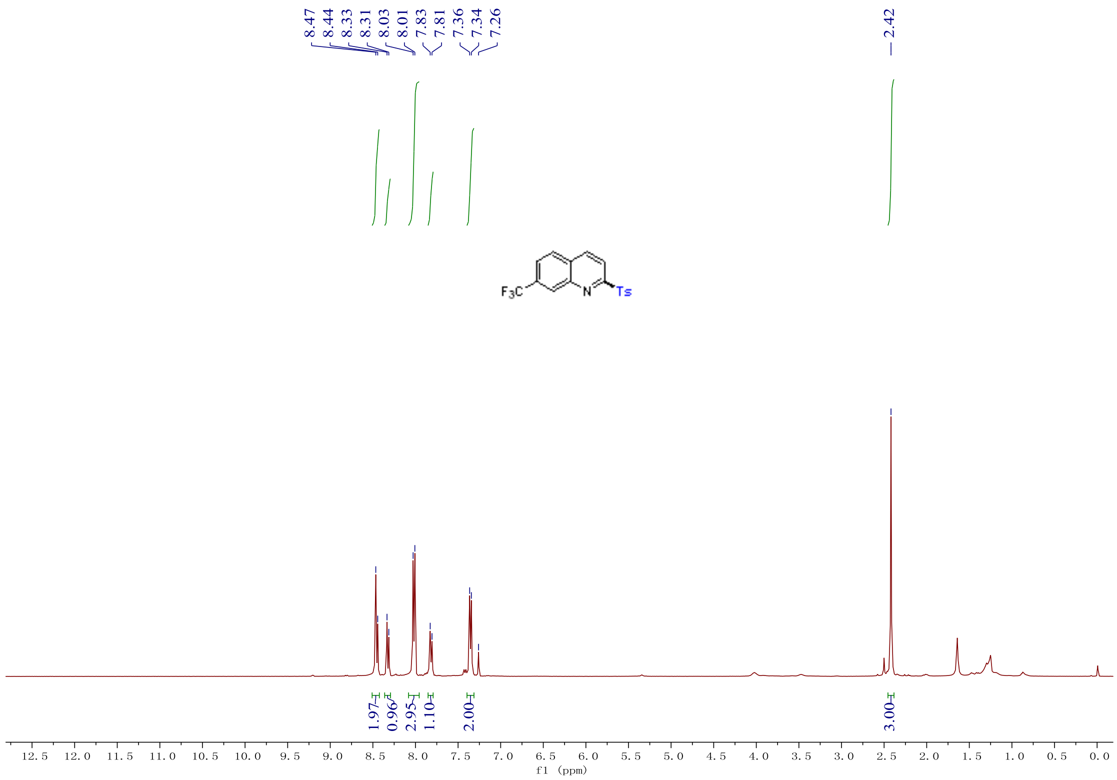

^{1}H NMR ($CDCl_3$, 400 M) spectra of **3na**

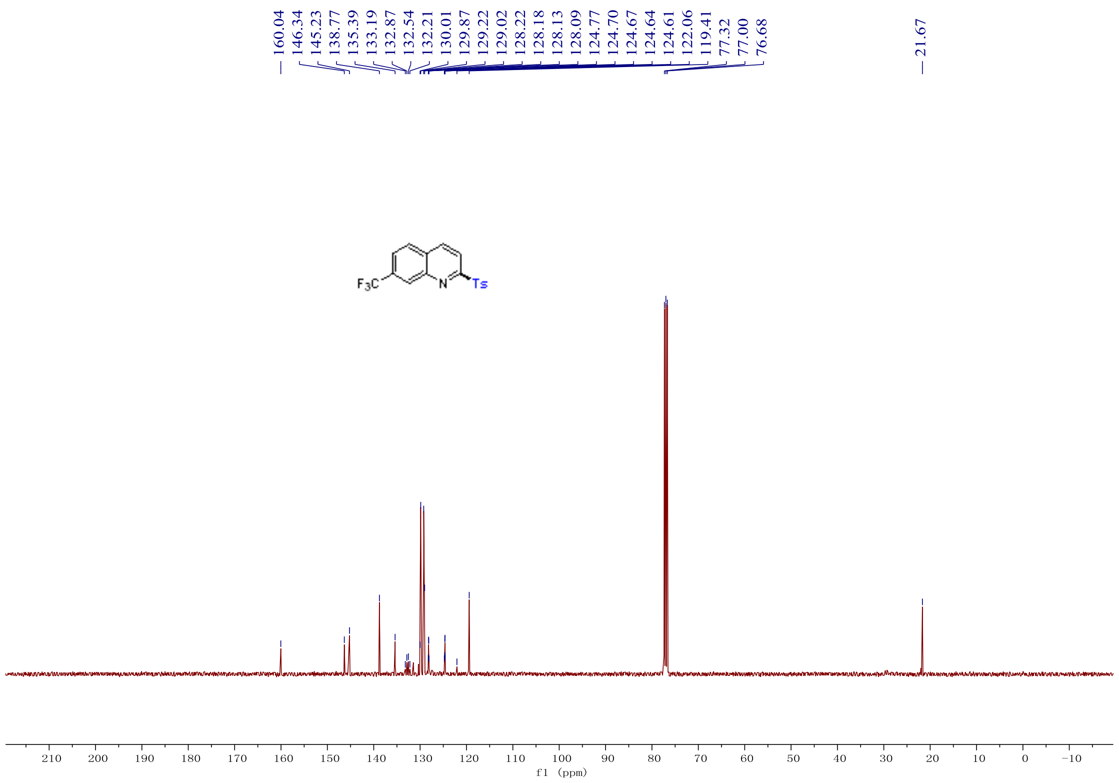

^{13}C NMR ($CDCl_3$, 100 M) spectra of **3na**

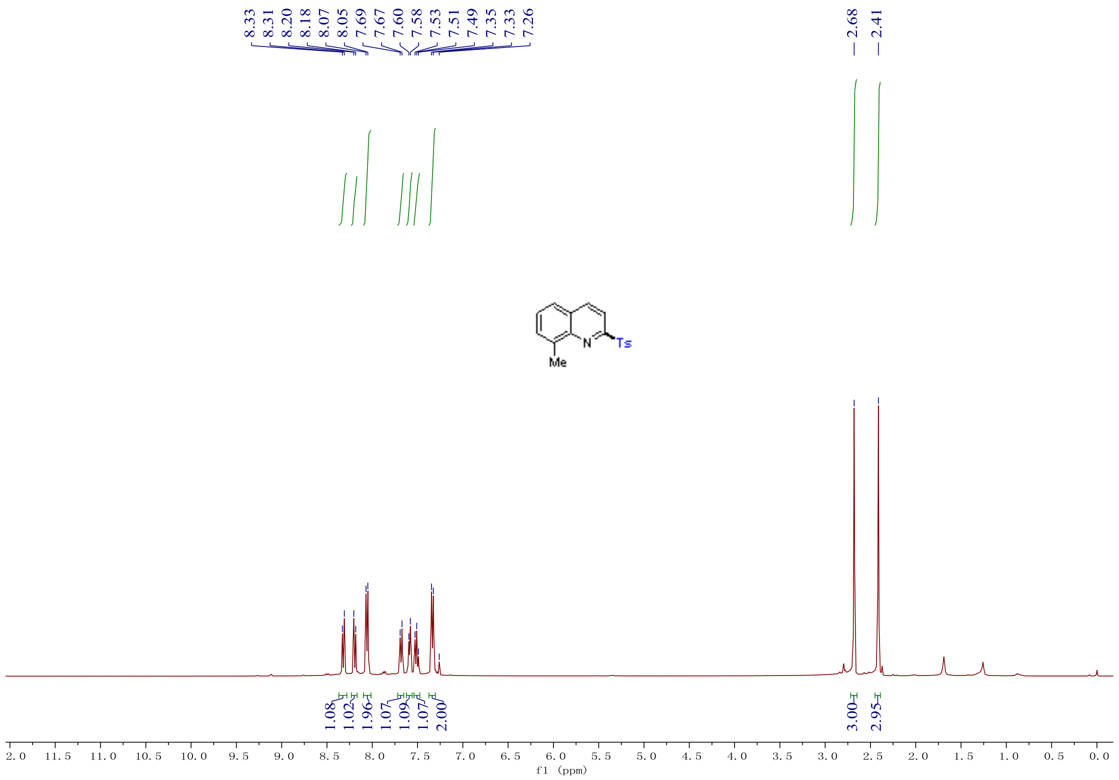

^{1}H NMR ($CDCl_3$, 400 M) spectra of **3oa**

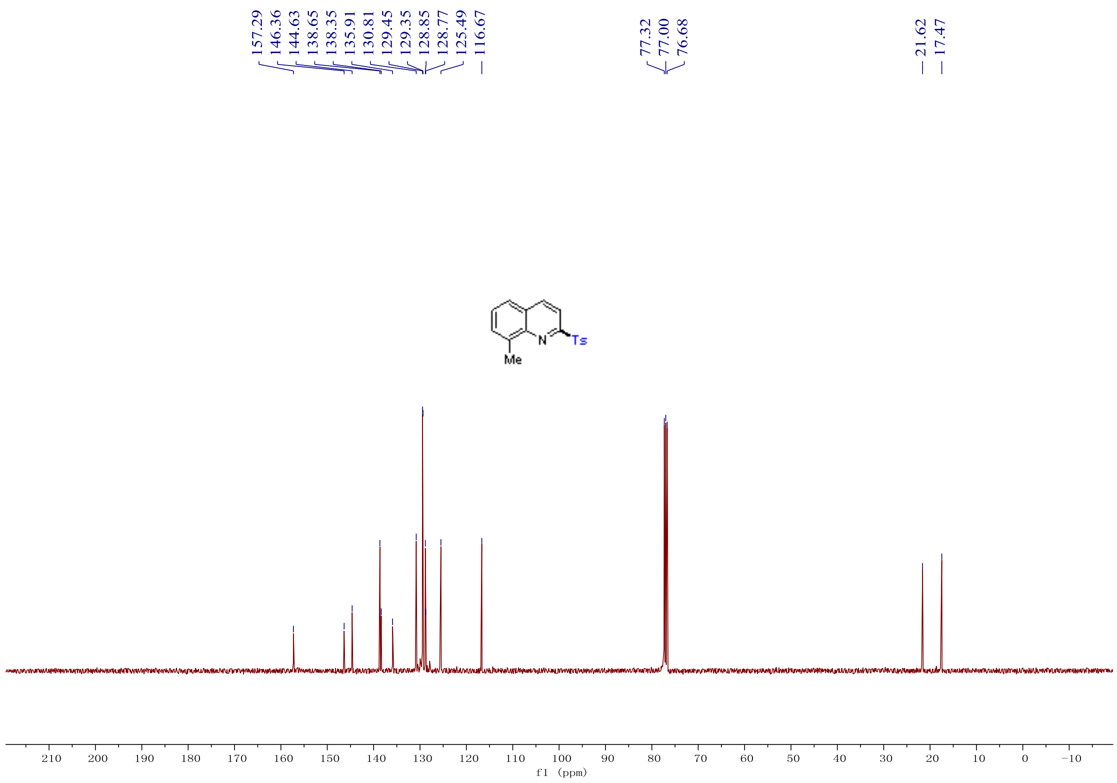

^{13}C NMR ($CDCl_3$, 100 M) spectra of **3oa**

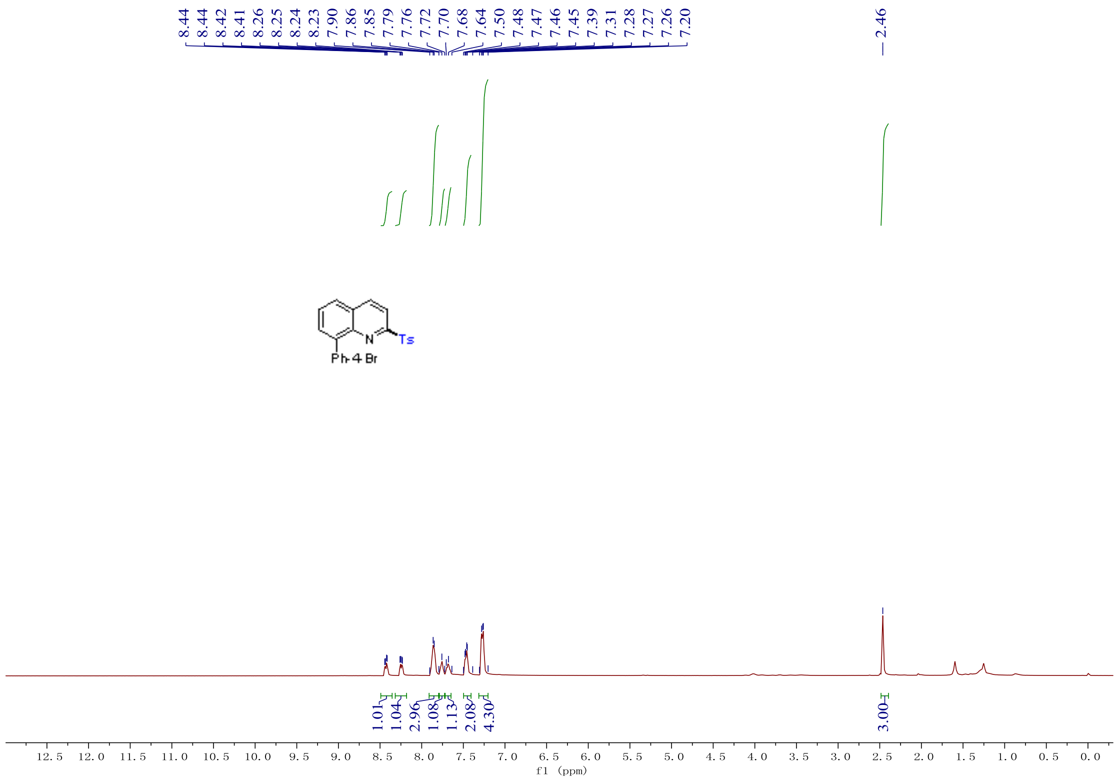

^{1}H NMR ($CDCl_3$, 400 M) spectra of **3pa**

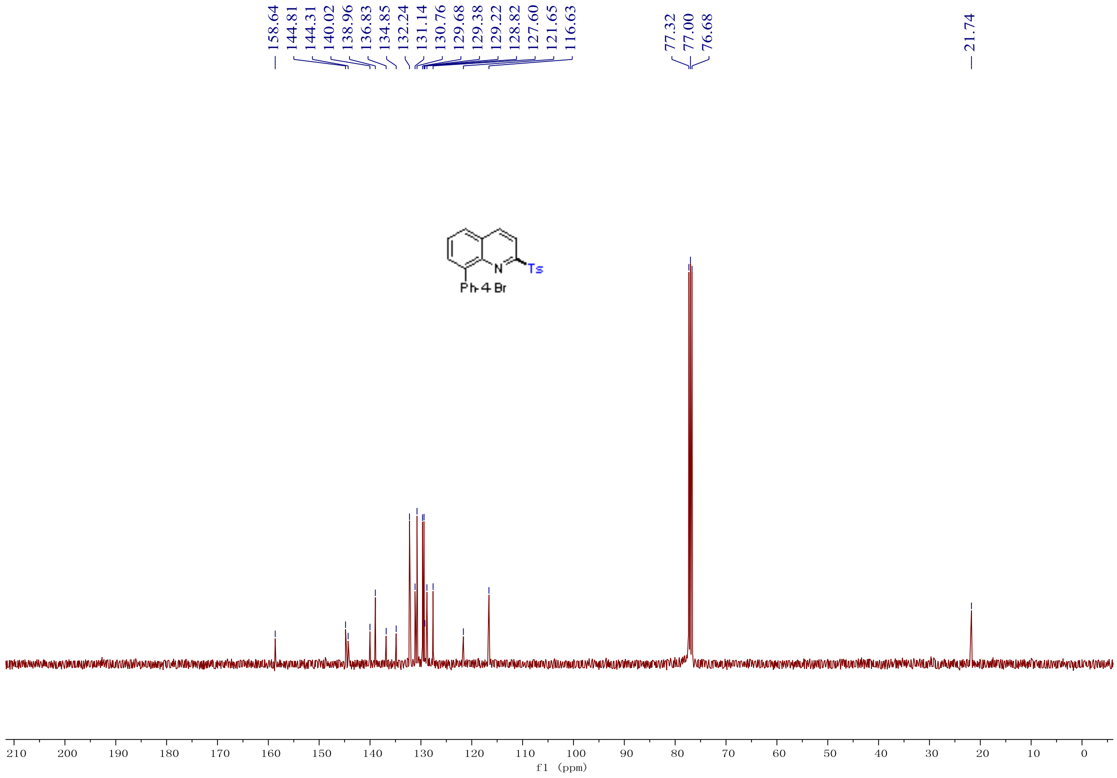

^{13}C NMR (CDCl$_3$, 100 M) spectra of **3pa**

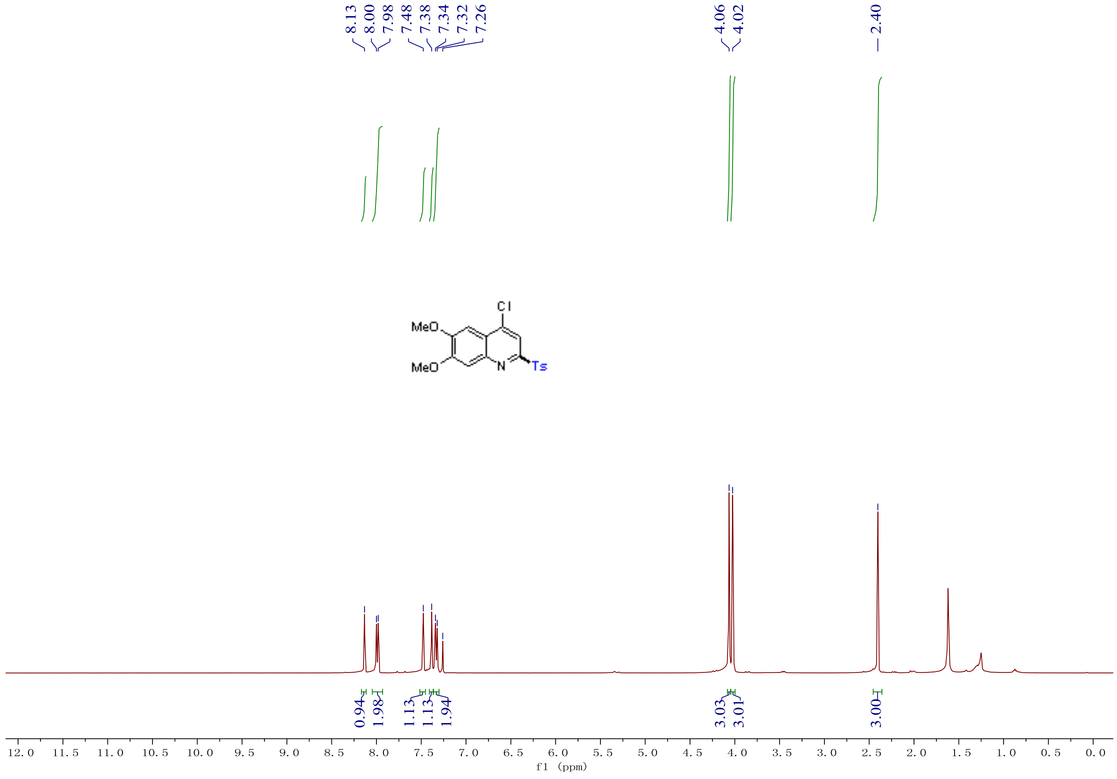

^{1}H NMR (CDCl$_3$, 400 M) spectra of **3qa**

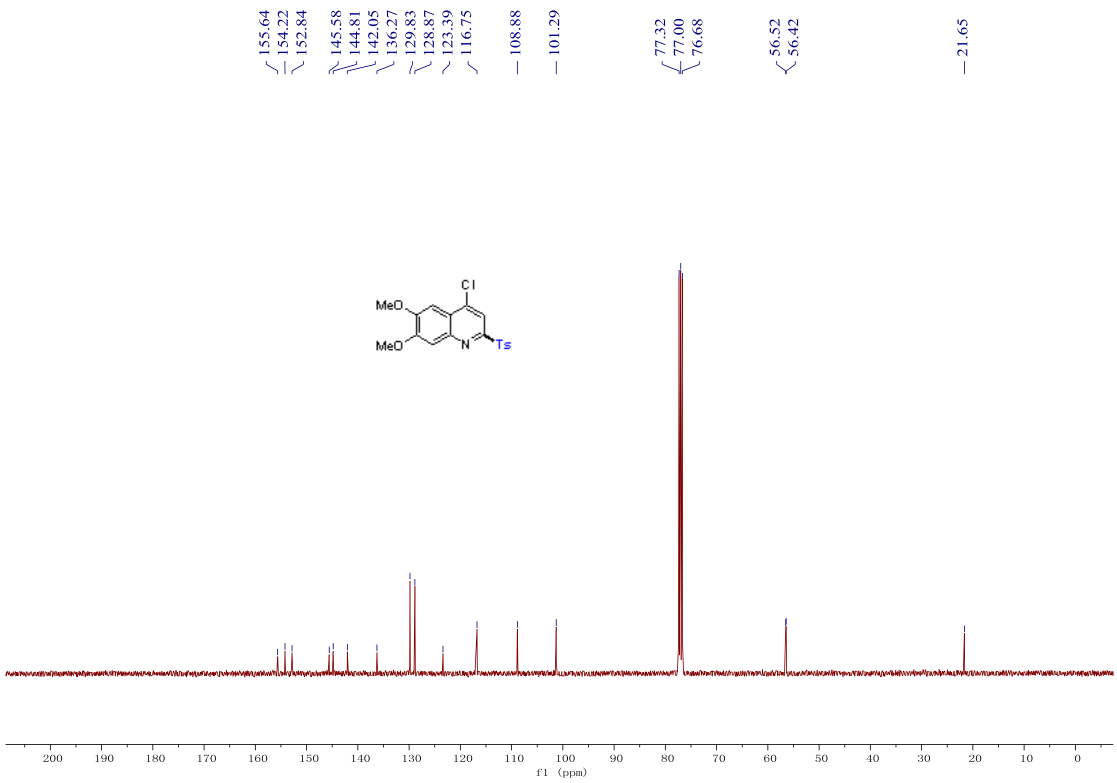

^{13}C NMR ($CDCl_3$, 100 M) spectra of **3qa**

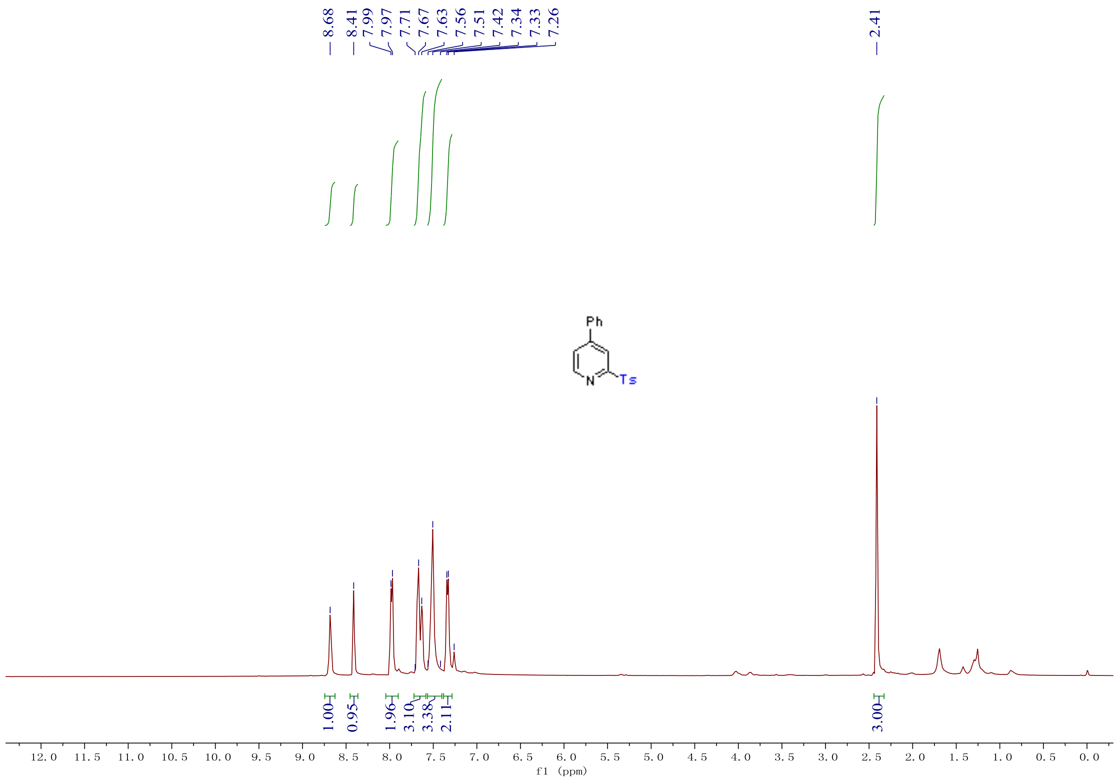

^{1}H NMR ($CDCl_3$, 400 M) spectra of **3xa**

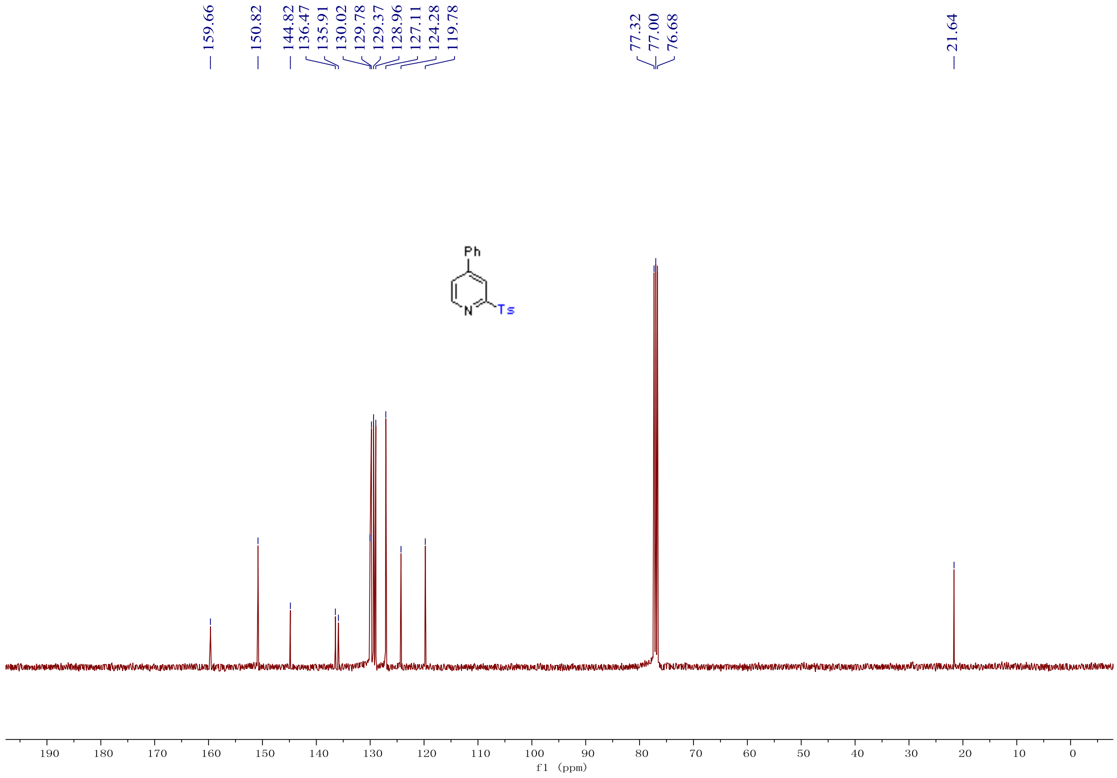

^{13}C NMR ($CDCl_3$, 100 M) spectra of **3xa**

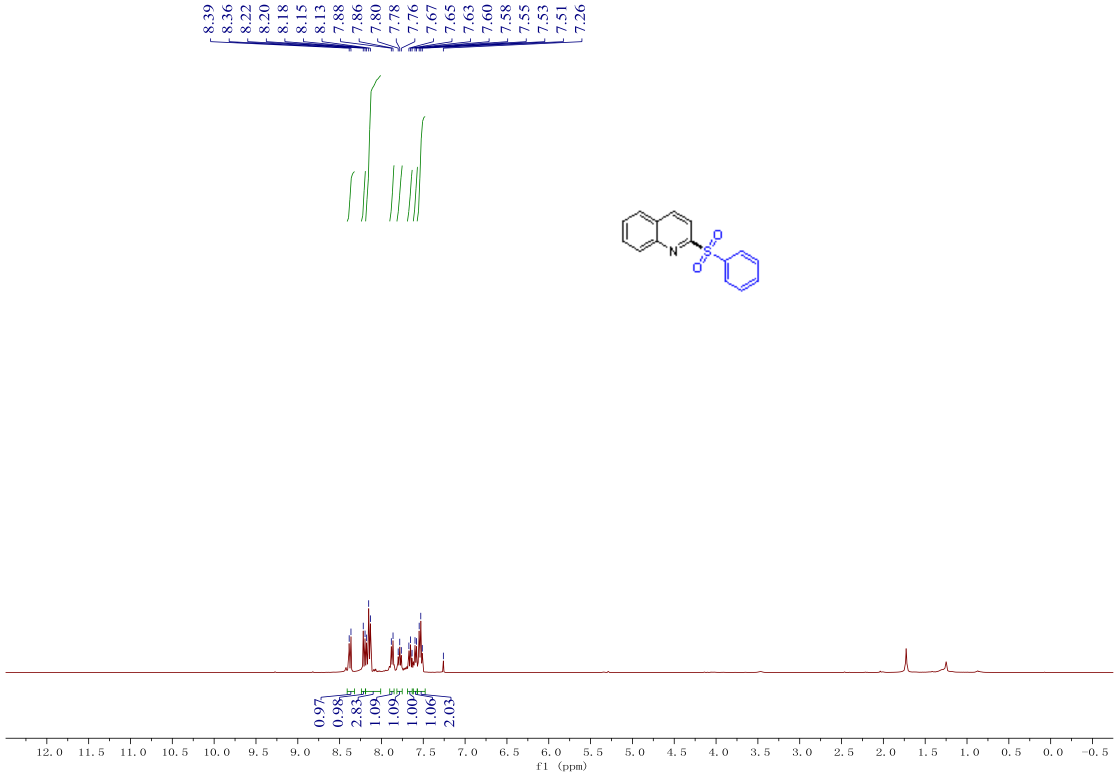

^{1}H NMR ($CDCl_3$, 400 M) spectra of **3ab**

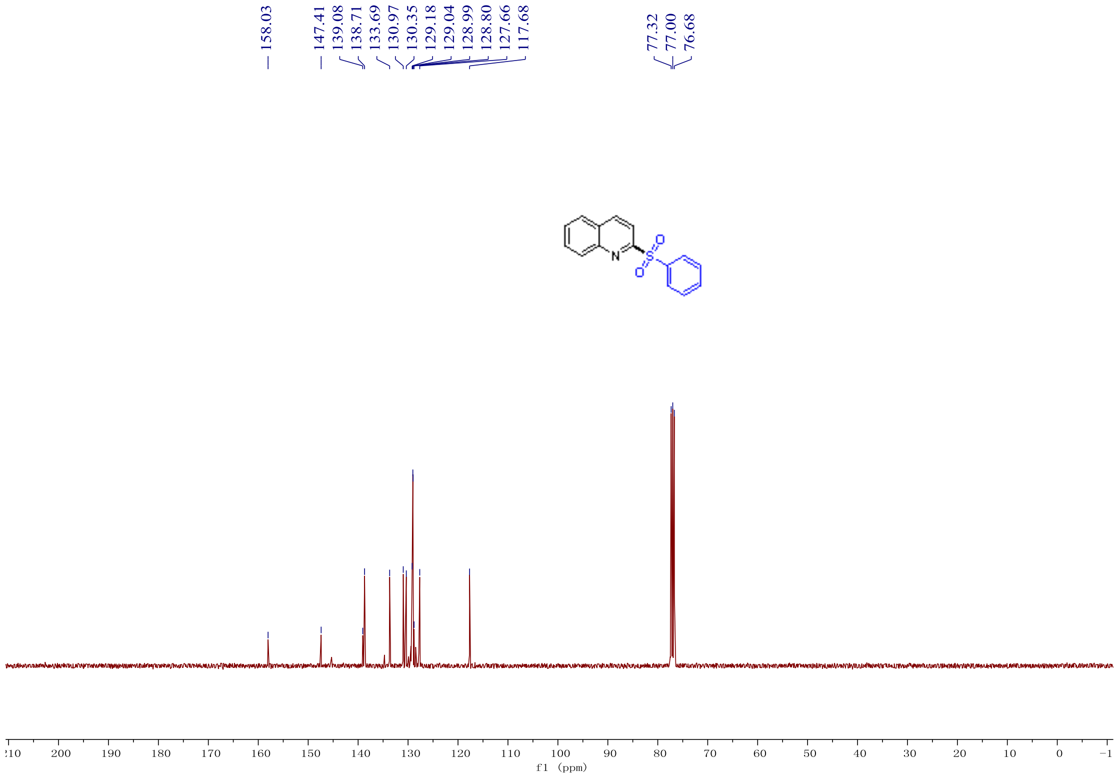

^{13}C NMR ($CDCl_3$, 100 M) spectra of **3ab**

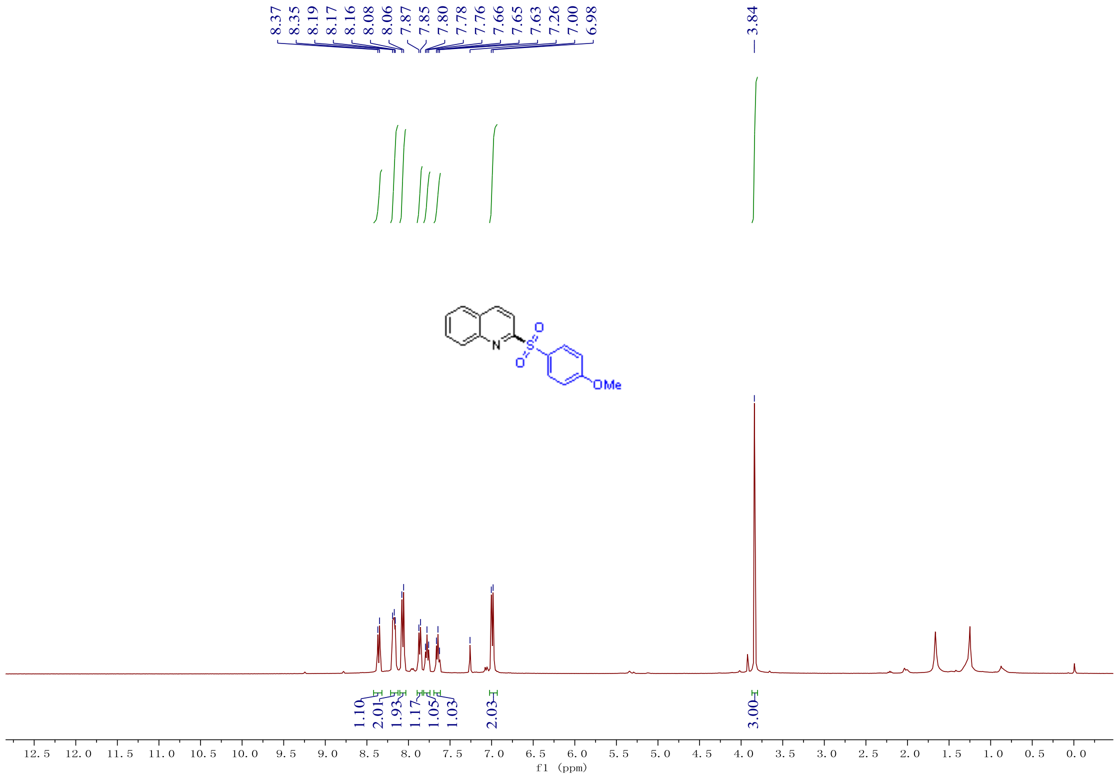

^{1}H NMR ($CDCl_3$, 400 M) spectra of **3ac**

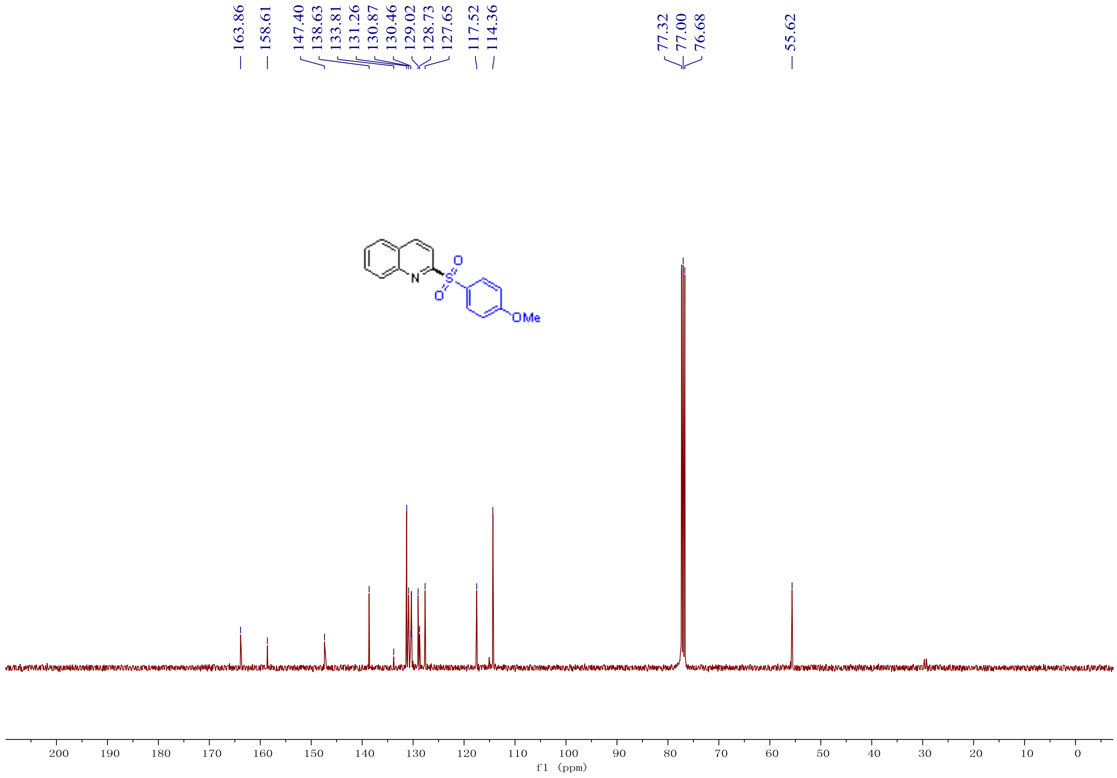

^{13}C NMR ($CDCl_3$, 400 M) spectra of **3ac**

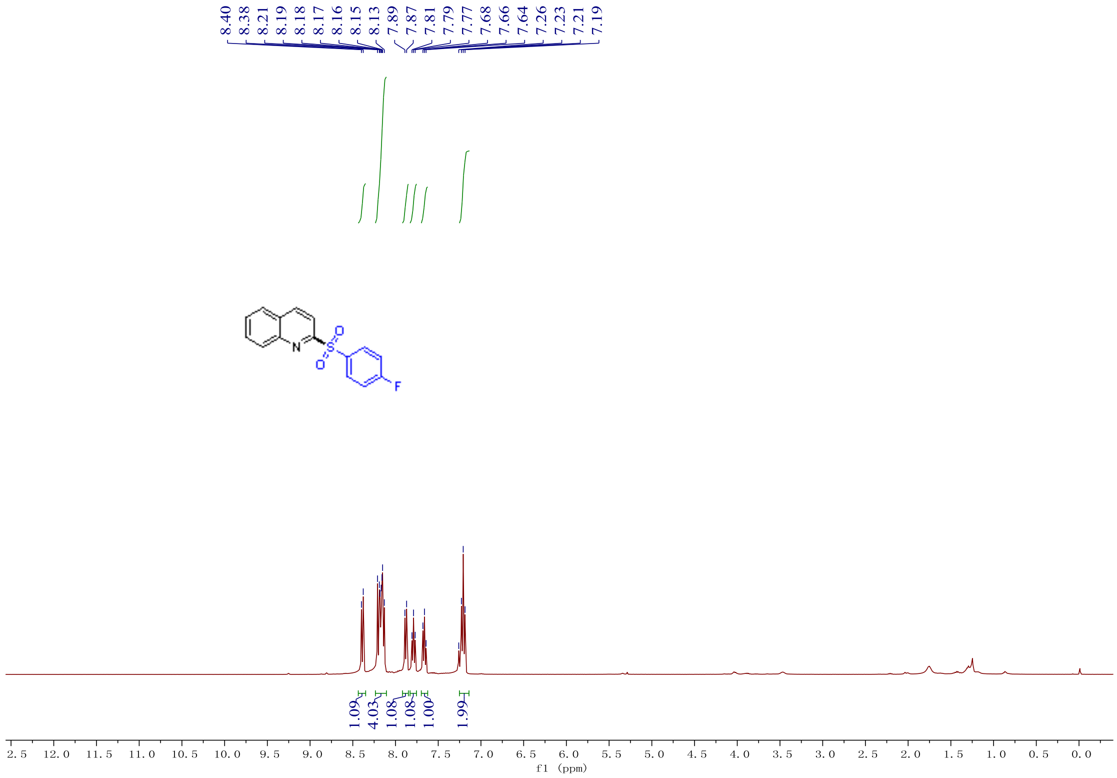

^{1}H NMR ($CDCl_3$, 400 M) spectra of **3ad**

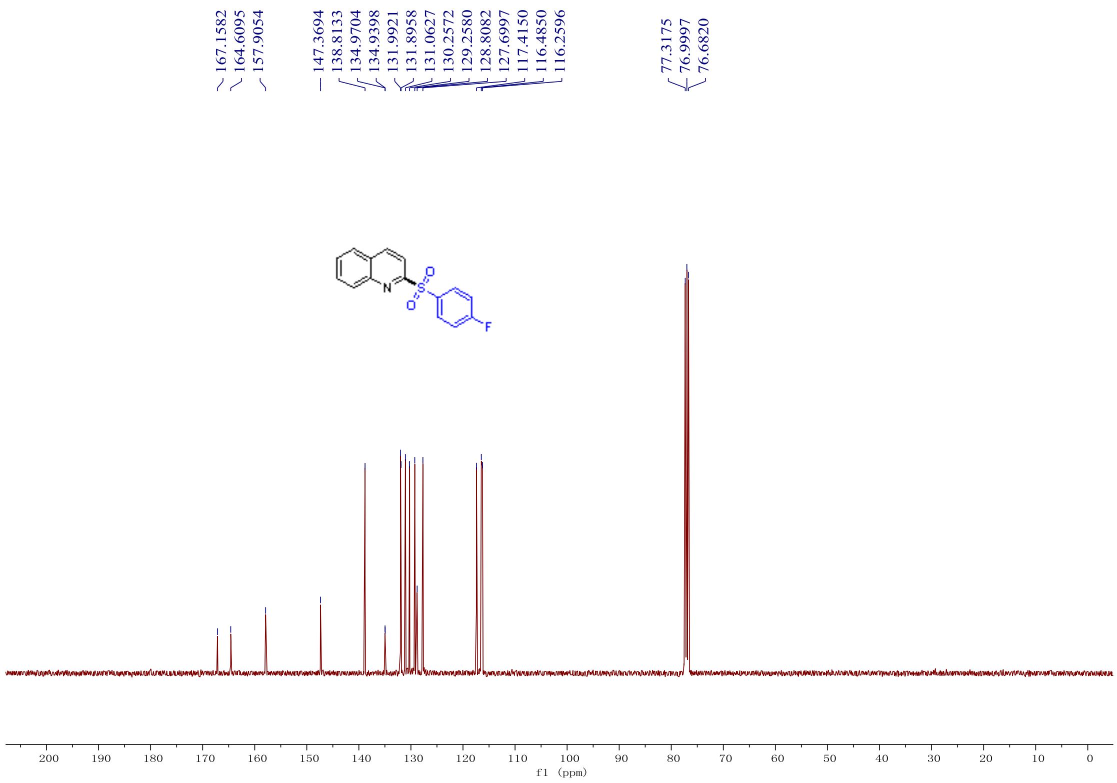

^{13}C NMR ($CDCl_3$, 100 M) spectra of **3ad**

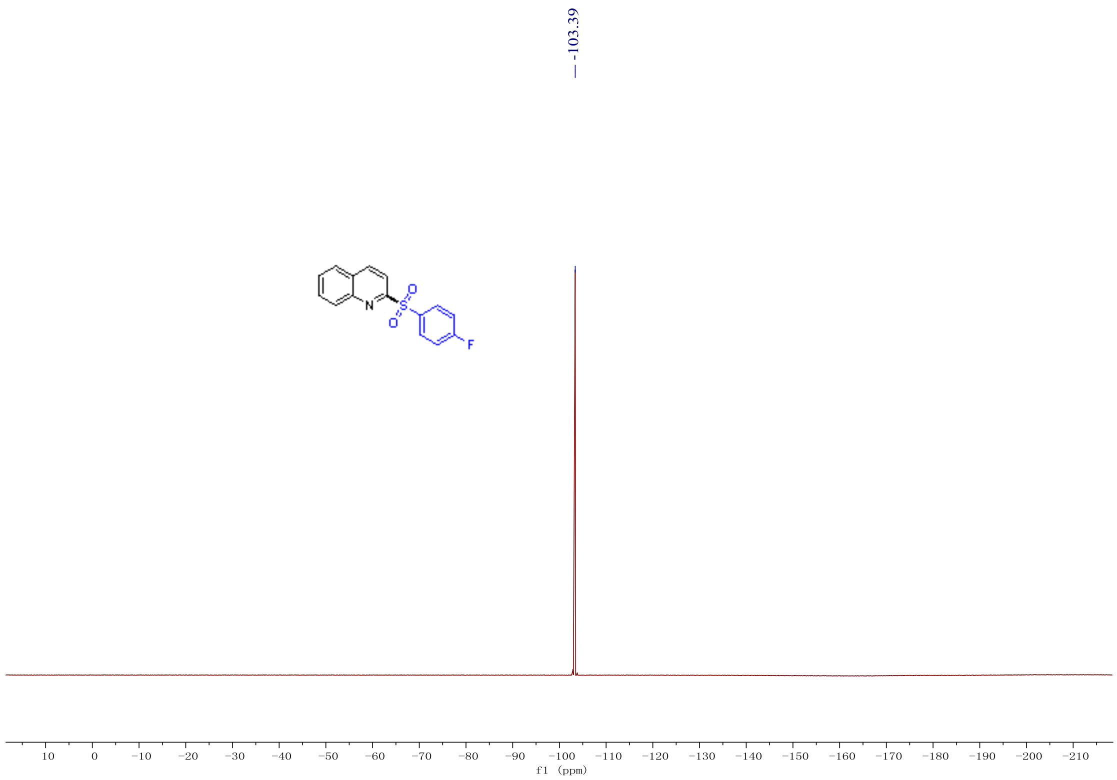

^{19}F NMR ($CDCl_3$, 376 M) spectra of **3ad**

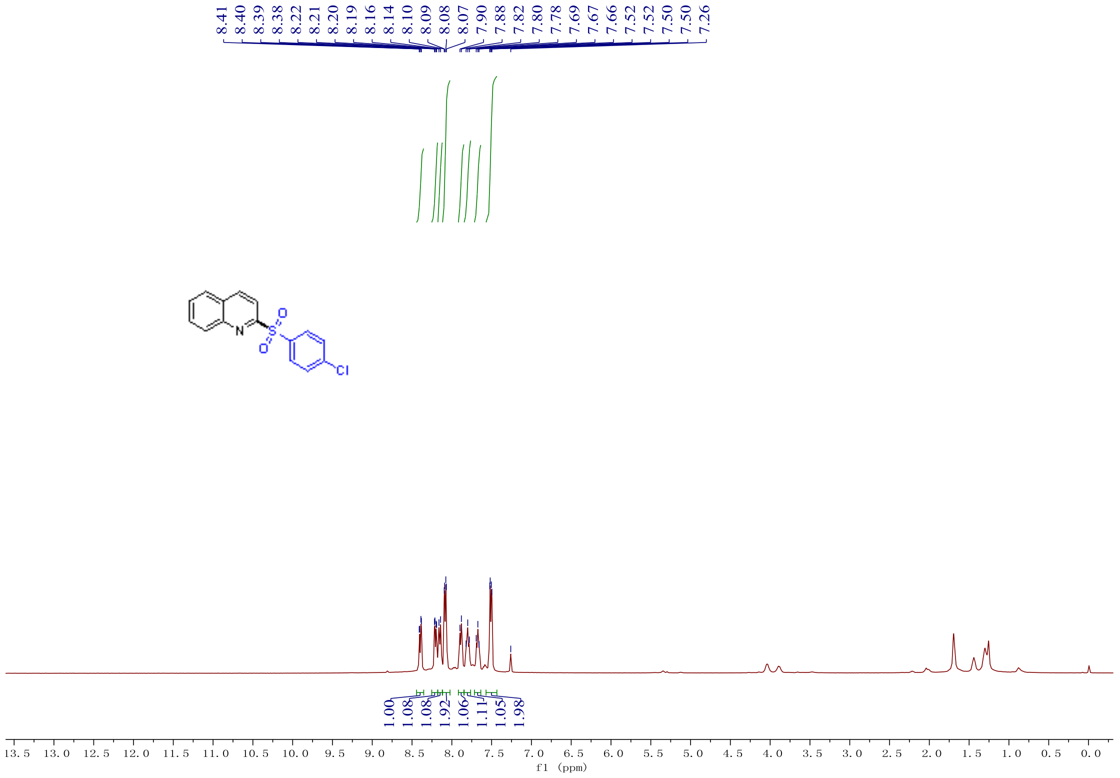

^{1}H NMR ($CDCl_3$, 400 M) spectra of **3ae**

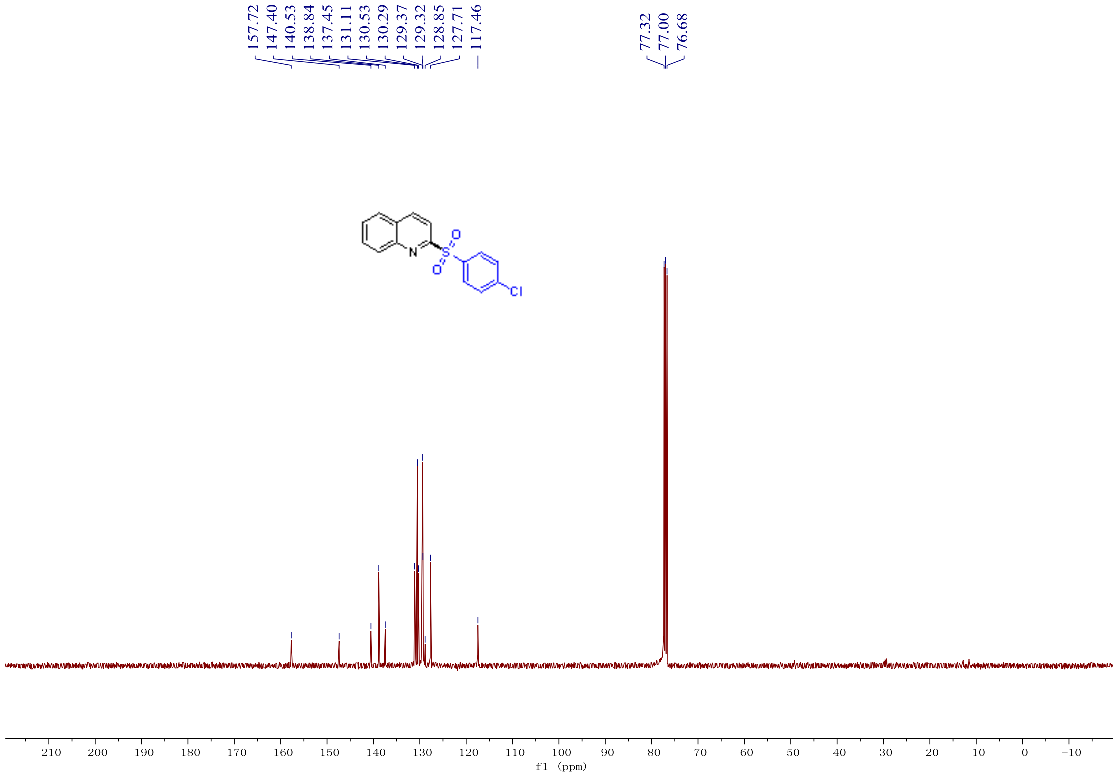

^{13}C NMR ($CDCl_3$, 100 M) spectra of **3ae**

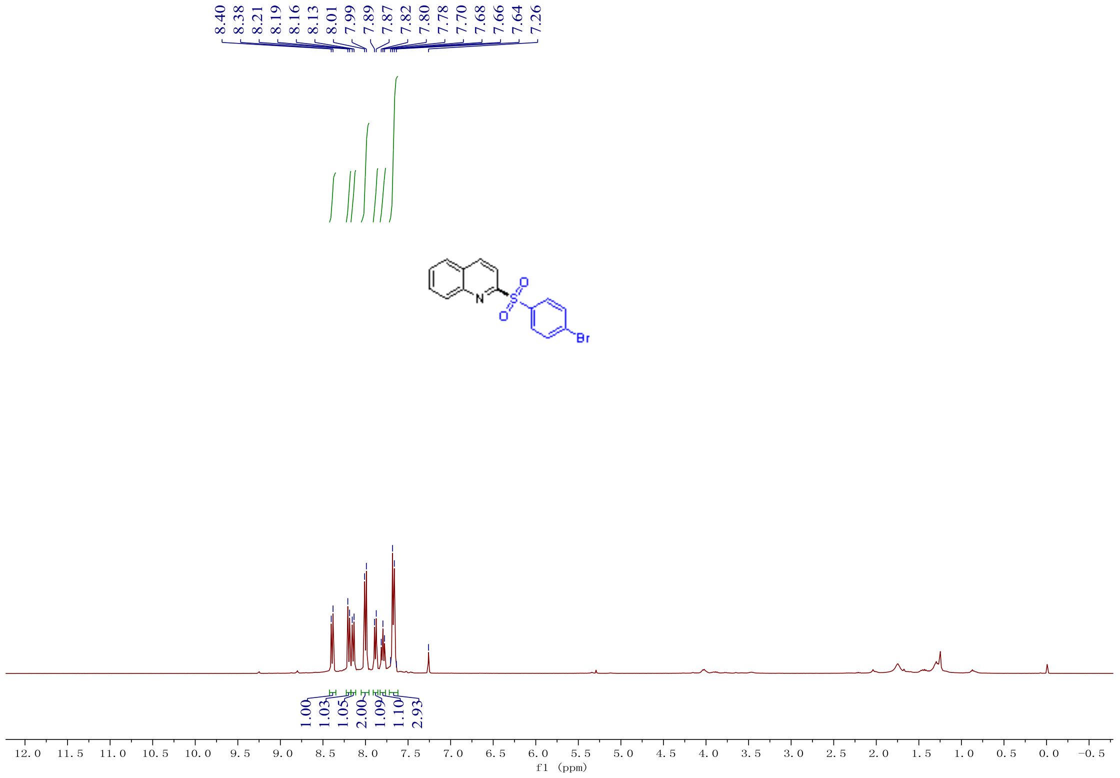

^{1}H NMR ($CDCl_3$, 400 M) spectra of **3af**

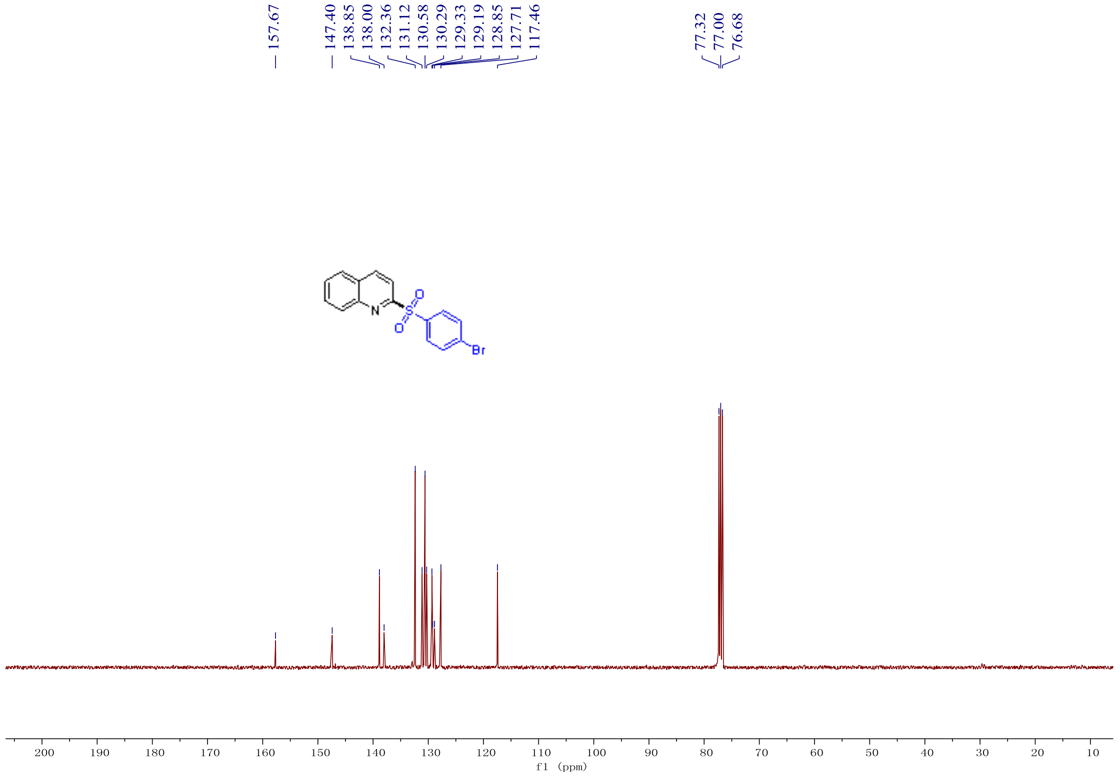

^{13}C NMR ($CDCl_3$, 100 M) spectra of **3af**

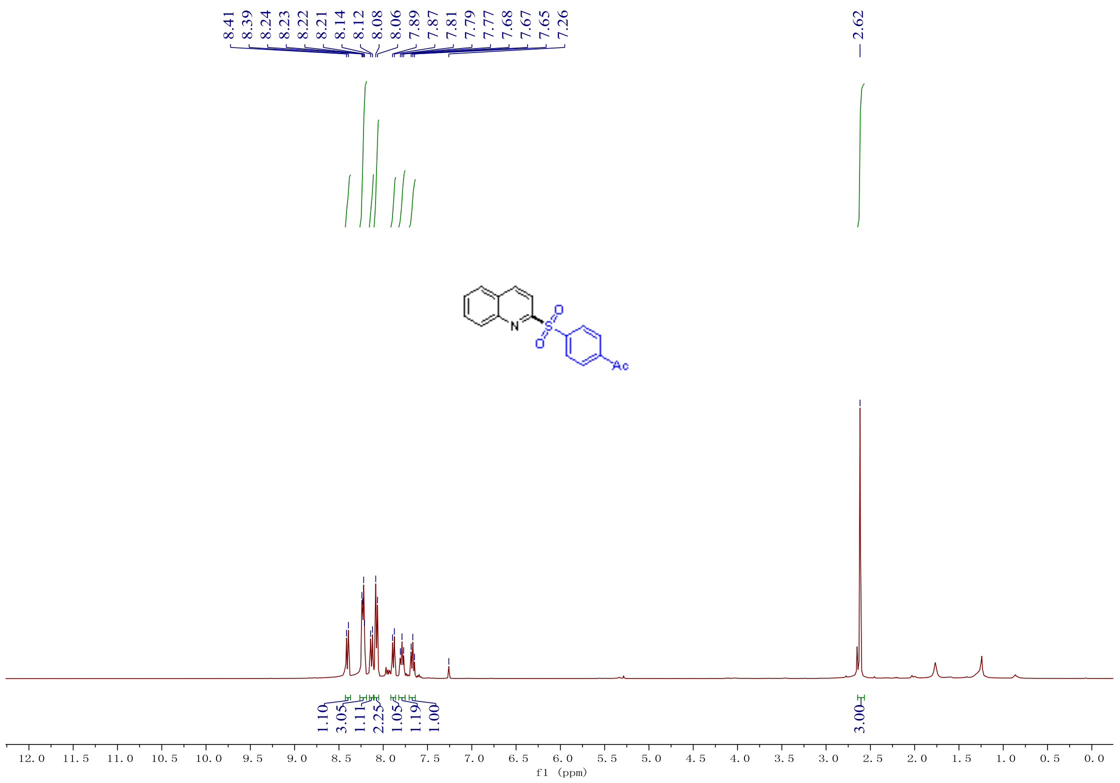

^{1}H NMR ($CDCl_3$, 400 M) spectra of **3ag**

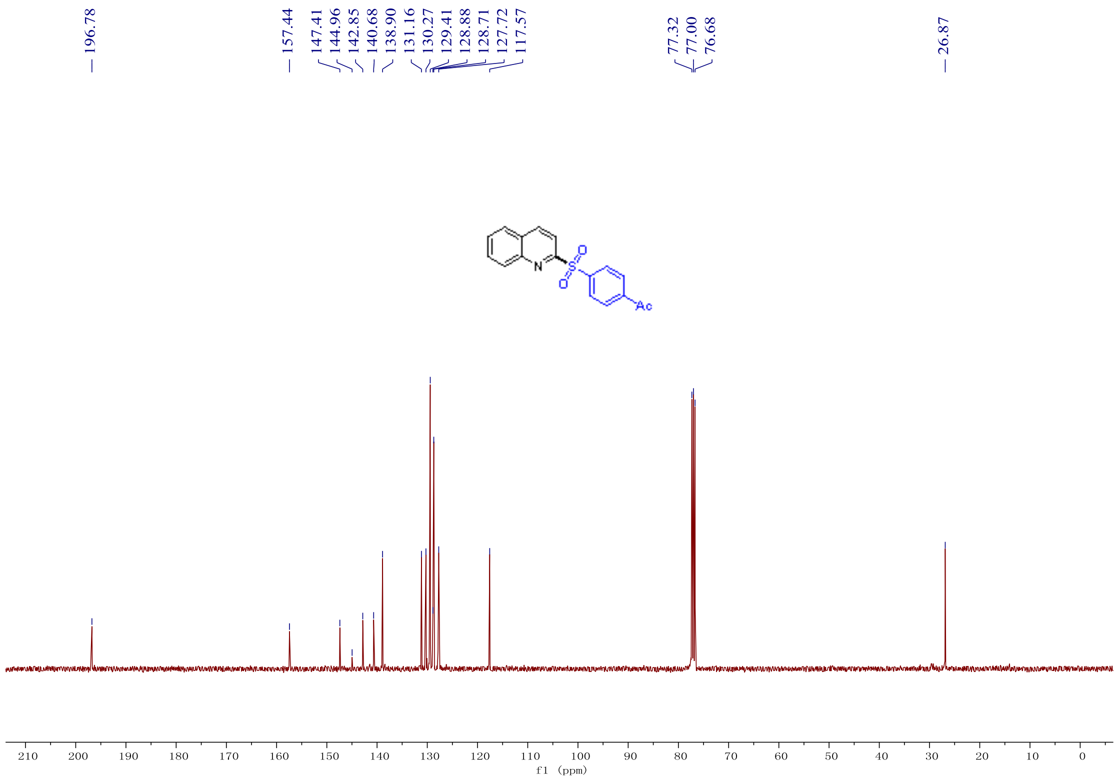

^{13}C NMR ($CDCl_3$, 100 M) spectra of **3ag**

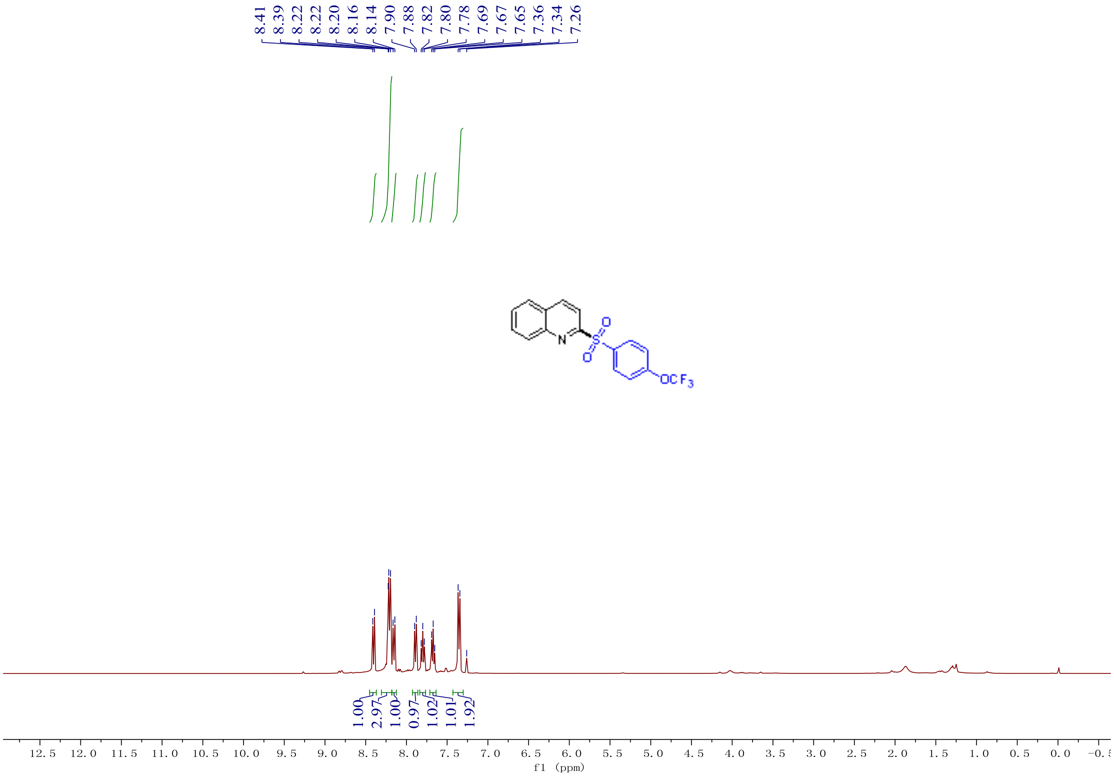

^{1}H NMR ($CDCl_3$, 400 M) spectra of **3ah**

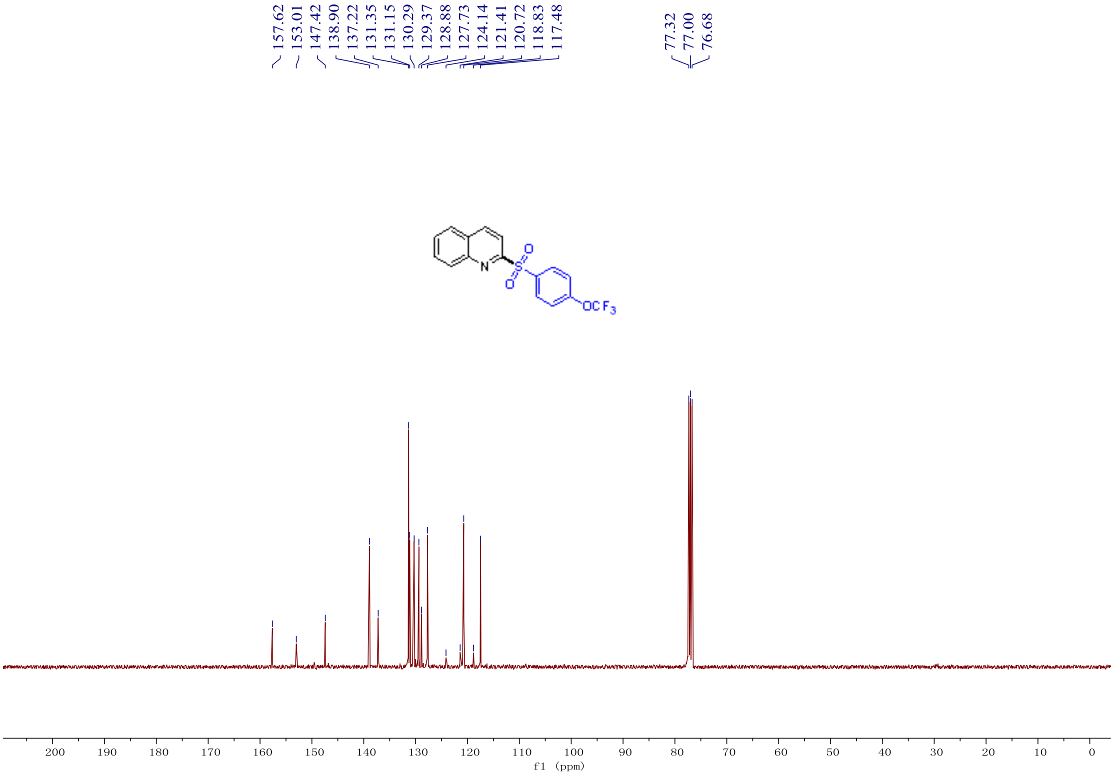

^{13}C NMR ($CDCl_3$, 100 M) spectra of **3ah**

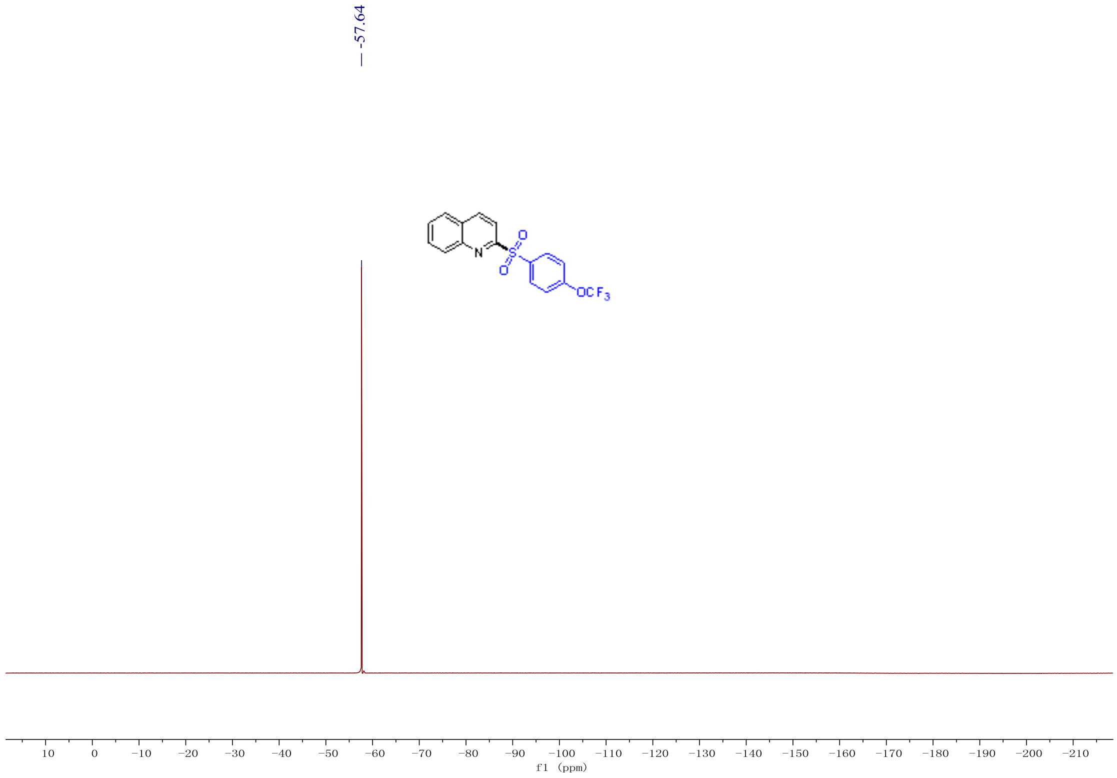

^{19}F NMR ($CDCl_3$, 100 M) spectra of **3ah**

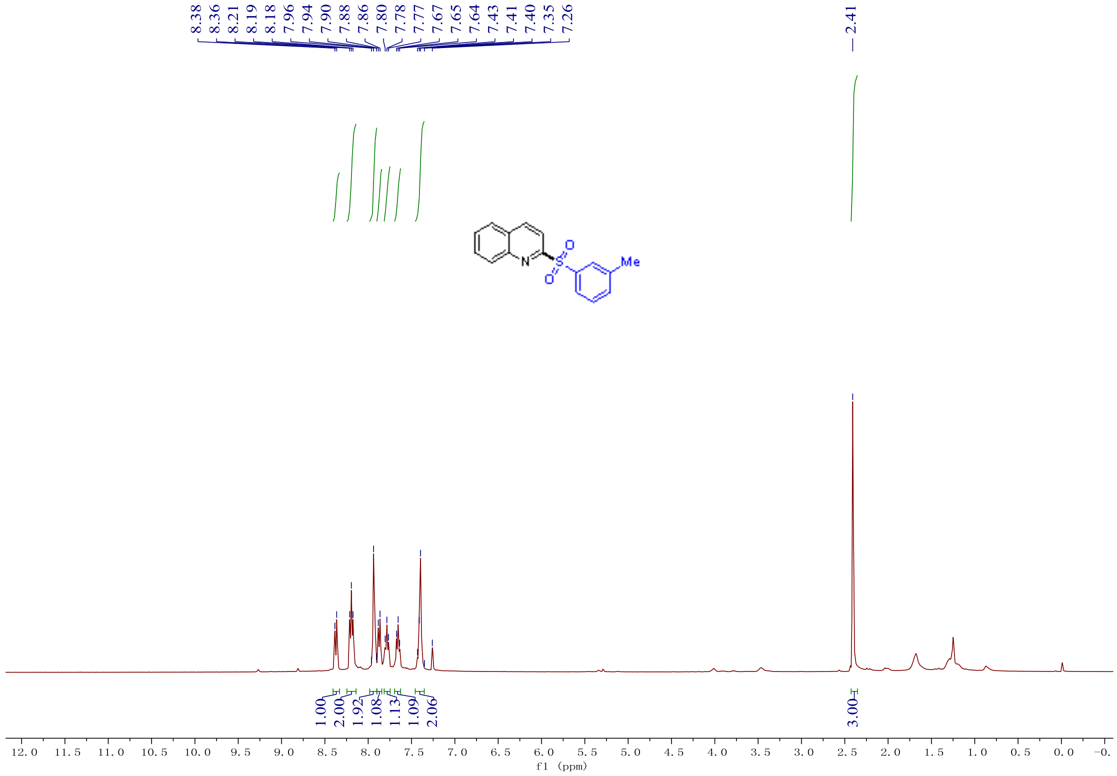

^{1}H NMR ($CDCl_3$, 400 M) spectra of **3ai**

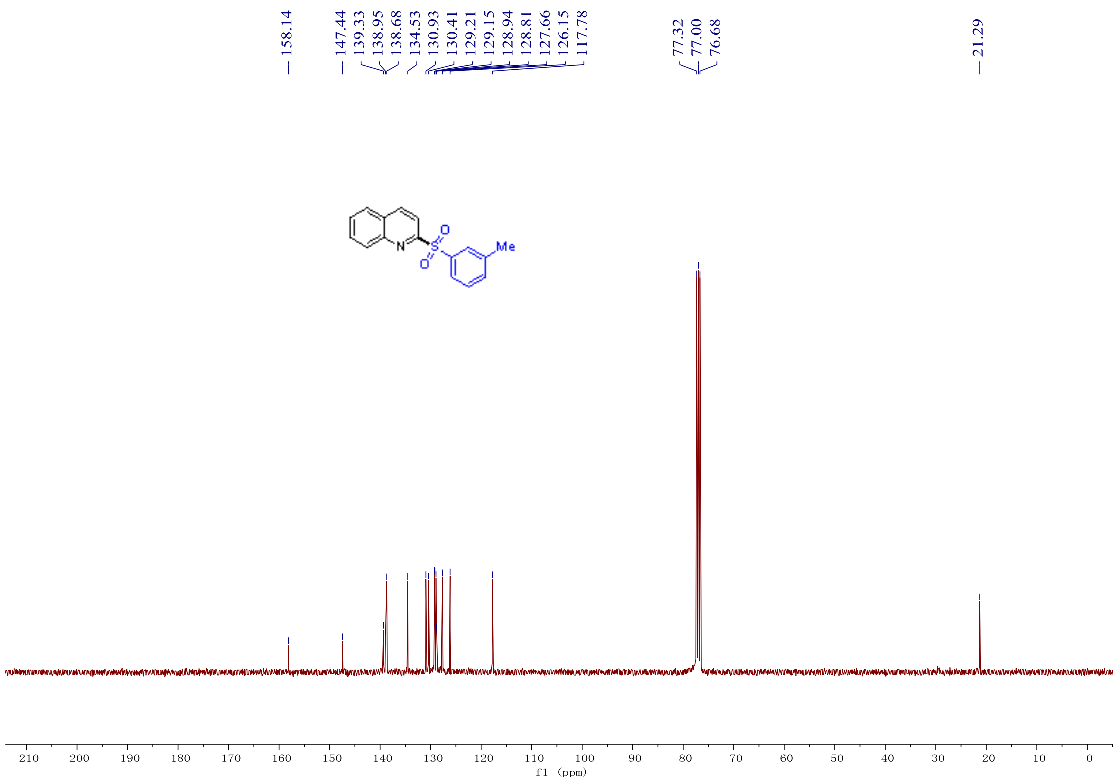

^{13}C NMR ($CDCl_3$, 100 M) spectra of **3ai**

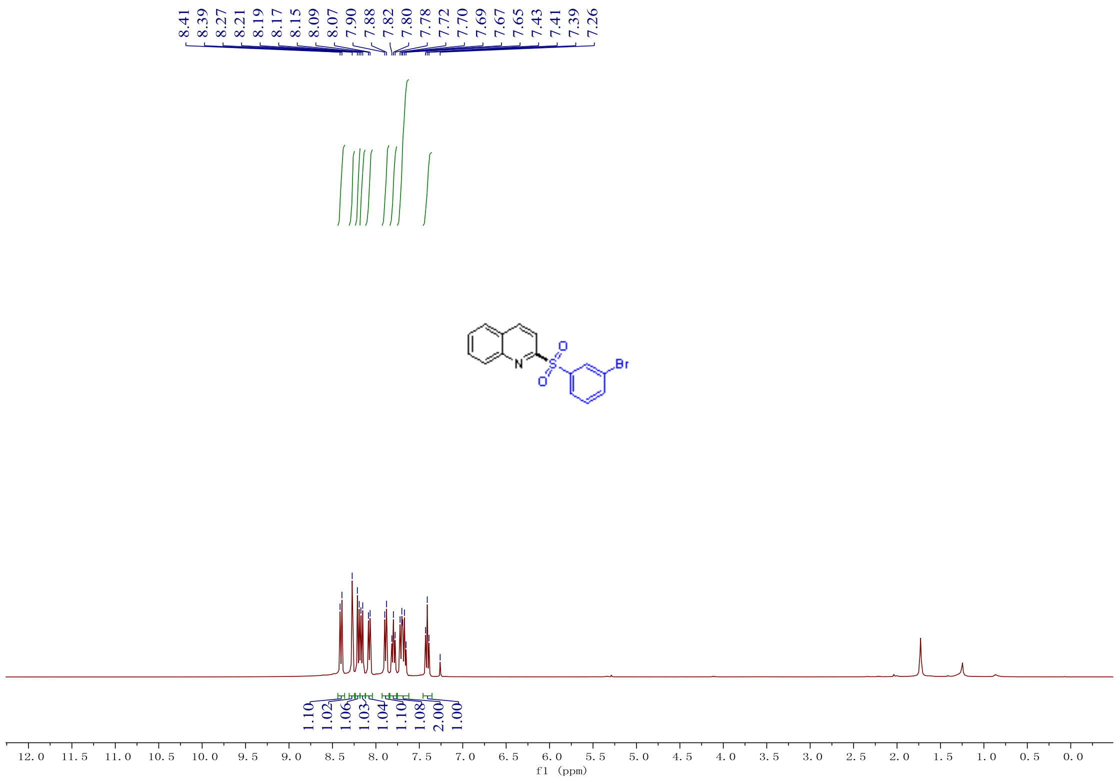

^{1}H NMR ($CDCl_3$, 400 M) spectra of **3aj**

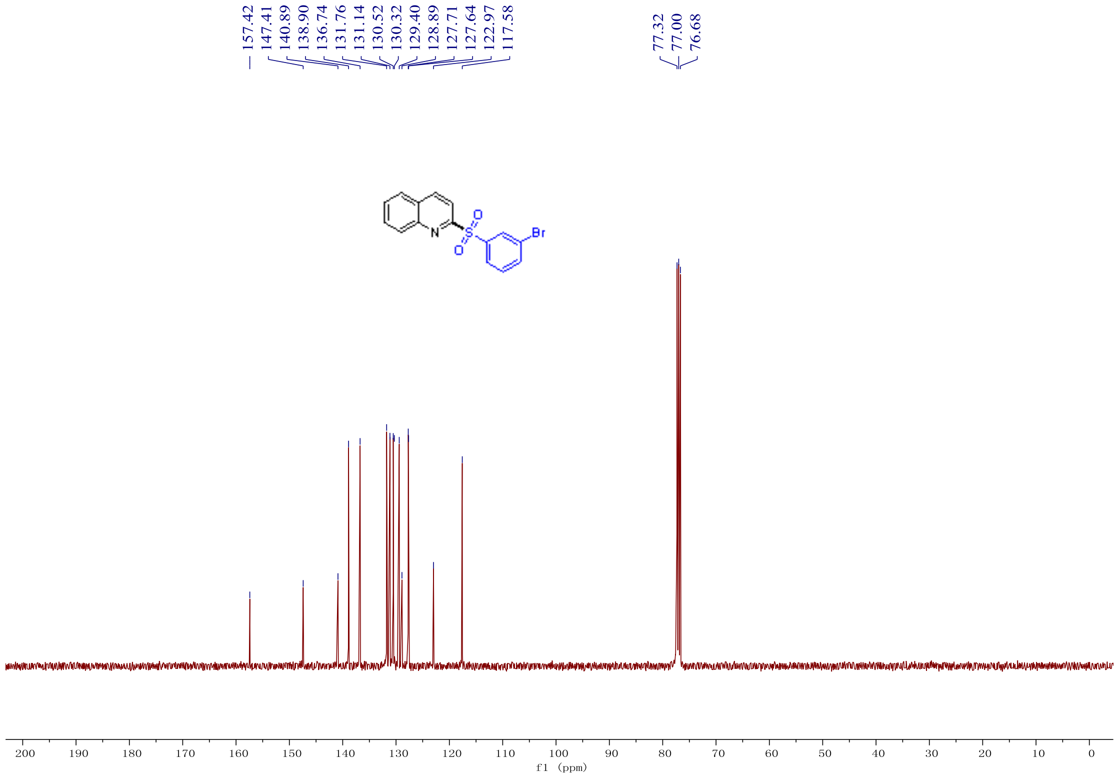

^{13}C NMR ($CDCl_3$, 100 M) spectra of **3aj**

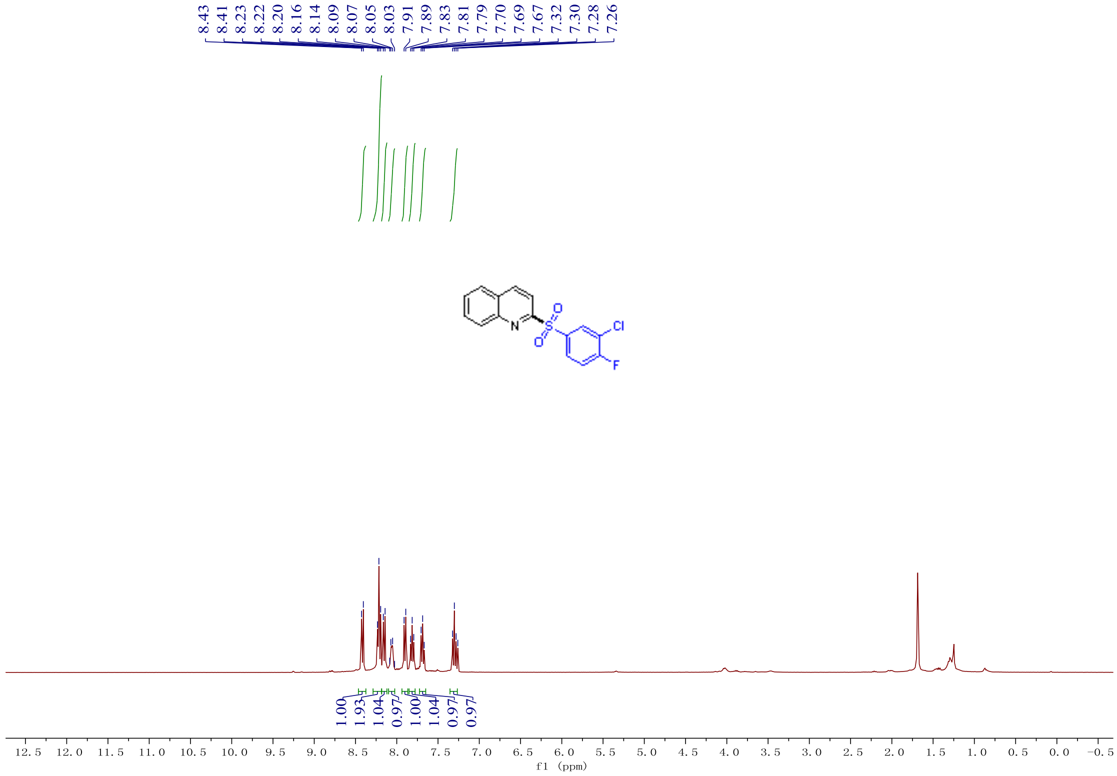

^{1}H NMR ($CDCl_3$, 400 M) spectra of **3ak**

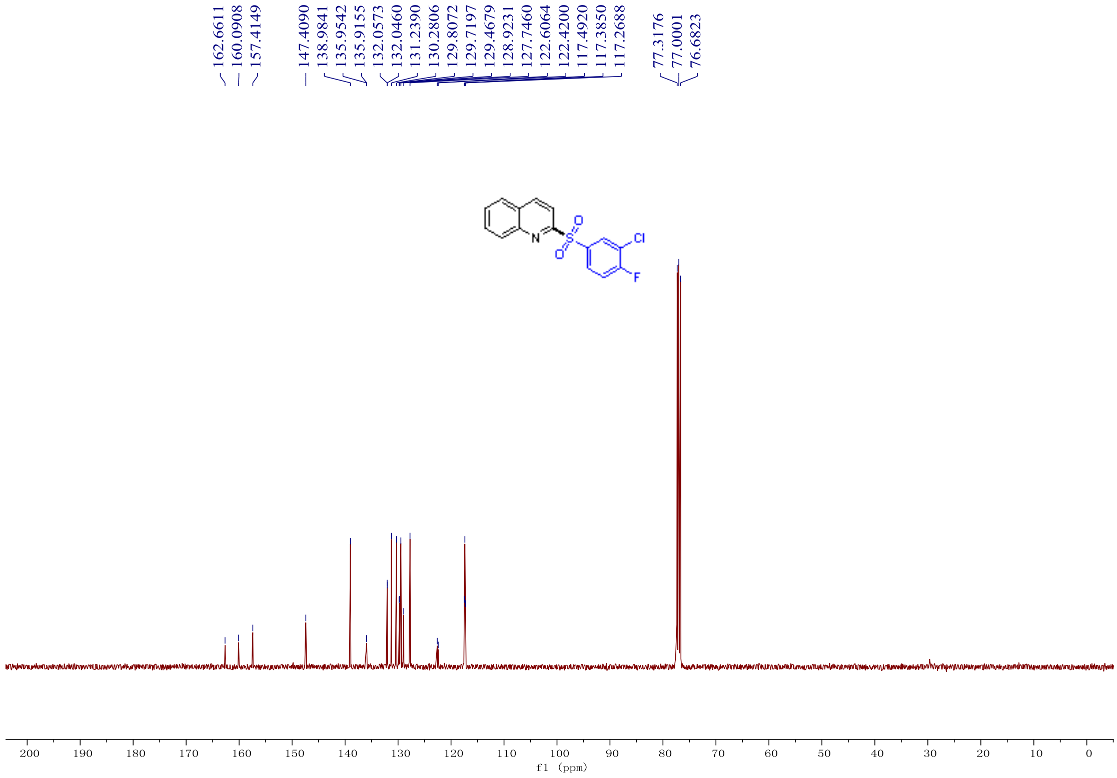

^{13}C NMR ($CDCl_3$, 100 M) spectra of **3ak**

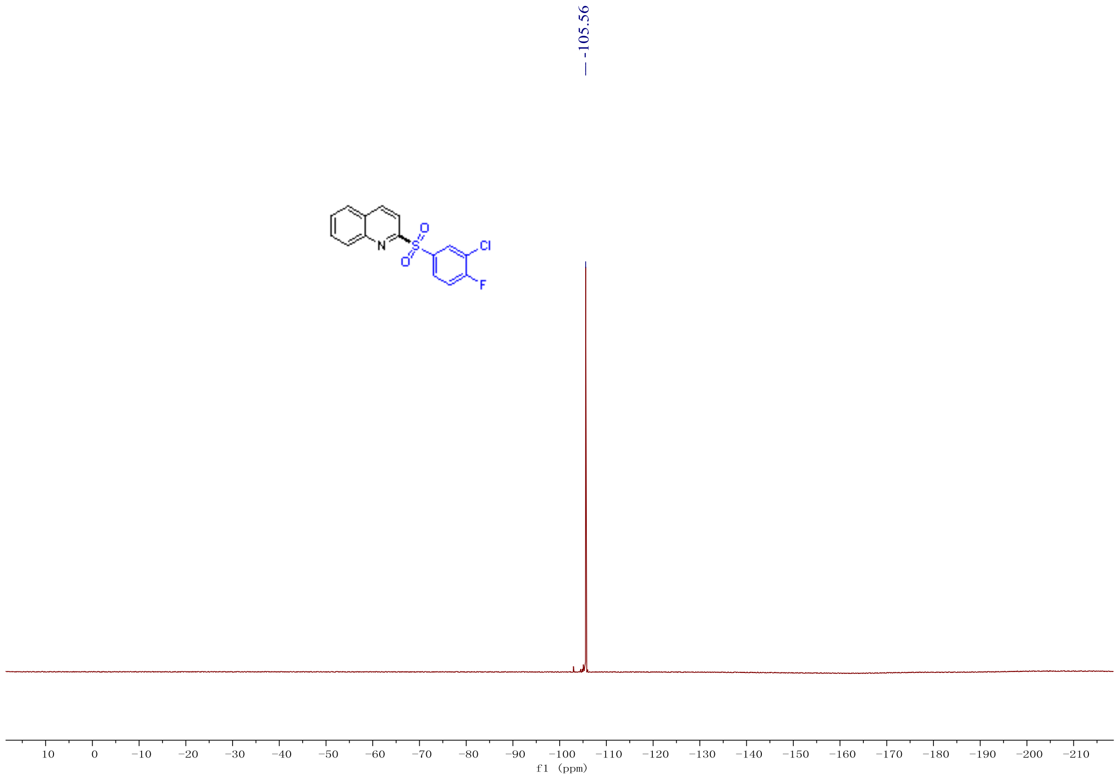

^{19}F NMR ($CDCl_3$, 376 M) spectra of **3ak**

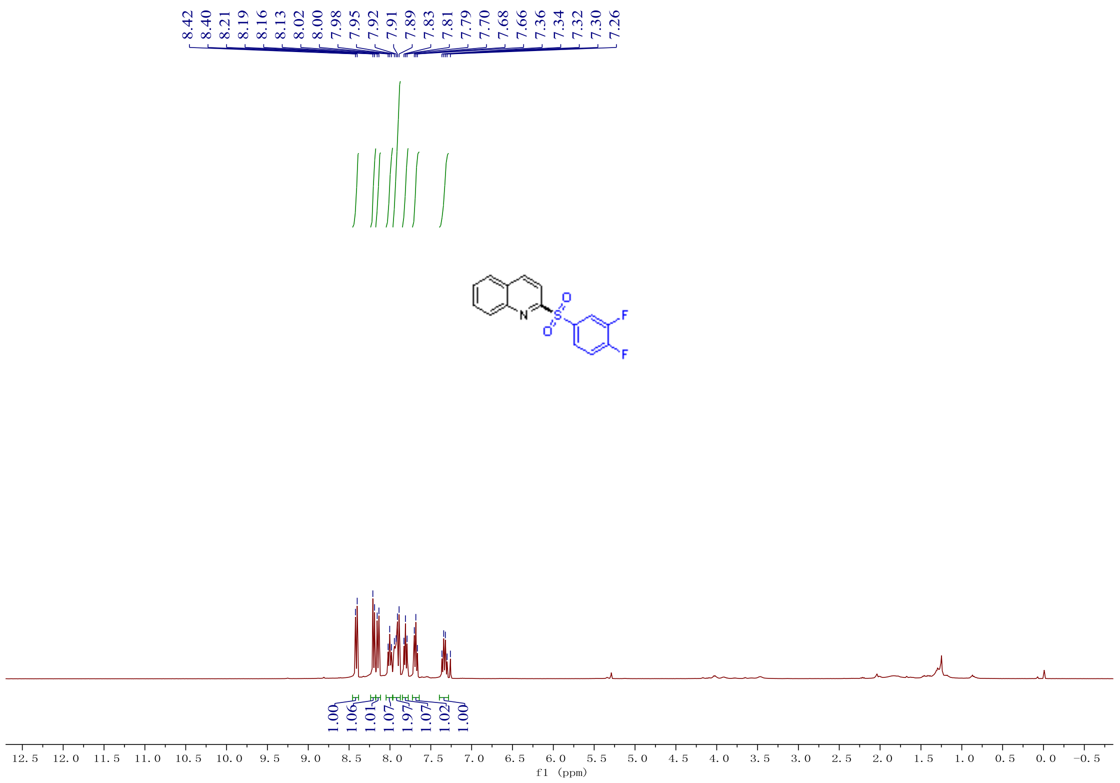

^{1}H NMR ($CDCl_3$, 400 M) spectra of **3al**

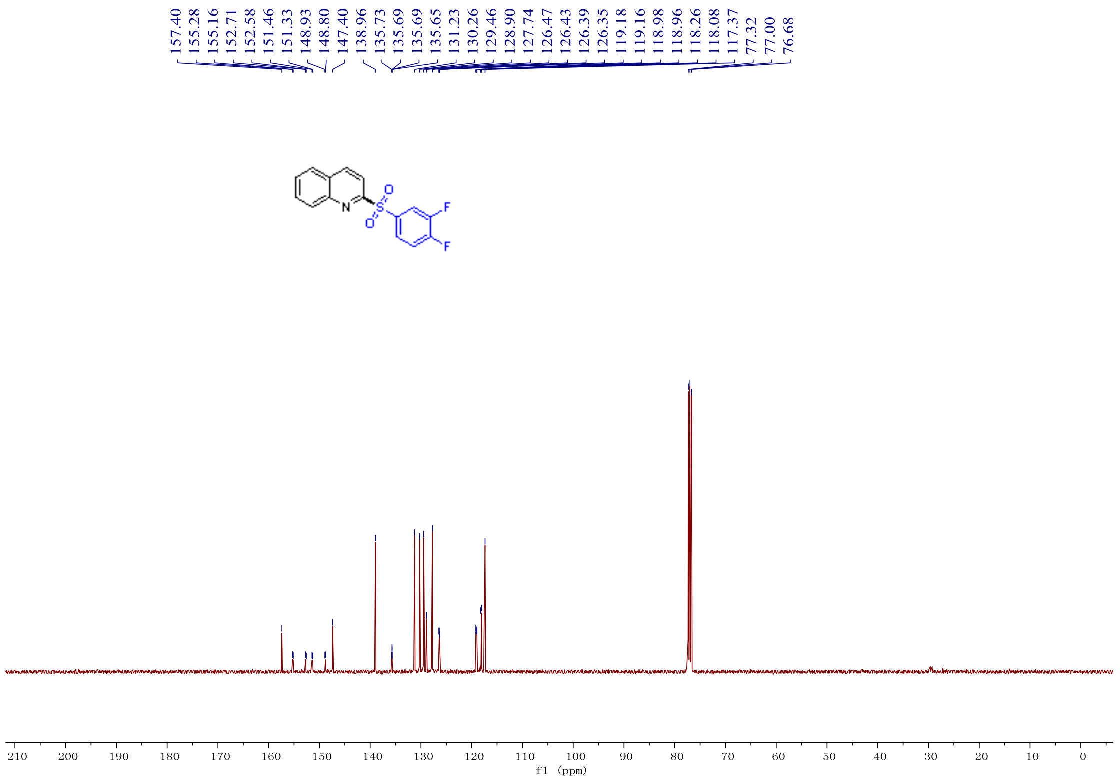

^{13}C NMR ($CDCl_3$, 100 M) spectra of **3al**

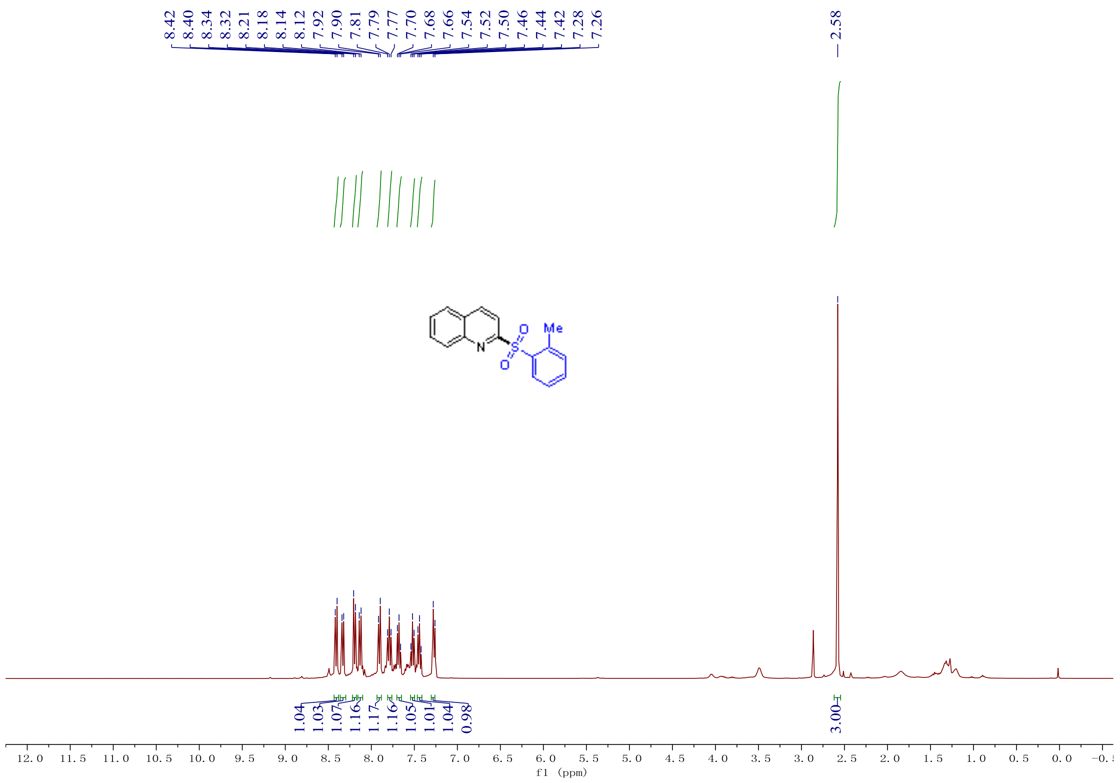

^{1}H NMR ($CDCl_3$, 400 M) spectra of **3am**

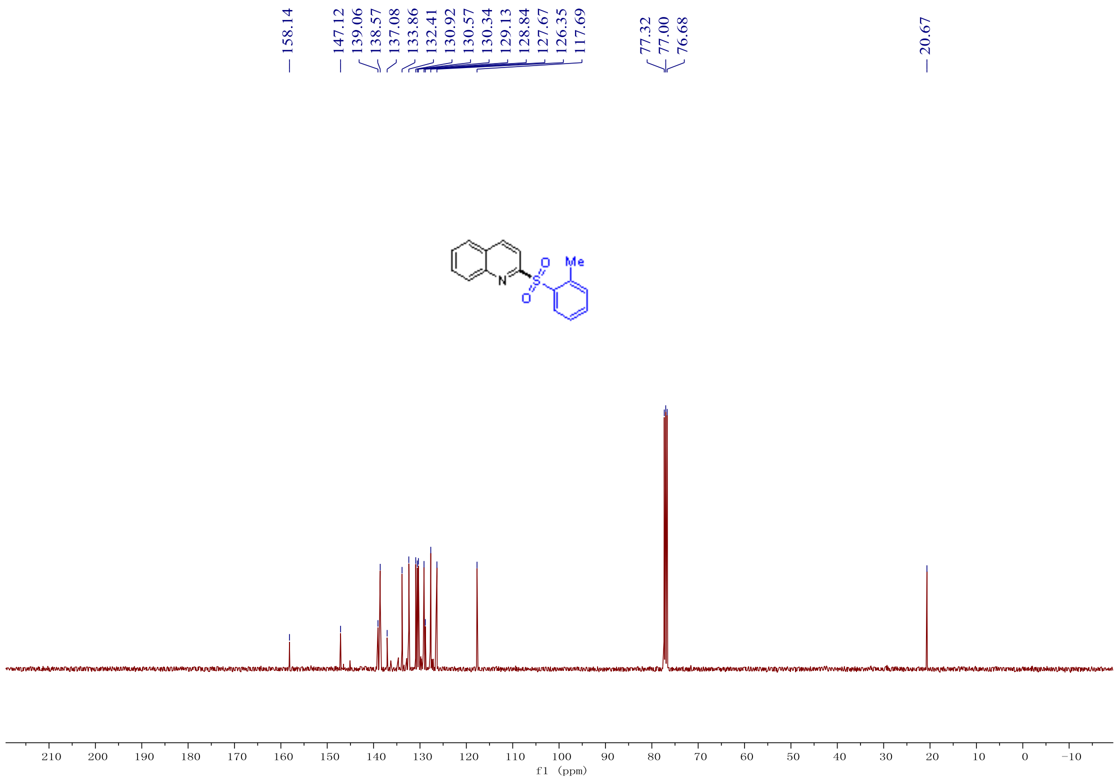

^{13}C NMR ($CDCl_3$, 100 M) spectra of **3am**

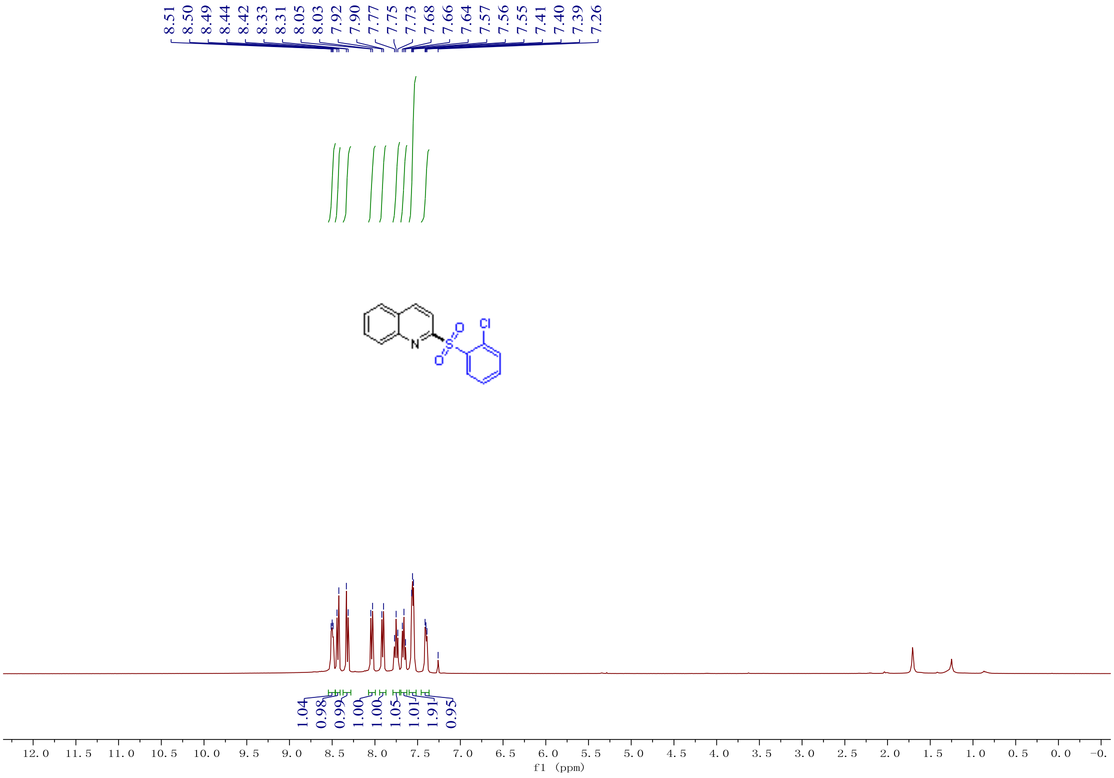

^{1}H NMR ($CDCl_3$, 400 M) spectra of **3an**

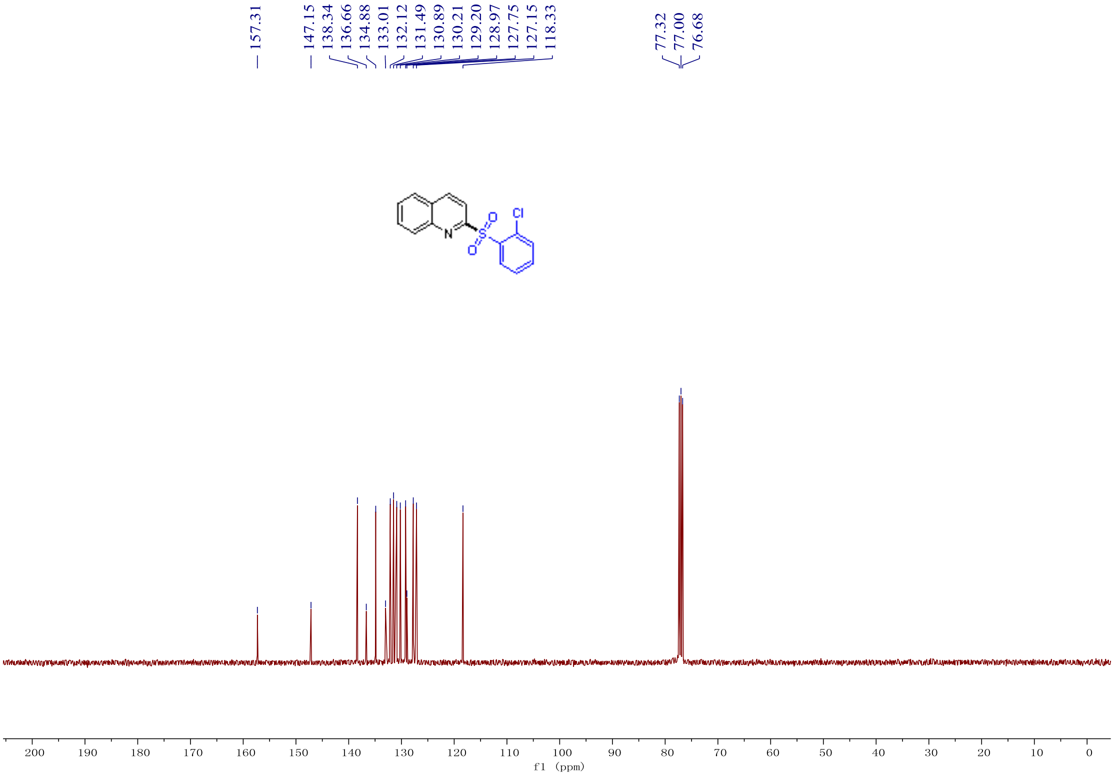

^{13}C NMR ($CDCl_3$, 100 M) spectra of **3an**

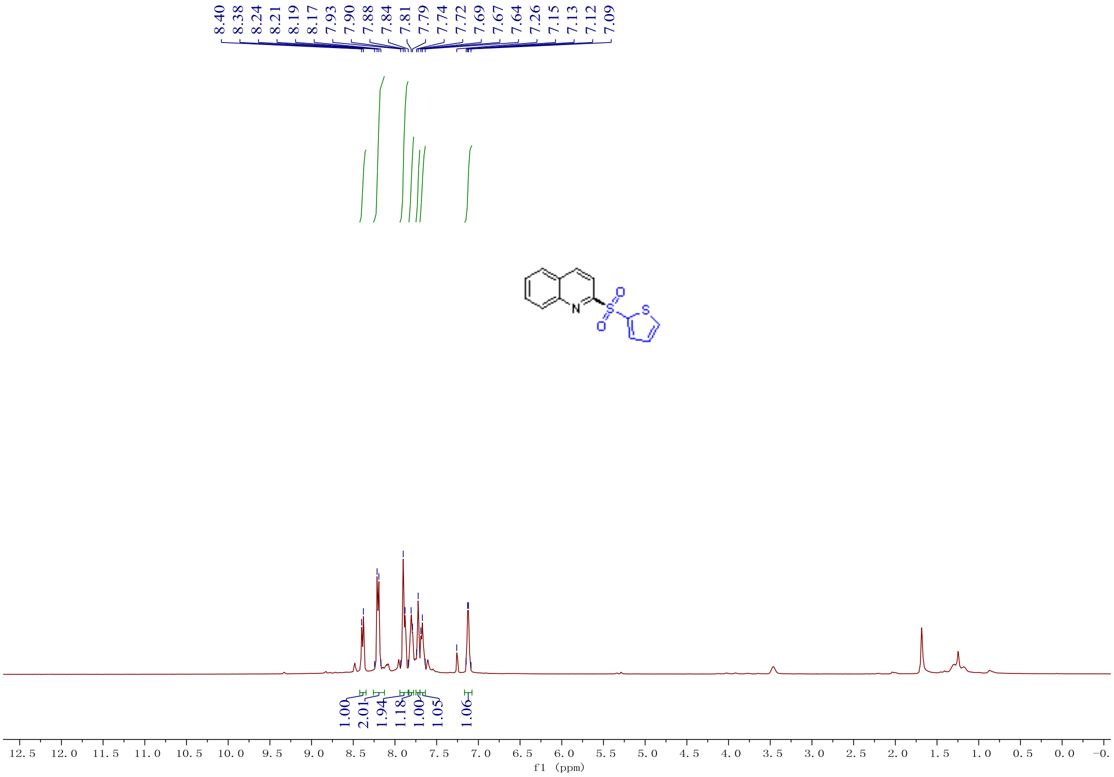

^{1}H NMR ($CDCl_3$, 400 M) spectra of **3ao**

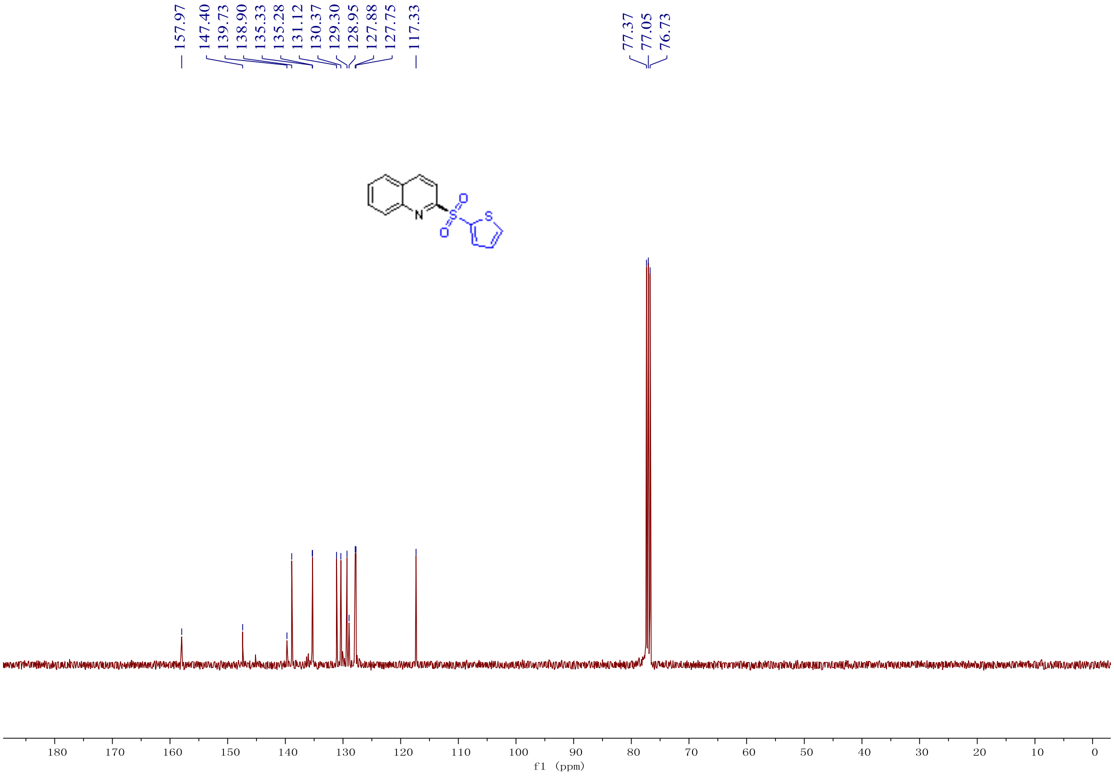

^{13}C NMR ($CDCl_3$, 100 M) spectra of **3ao**

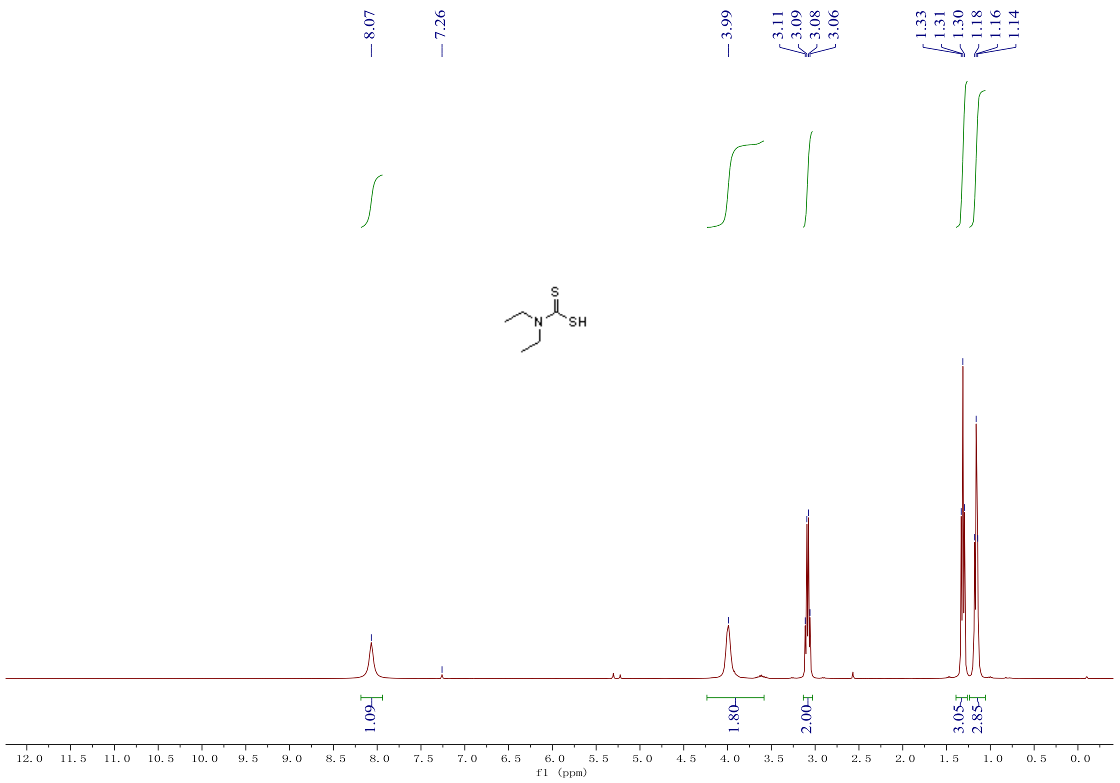

^{1}H NMR ($CDCl_3$, 400 M) spectra of **IM-1**

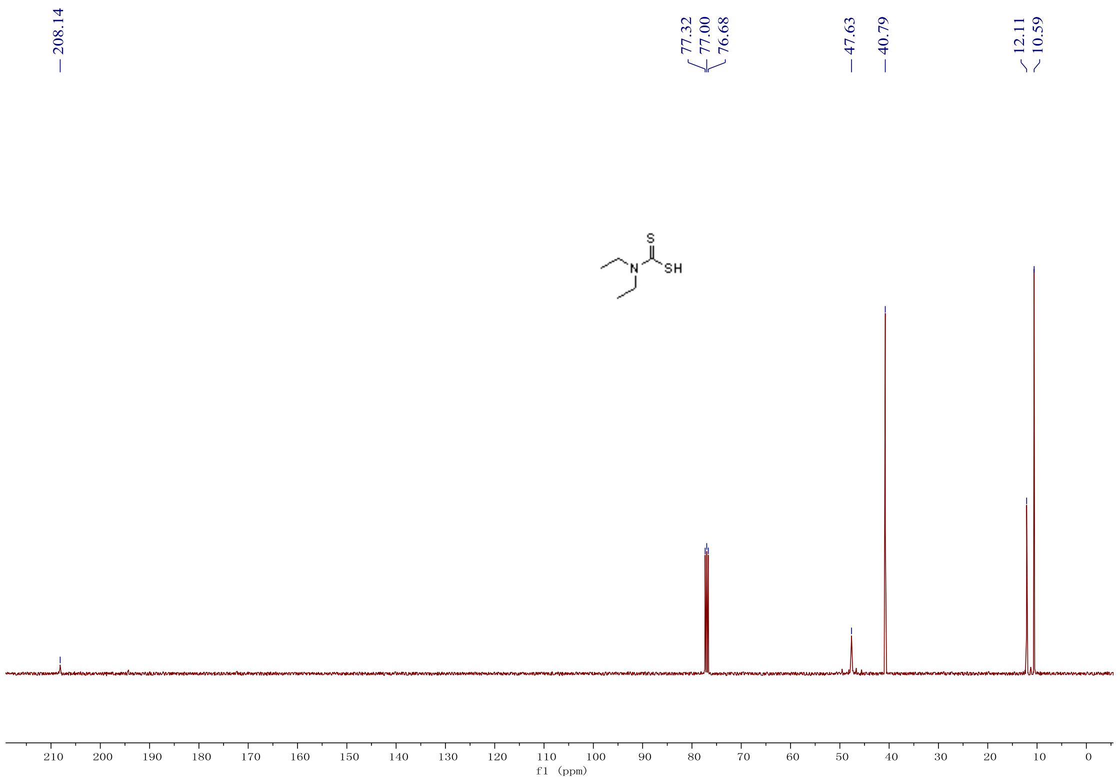

^{13}C NMR ($CDCl_3$, 100 M) spectra of **IM-1**

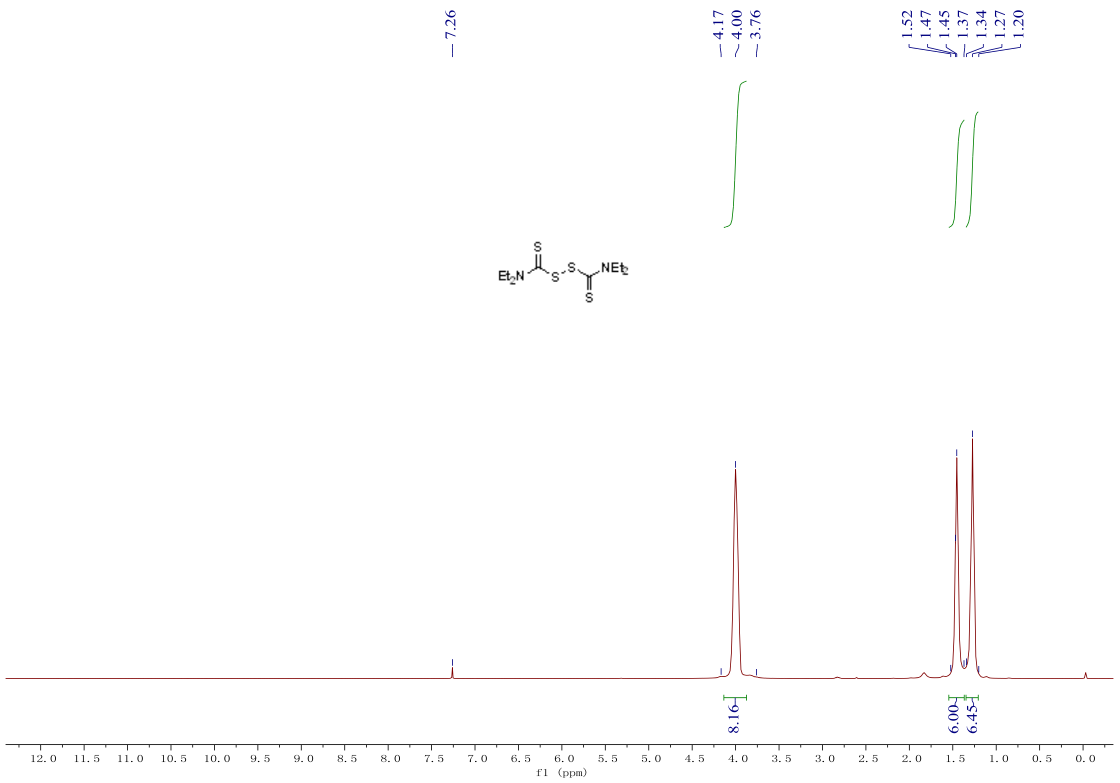

^{1}H NMR ($CDCl_3$, 400 M) spectra of **4a**

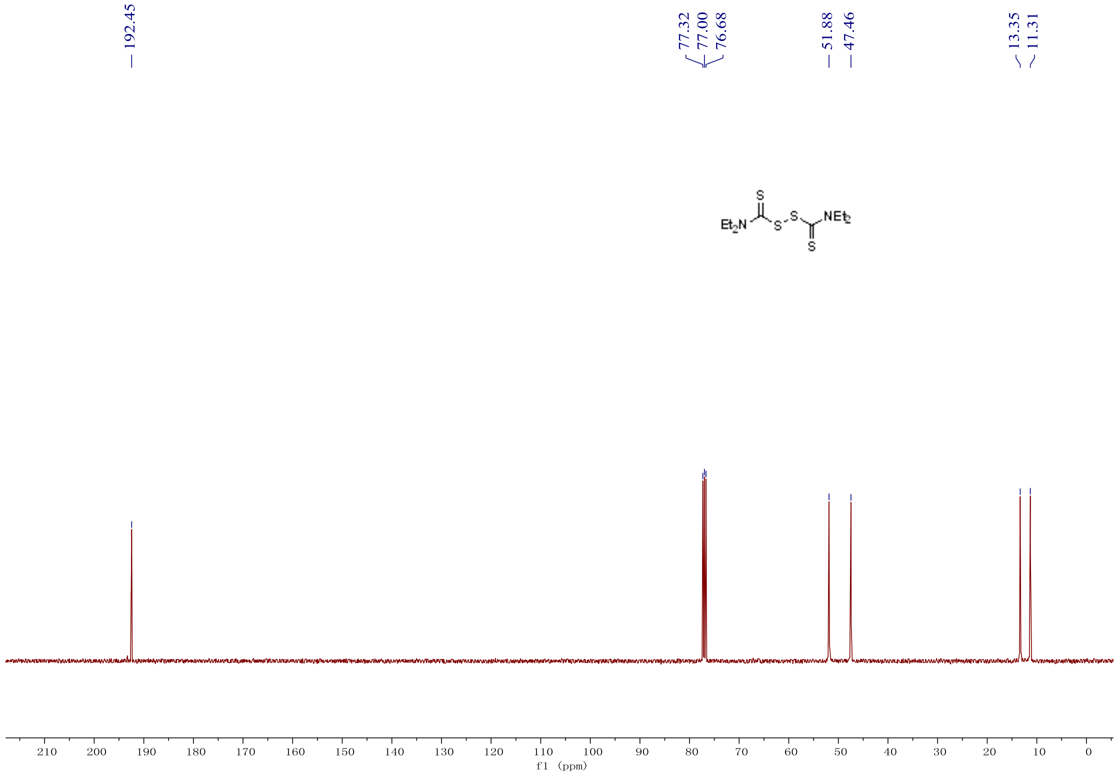

^{13}C NMR ($CDCl_3$, 100 M) spectra of **4a**

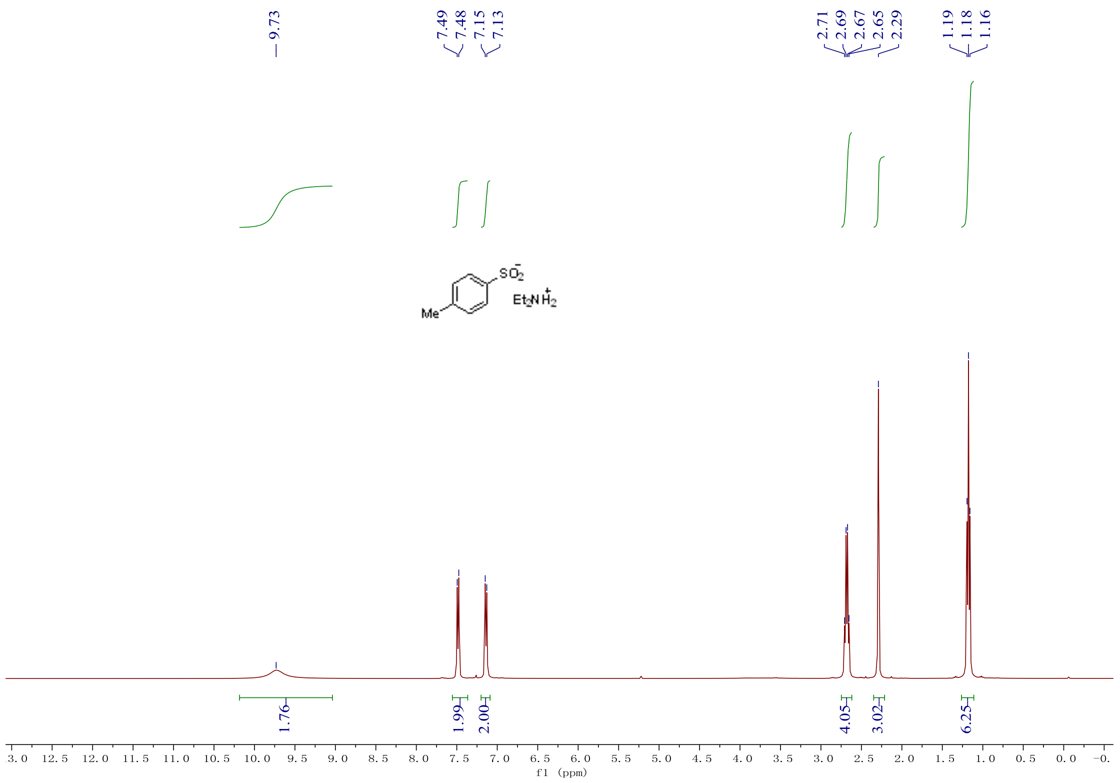

^{1}H NMR ($CDCl_3$, 400 M) spectra of **5a**

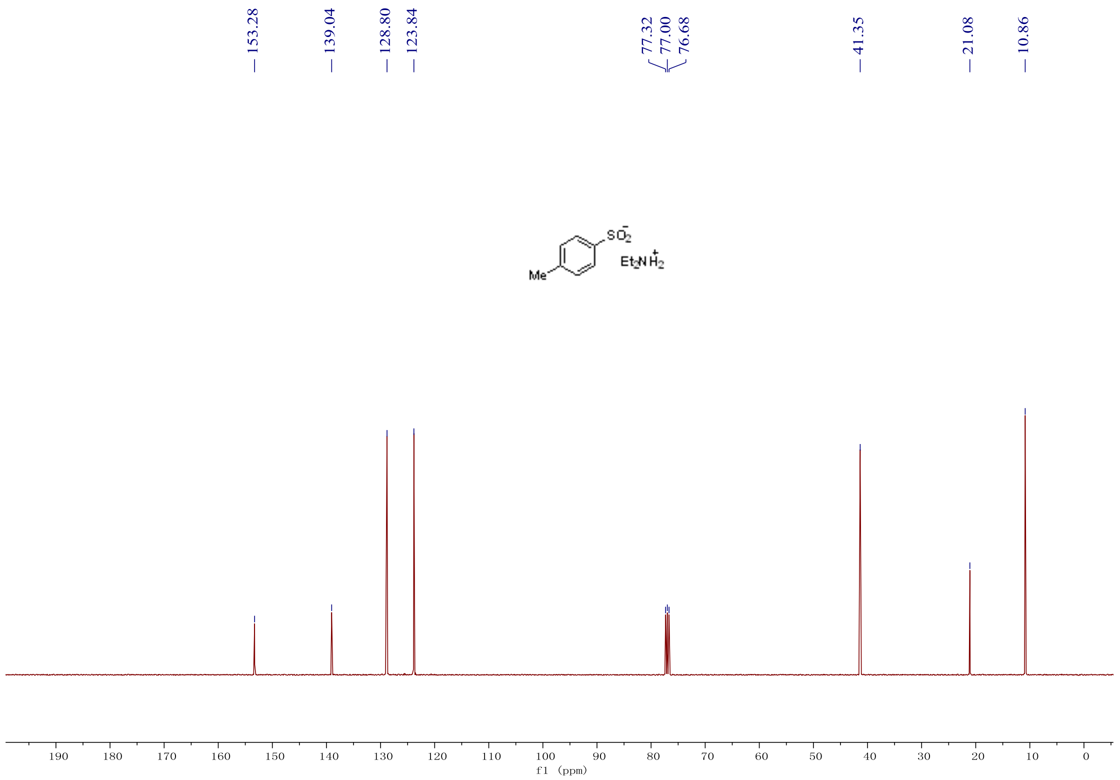

^{13}C NMR ($CDCl_3$, 100 M) spectra of **5a**
